# Metformin Attenuates the High-Glucose Effect on MORG1 Expression in HEK293 Cells

**DOI:** 10.3390/cimb48090934

**Published:** 2026-09-13

**Authors:** Tzvetanka Bondeva, Stefanie Reuter, Anika Westphal, Ralf Mrowka, Gunter Wolf

**Affiliations:** 1 Department of Internal Medicine III, University Hospital Jena, Am Klinikum 1, 07747 Jena, Germany; ralf.mrowka@med.uni-jena.de (R.M.); gunter.wolf@med.uni-jena.de (G.W.); 2 ThIMEDOP, Jena University Hospital, Am Klinikum 1, 07747 Jena, Germany; stefanie.reuter@med.uni-jena.de (S.R.); anika.westphal@med.uni-jena.de (A.W.)

**Keywords:** MORG1 (MAPK organiser protein 1), diabetes mellitus, metformin, CRISPR/Cas9, endogenous luciferase reporter tagging

## Abstract

Reduced expression of MAPK organiser 1 (MORG1) exerts renoprotective effects in MORG1^+/−^ mice in models of type 1 and type 2 diabetes. To investigate the regulation of MORG1 expression, we generated endogenous h*MORG1*-luciferase-tagged HEK293 cells carrying a firefly luciferase reporter inserted downstream of the human *MORG1* gene using the CRISPR/Cas9 method. This study analysed the time-dependent effects of 20 mM D-glucose on MORG1-luciferase activity and examined whether 20 µM metformin influences *MORG1* expression. HEK293 cells were genomically modified using CRISPR/Cas9 to insert the firefly luciferase gene downstream of *MORG1*. Stable clones were selected with puromycin. The influence of D-glucose concentration on MORG1 expression was analysed in a dose- and time-dependent manner. Luciferase assays assessed *MORG1* transcriptional activity, while Western blotting determined protein expression. qRT-PCR was used to assess *MORG1* mRNA levels. The pharmacological effect of metformin was evaluated in parental and tagged HEK293 cells and TKPTS cells, simultaneously exposed to 5.5 mM or 20 mM D-glucose in the presence or absence of 20 µM metformin. High glucose significantly increased luciferase activity and MORG1 protein expression compared with 5.5 mM glucose controls. Time course experiments demonstrated significant induction of *MORG1* transcription, confirmed by corresponding increases in protein levels. Metformin treatment significantly reduced glucose-induced luciferase activity, *MORG1* mRNA levels, and MORG1 protein expression at 24 h. Analyses of TKPTS cells show that 20 mM D-glucose elevated *Morg1* mRNA levels, while this induction was attenuated in the presence of 20 µM metformin. High glucose induces time-dependent upregulation of *MORG1* transcription and protein expression in *MORG1*-luciferase-tagged HEK293 cells. Metformin application attenuates, but does not fully prevent, glucose-induced *MORG1* mRNA and protein expression.

## 1. Introduction

Hyperglycaemia is a hallmark of diabetes and is associated with both type 1 diabetes mellitus (T1DM) and type 2 diabetes mellitus (T2DM) [1]. T1DM is an autoimmune disorder characterised by destruction of pancreatic β-cells, resulting in a deficiency of insulin production [2,3]. In contrast, T2DM is a multifactorial metabolic disorder influenced by genetic predisposition, lifestyle factors, environmental influences, and ageing, which contribute to its development and progression [1,4]. T2DM is characterised by insulin resistance and impaired glucose uptake, ultimately leading to a progressive decline in insulin production by pancreatic β-cells and chronic hyperglycaemia [5,6,7,8].

Diabetic kidney disease (DKD) is one of the most common microvascular complications of diabetes mellitus and remains a leading cause of chronic kidney disease and end-stage renal failure worldwide [9]. Persistent hyperglycaemia promotes renal inflammation, oxidative stress, extracellular matrix accumulation, progressive fibrosis, and tubular atrophy, ultimately leading to declining renal function [10,11,12,13,14,15,16]. Although current therapeutic approaches can slow disease progression, the molecular mechanisms underlying diabetic renal injury remain poorly understood, highlighting the need to identify novel regulatory pathways and therapeutic targets involved in DKD pathogenesis.

We and others have previously identified the MAPK organiser protein 1 (MORG1) as an interaction partner in numerous complexes [17,18,19,20,21]. Therefore, it participates in different cellular signal transduction pathways, but its biological function(s) remain incompletely understood. The review by Loeffler, L. and Wolf, G. [22] summarises the current MORG1 biologically relevant findings on MORG1 [22]. Several lines of evidence suggest that MORG1 is implicated in the pathophysiology of several diseases, including T1DM, T2DM, and hypoxic injury [23]. We have previously demonstrated that reduced MORG1 protein expression in heterozygous *Morg1*-deficient mice exerts renoprotective effects in animal models of T1DM [24], T2DM [25], ischemia–reperfusion [26], and hypoxia-induced kidney injury [23].

The observed beneficial effects in *Morg1* heterozygous mice were associated with reduced expression of inflammatory markers, including kidney injury molecule 1 (KIM-1) [24], as well as decreased renal fibrosis accompanied by lower levels of TGF-β1, CTGF, SNAI1, and α-SMA [23,25,26]. Consequently, *Morg1* heterozygosity improved renal function and attenuated tubular damage compared with wild-type animals subjected to diabetic [25] or hypoxic injury [23]. These studies also measured reduced proteinuria and serum creatinine levels but did not detect reduced glucose levels in these experimental sets.

Despite these findings, the molecular mechanisms by which reduced *Morg1* expression preserves kidney morphology, improves renal function, and limits extracellular matrix accumulation remain poorly understood. At present, the mechanisms underlying these renoprotective abilities in *Morg1*-heterozygous diabetic models are unknown, and future studies are needed to fill this knowledge gap. Thus, the observed renoprotective effects suggest that MORG1 may represent a promising therapeutic target for the prevention or treatment of diabetic kidney disease.

To better understand the regulation of *MORG1* expression, we recently cloned and characterised the human MORG1 promoter in transiently transfected HEK293 [27]. However, transient transfection approaches may reflect reporter activity and do not fully reflect endogenous gene regulation. Therefore, in the present study, we established an endogenous MORG1 reporter system using CRISPR/Cas9-mediated genome editing [28]. Specifically, we generated HEK293 cells with a luciferase reporter tag integrated into the endogenous *MORG1* locus, enabling direct assessment of *MORG1* transcriptional activity under physiological regulatory conditions.

Using this novel reporter system, we investigated the effects of elevated glucose concentrations on MORG1 expression. We demonstrated that high D-glucose levels increase MORG1 expression in both endogenous MORG1-luciferase reporter cells and parental HEK293 cells, suggesting a role for hyperglycaemia in regulating *MORG1* expression. Moreover, a clinically relevant dose of metformin partially reduced D-glucose-dependent *MORG1* mRNA and protein expression.

## 2. Materials and Methods

### 2.1. HEK293 Cell Culture and Treatment Conditions

Human embryonic kidney cells (HEK293 cells) were cultured in Dulbecco’s Modified Eagle Medium containing 4.5 g/L D-glucose (DMEM; Invitrogen, Darmstadt, Germany), supplemented with 10% heat-inactivated FBS (PAN Biotech GmbH, Aidenbach, Germany) and 1% penicillin-streptomycin solution (Gibco™, Thermo Fisher Scientific, Darmstadt, Germany). This is referred to hereafter as complete DMEM.

HEK293 cells were maintained at 37 °C in a humidified atmosphere containing 5% CO2. The cells were passaged at confluence and used for experiments between passages 3 and 8.

To investigate the effects of D-glucose on luciferase activity and on the mRNA and protein expression of MORG1 in endogenously tagged HEK293 cells, the cells were seeded at a density of 2 × 10^5^ cells per well in 12-well plates or 1 × 10^6^ cells per 6 cm cell culture dish for protein analyses and were cultured for 24 h in complete DMEM.

To adapt the cells to 5.5 mM D-glucose and serum deprivation, the culture medium was replaced with DMEM containing 5.5 mM D-glucose, 0.5% heat-inactivated FBS, and 1% penicillin-streptomycin. The cells were incubated under these conditions for an additional 24 h. Following this adaptation period, the cells were exposed to the respective experimental conditions for 24 h.

For dose-dependent analyses, control cells were maintained in DMEM containing 5.5 mM D-glucose, 0.5% heat-inactivated FBS, and 1% penicillin-streptomycin. Experimental groups were treated with additional D-glucose (Carl Roth GmbH + Co. KG, Karlsruhe, Germany) to achieve final concentrations of 10, 20, or 30 mM. Specifically, 4.5, 14.5, or 24.5 mM additional D-glucose was added to the basal medium containing 5.5 mM D-glucose to achieve final concentrations of 10, 20, or 30 mM, respectively. All treatment media contained 0.5% heat-inactivated FBS and 1% penicillin-streptomycin. Cells were incubated under these conditions for 24 h. Subsequently, the cells were lysed using an appropriate lysis buffer for RNA isolation, luciferase assays, or protein expression analysis. Lysates were stored at −20 °C until further analysis.

In addition, the ability of 20 mM D-glucose to modulate *MORG1* mRNA and MORG1 protein expression over time was investigated. Cells were exposed to either control conditions (5.5 mM D-glucose) or 20 mM D-glucose for 3, 6, 9, or 12 h. For the 20 mM D-glucose condition, an additional 14.5 mM D-glucose was added to the basal medium containing 5.5 mM D-glucose to achieve a final concentration of 20 mM. Before experimental treatment, the cells were adapted to a low 5.5 mM D-glucose and low serum condition in medium containing 5.5 mM D-glucose, 0.5% heat-inactivated FBS, and 1% penicillin-streptomycin for 24 h. Following this adaptation period, the cells were exposed to the respective experimental conditions for 3, 6, 9, or 12 h. Afterwards, the cells were lysed using an appropriate lysis buffer for RNA isolation, luciferase assays, or protein expression analysis. Lysates were stored at −20 °C until further analysis.

To evaluate the influence of metformin on the expression of h*MORG1*, the HEK293 cells were seeded as described above. To adapt the cells to 5.5 mM D-glucose and serum deprivation, the culture medium was replaced with DMEM containing 5.5 mM D-glucose, 0.5% heat-inactivated FBS, and 1% penicillin-streptomycin. The cells were incubated under these conditions for an additional 24 h. After that, the cells were exposed to the respective experimental conditions for 24 h. The following treatment groups were used: control group, in which cells were incubated with DMEM containing 5.5 mM D-glucose, 0.5% heat-inactivated FBS, and 1% penicillin-streptomycin; 20 mM D-glucose group, in which cells were incubated with DMEM containing 5.5 mM D-glucose supplemented with an additional 14.5 mM D-glucose to achieve a final concentration of 20 mM, 0.5% heat-inactivated FBS, and 1% penicillin-streptomycin; 5.5 mM D-glucose + 20 µM metformin group, in which cells were treated with DMEM containing 5.5 mM D-glucose, 0.5% heat-inactivated FBS, 1% penicillin-streptomycin, and 20 µM metformin; and 20 mM D-glucose + 20 µM metformin group, in which cells were treated with DMEM containing 5.5 mM D-glucose supplemented with an additional 14.5 mM D-glucose to achieve a final concentration of 20 mM, 0.5% heat-inactivated FBS, 1% penicillin-streptomycin, and 20 µM metformin. After 24 h, the cells were lysed using an appropriate lysis buffer for RNA isolation, luciferase assays, or protein expression analysis. Lysates were stored at −20 °C until further analysis.

In separate experiments, cells were treated with two different HIF prolyl-hydroxylase inhibitors: 3,4-dihydroxybenzoic acid (3,4-DHB; 500 µM) or L-Mimosine (500 µM), both purchased from Sigma-Aldrich (Taufkirchen, Germany). To assess the luciferase activity, the cells were seeded at a density of 2 × 10^5^ cells per well in 12-well plates for luciferase assay analyses in complete DMEM. To adapt the cells to 5.5 mM D-glucose and serum deprivation, the culture medium was replaced with DMEM containing 5.5 mM D-glucose, 0.5% heat-inactivated FBS, and 1% penicillin-streptomycin. The cells were incubated under these conditions for an additional 24 h. Following this adaptation period, the cells were exposed to DMEM containing 5.5 mM D-glucose, 0.5% heat-inactivated FBS, and 1% penicillin-streptomycin, which served as the control group, or to DMEM containing 5.5 mM D-glucose, 0.5% heat-inactivated FBS, and 1% penicillin-streptomycin supplemented with 500 µM 3,4-DHB or 500 µM L-Mimosine for an additional 24 h. Subsequently, the cells were lysed in luciferase lysis buffer and were stored at −20 °C until further analysis.

### 2.2. TKPTS Cells Culturing and Treatment Conditions

TKPTS cells are murine proximal tubular cells (ATCCT^®^, CRL-3361™) (Manassas, VA, USA) and were grown in DMEM 1 g/L D-glucose (DMEM; Invitrogen, Darmstadt, Germany) and 10% heat-inactivated serum (PAN Biotech GmbH, Aidenbach, Germany) supplemented with 1% penicillin-streptomycin solution (Gibco™, Thermo Fisher Scientific, Darmstadt, Germany) and 6.3 µg/mL human insulin solution (Sigma-Aldrich, St. Louis, MO, USA) as recommended by the manufacturer.

For experiments, 3 × 10^5^ cells per well were seeded in a 6-well plate, or 8 × 10^5^ cells in 6 cm cell culture dishes in DMEM containing 5.5 mM D-glucose, 10% heat-inactivated FBS, and 1% penicillin-streptomycin solution without insulin supplement, and they were grown for 24 h in this medium for adaptation to conditions without insulin. Because insulin could confound our experimental conditions by influencing glucose uptake and metformin effects, it was not added to the medium during the treatments. After 24 h, the medium was exchanged, and the cells were serum-deprived in DMEM medium containing 5.5 mM D-glucose, 0.5% heat-inactivated FBS, and 1% penicillin-streptomycin solution. To adapt the cells to 5.5 mM D-glucose and serum deprivation, the culture medium was replaced with DMEM containing 5.5 mM D-glucose, 0.5% heat-inactivated FBS, and 1% penicillin-streptomycin. The cells were incubated under these conditions for an additional 24 h. After that, the cells were exposed to the respective experimental conditions for 24 h. The following treatment groups were used: control group, in which cells were incubated with DMEM containing 5.5 mM D-glucose, 0.5% heat-inactivated FBS, and 1% penicillin-streptomycin; 20 mM D-glucose group, in which cells were incubated with DMEM containing 5.5 mM D-glucose supplemented with an additional 14.5 mM D-glucose to achieve a final concentration of 20 mM, 0.5% heat-inactivated FBS, and 1% penicillin-streptomycin; 5.5 mM D-glucose + 20 µM metformin group, in which cells were treated with DMEM containing 5.5 mM D-glucose, 0.5% heat-inactivated FBS, 1% penicillin-streptomycin, and 20 µM metformin; and 20 mM D-glucose + 20 µM metformin group, in which cells were treated with DMEM containing 5.5 mM D-glucose supplemented with an additional 14.5 mM D-glucose to achieve a final concentration of 20 mM, 0.5% heat-inactivated FBS, 1% penicillin-streptomycin, and 20 µM metformin. After 24 h, the cells were lysed using an appropriate lysis buffer for RNA isolation, luciferase assays, or protein expression analysis. Lysates were stored at −20 °C until further analysis.

### 2.3. Generation of DNA Constructs for Endogenous Human MORG1 Tagging with Firefly Luciferase Reporter Tag

MORG1 (RefSeq NM_001099737.3) was tagged in frame at exon 11 with the gene for firefly *luciferase* using a Cas9 and a specially designed guide sequence 5′-ATGGAGCAGGCTGAAGCCAG-3′ that targets the end of the coding sequence. The guide oligo design was performed by the online guide design tool www.benchling.com. The guide was cloned according to the manufacturer’s instructions into pGuide-it-mNeonGreen (Clontech Laboratory GmbH, Heidelberg, Germany). The stop codon of *MORG1* was deleted, and the *luciferase* gene was linked via a P2A sequence. The flanking homology arms were amplified from genomic DNA of HEK293 cells by PCR. The following primers and included restriction enzyme sites were used for the reaction: MORG1 left arm forward NotI AGCTGCGGCCGCGCATGGTGGTTCACACCTGTAATCCC, MORG1 left arm reverse PacI GTCATTAATTAACTGCTCCATCCTCTGCCTCATAG, MORG1 right arm forward AarI ACGTCCACCTGCGTGCTTAACCACCAACAGGACCAAGGACCG, and MORG1 right arm reverse AscI AGCTGGCGCGCCCTTGCAGATGGGGCAGGGTTGTTG. The homology arm PCR products were integrated into the MV-PGK-Puro-TK vector (Transposagen, Transposagen Biopharmaceuticals (Lexington, KY, USA), which was modified by integrating a cassette via NotI and AsiSI digestion. The cassette contains a P2A-linker sequence flanked by the restriction sites NotI and PacI for left homology arm integration and AarI and AscI for right homology arm integration. The firefly *luciferase* gene was integrated via the restriction enzyme sites HindIII and KpnI. The sequencing results were analysed using the VNTI Advance 9.1 software (Agilent Technologies Deutschland GmbH, Waldbronn, Germany).

### 2.4. Establishment of Endogenous Human MORG1 Tagging with Firefly Luciferase Reporter in HEK293 Cells

To establish endogenous tagging of human *MORG1* with a firefly luciferase reporter gene, 3 × 10^5^ HEK293 cells were seeded in 6-well plates in complete DMEM (see Section 2.1). The following day, the medium was replaced with 1 mL DMEM, containing 1.0 g/L D-glucose supplemented with 0.5% heat-inactivated FBS and penicillin-streptomycin.

For endogenous tagging, the cells were transfected with 2 µg purified plasmid DNA of each construct (Morg_LA_P2A_FFLY_Puro_TK_Morg-RA@6 for firefly luciferase tagging and pMorg-Tagging_Guide_mNeonGreen@6, expressing Cas9 and sgRNA guide sequence), using 6 µL of Lipofectamine 2000 transfection reagent (Thermo Fisher Scientific, Darmstadt, Germany), according to the manufacturer’s instructions. The transfection mixture was incubated in 200 µL of serum-free DMEM for 15 min at room temperature and added gently dropwise to the cells. After 24 h, the medium was replaced with complete DMEM, and the cells were selected using 10 µg/mL of puromycin (Carl Roth, GmbH & Co. KG, Karlsruhe, Germany).

After 10 selection passages, the luciferase activity was assessed. Genomic DNA was isolated from puromycin-selected cells, and successful endogenous tagging was confirmed by PCR amplification, using the following primers: genomic *MORG1* forward: 5′-GAGGAACATGTGGTACAAGAAAAAGACT-3’ and firefly luciferase reverse: 5′-GCTTCATGGCTTTGTGCAGCTG-3′. The amplified fragment was verified by sequencing analysis (Eurofins Genomics, Ebersberg, Germany). The primers were purchased from Invitrogen (Thermo Fisher Scientific, Darmstadt, Germany).

### 2.5. RNA Isolation, cDNA Synthesis, and Quantitative Real-Time PCR

Total RNA was isolated using the NucleoSpin^®^ Tissue Kit (Macherey-Nagel GmbH & Co. KG, Dueren, Germany), according to the manufacturer’s instructions. All steps were performed on ice, and the centrifugation was carried out at 4 °C. The total RNA was eluted with 40 µL of RNase-/DNAse-free water, supplied with the RNA isolation kit. The RNA concentration was determined using a microtiter plate reader (Infinite^®^ 200 PRO, Tecan, Austria).

cDNA was synthesized from 1 µg total RNA using the Promega M-MLV Reverse Transcription system (Promega, Walldorf, Germany) with Oligo (dT)_18_ primers (Invitrogen, Thermo Fisher Scientific, Darmstadt, Germany). The reverse transcription was performed at 37 °C for 60 min, followed by enzyme inactivation at 75 °C for 5 min. The cDNA was stored at −20 °C until use.

Quantitative real-time PCR (qRT-PCR) was performed using the qTOWER 2.2 Real-Time PCR Thermocycler (Analytik Jena AG, Jena, Germany). The gene-specific primers were purchased from Invitrogen, Darmstadt, Germany). The primer sequences used for amplification of the human genes are shown in Supplementary Table S1, while the primer sequences used for amplification of the murine genes are shown in Supplementary Table S2. For mRNA quantification, 1 µL cDNA was analysed using 1 µM of each primer and ORA™ SEE qPCR Green ROXLMastermix (highQU GmbH, Kreichtal, Germany) according to the manufacturer’s instructions.

Relative mRNA expression was quantified by qPCRsoft 4.1 software (Analytik Jena AG, Jena, Germany) and calculated using the ∆∆CT method [29]. The CT values of the gene of interest were normalised to housekeeping gene hypoxanthine guanine-phosphorybosyl-transferase (*HPRT*) CT values (∆CT). The gene expression was calculated as a ratio relative to the control R = 2^−∆∆CT^. All samples analysed by real-time PCR within each experiment were independently cultured and treated and represent independent biological samples, and not technical replicates or repeated measurements of the same biological sample.

### 2.6. Luciferase Assays

After treatment, the cells were washed with 1× sterile PBS and lysed using 1× cell lysis buffer (Promega, Walldorf, Germany). The protein lysates were centrifuged at 13,000 rpm for 15 min at 4 °C. The luciferase activity was measured routinely using 20 µL protein lysate and 100 µL luciferin substrate (Promega, Walldorf, Germany) in luminometer mode with a microtiter plate reader (Infinite^®^ 200 PRO, Tecan, Austria). The luciferase activity was normalised to the total protein content and expressed as fold change relative to cells treated with 5.5 mM D-glucose. All samples included in the luciferase assay analyses were independently cultured and treated biological samples. Thus, each sample represented an independent biological replicate rather than a technical replicate or a repeated measurement of the same biological sample.

### 2.7. Analysis of Protein Expression

The cells were lysed in Complete™ Lysis M buffer (Merck KGaA, Darmstadt, Germany) supplemented with protease inhibitors (Merck KGaA, Darmstadt, Germany). Protein concentrations were determined, and routinely 40 µg of total protein was separated by 10–12% SDS-PAGE, followed by semi-dry transfer onto a polyvinylidene fluoride (PVDF) membrane (Merck KGaA, Darmstadt, Germany). The membranes were blocked in 1× Roti blocking buffer (Carl Roth GmbH & Co. KG, Karlsruhe, Germany) and incubated overnight at 4 °C with the following primary antibodies: anti-MORG1 (custom rabbit polyclonal antibody; 1:1000), generated by Biotrend (Berlin, Germany), anti-VINCULIN (mouse monoclonal antibody; 1:1000) purchased from Merck KGaA, Darmstadt, Germany), and phospho-AMPKα (Thr172) antibody(rabbit monoclonal antibody, 1:500) purchased from Cell Signaling Technology, Danvers, MA, USA. After washing with 1× PBS, the membranes were incubated with the appropriate HRP-conjugated secondary antibodies (1:2000 dilution) purchased from KPL (Sera Care, Gaithersburg, MD, USA). Protein bands were visualised using a chemiluminescence reagent (Revvity Health Sciences, Waltham, MA, USA), and chemiluminescent signals were detected and captured using the iBright™ FL1500 Imaging System (ThermoFisher Scientific, Dreieich, Germany). VINCULIN expression served as the loading control. Band intensities were quantified using Fiji ImageJ-win64 software and normalised to the VINCULIN loading control. Protein expression levels are presented as fold change relative to the expression of the protein of interest in cells treated with 5.5 mM D-glucose. All samples analysed by Western blots represent independent biological samples, which means that all samples within each experiment were independently cultured and treated, and not technical replicates or repeated measurements of the same biological sample.

### 2.8. Determination of Metformin Cytotoxicity in HEK293 Cells and TKPTS

Although metformin has long been used in patient treatment, we performed a cytotoxicity assay to assess whether it exhibits cytotoxic effects under our experimental conditions in HEK293 cells and TKPTS cells. For this purpose, we used the LDH-Cytox™ Assay kit from BioLegend (San Diego, CA, USA) and analysed LDH (lactate dehydrogenase) release into the cell supernatant according to the manufacturer’s instructions. To analyse metformin cytotoxicity in HEK293 cells, 10,000 cells per well were seeded in a 96-well plate in DMEM containing 5.5 mM D-glucose supplemented with 10% heat-inactivated FBS, or in DMEM containing 20 mM D-glucose and 10% heat-inactivated FBS, for 24 h. Metformin cytotoxicity was also measured in murine TKPTS cells using 5000 cells per well for analyses. Afterwards, the same procedures were followed for both cell lines. The cytotoxicity assay was performed exactly under our experimental conditions. Therefore, after 24 h, the medium was exchanged for fresh medium containing 5.5 mM D-glucose and 0.5% FBS, or 20 mM D-glucose and 0.5% FBS, in DMEM, and the cells were incubated for another 24 h under serum deprivation conditions. After that, the cells were left untreated (0 µM metformin) or incubated with 20 µM, 200 µM, 1 mM, or 5 mM metformin for 24 h, respectively, in 5.5 mM D-glucose or in 20 mM D-glucose-containing medium. After that, 20 µL of lysis buffer, provided by the supplier, was added to the high-control wells, and the plates were incubated at 37 °C for 30 min, followed by centrifugation of the plate at 250× *g* for 2 min. The high control represents the maximum release of the LDH, as the cells in these wells were incubated for 30 min at 37 °C with a lysis buffer provided by the supplier to lyse the cells and to release the LDH into the cell supernatant, which corresponds to the maximum cytotoxicity measurement. It was performed as all other treatments in triplicate for 5.5 mM D-glucose-treated cells as well as for 20 mM D-glucose-treated cells. In addition, the low control was also performed. It represents the minimal release from the LDH, and the low-control samples did not differ from the 0 µM metformin samples in our experimental protocol, as metformin was dissolved in sterile water. Afterwards, 100 µL of the supernatant from each well was transferred to a new plate, and 100 µL of assay buffer was added to each well, followed by incubation at room temperature in the dark for 30 min. After 30 min, the reactions were terminated by adding 50 µL of stop solution. Finally, the absorbance of the formazan dye formed during the LDH-catalysed reaction was measured at 490 nm. The formazan absorbance is proportional to the amount of LDH released into the medium, indicating cytotoxicity.

### 2.9. Statistical Analysis

Statistical analyses were performed using SigmaPlot v16 software (Systat, Frankfurt am Main, Germany). Data are presented as box and whisker dot plots, plotted using SigmaPlot v16 software. Multi-group comparisons were analysed using one-way ANOVA or the Kruskal–Wallis test, followed by appropriate post hoc testing. Comparisons involving two independent variables were analysed using two-way ANOVA followed by the Holm–Sidak post hoc test. The Scheirer–Ray–Hare test was used as a non-parametric alternative to a two-way ANOVA for analyses involving two independent variables. The analyses were performed using R version 4.5.0 (R Core Team, 2025), with the rcompanion package (version 2.5.0). All tests used for analyses, the corresponding post hoc tests, and the exact *p*-values are shown in the figure legends. All samples included in the analyses were independently cultured and treated biological samples. Thus, each sample represented an independent biological replicate rather than a technical replicate or a repeated measurement of the same biological sample. A *p*-value of < 0.05 was considered statistically significant. * *p* < 0.05, ** *p* < 0.01, and *** *p* < 0.001.

## 3. Results

### 3.1. Characterisation of the hMORG1-Luciferase Reporter Endogenous Tagging in HEK293 Cells

The genome-editing strategy used to introduce the firefly luciferase gene as a reporter at the genomic hMORG1 locus is described in Section 2.1. We verified the correct integration of the firefly luciferase reporter cassette at the endogenous h*MORG1* locus in HEK293 cells by two independent genomic DNA isolation and sequencing procedures. Primers annealing to genomic regions outside the inserted cassette were used to confirm correct integration of the reporter cassette at the endogenous h*MORG1* locus. The proper integration was analysed by generating a PCR product amplified from genomic DNA using the following forward primer 5′-CATGGTCTAGAAGCAGAGCCAAGAGG-3′ that binds to a sequence upstream from the integration cassette and a reverse primer 5′-GCTTCATGGCTTTGTGCAGCTG-3′ that binds to the luciferase reporter gene. The sequencing results were analysed using the VNTI Advance 9.1 software and the corresponding data are shown in Supplementary Figure S1. Supplementary Tables S3 and S4 present the sequencing results from the forward and reverse primers, respectively. In addition, we verified proper integration of the inserted cassette by PCR amplification from independently isolated genomic DNA from the same polyclonal HEK293-selected population using the primer pair shown in Section 2.3. The amplified PCR product was subcloned into the pJET1.2 vector (TermoFisher Scientific, Darmstadt, Germany), and the purified plasmid DNA was sequenced using the forward and reverse pJET1.2 sequencing primers. The corresponding sequencing results are shown in Supplementary Figure S2 and Table S5 (showing the sequencing results in FASTA format). Both independent analyses of genomic DNA isolated from the polyclonal cell population confirmed correct integration of the reporter cassette at the endogenous h*MORG1* locus. All subsequent experiments were performed using this validated polyclonal cell population.

### 3.2. Validation of the hMORG1-Luciferase Reporter Endogenous Tagging in HEK293 Cells

**Figure 1:**
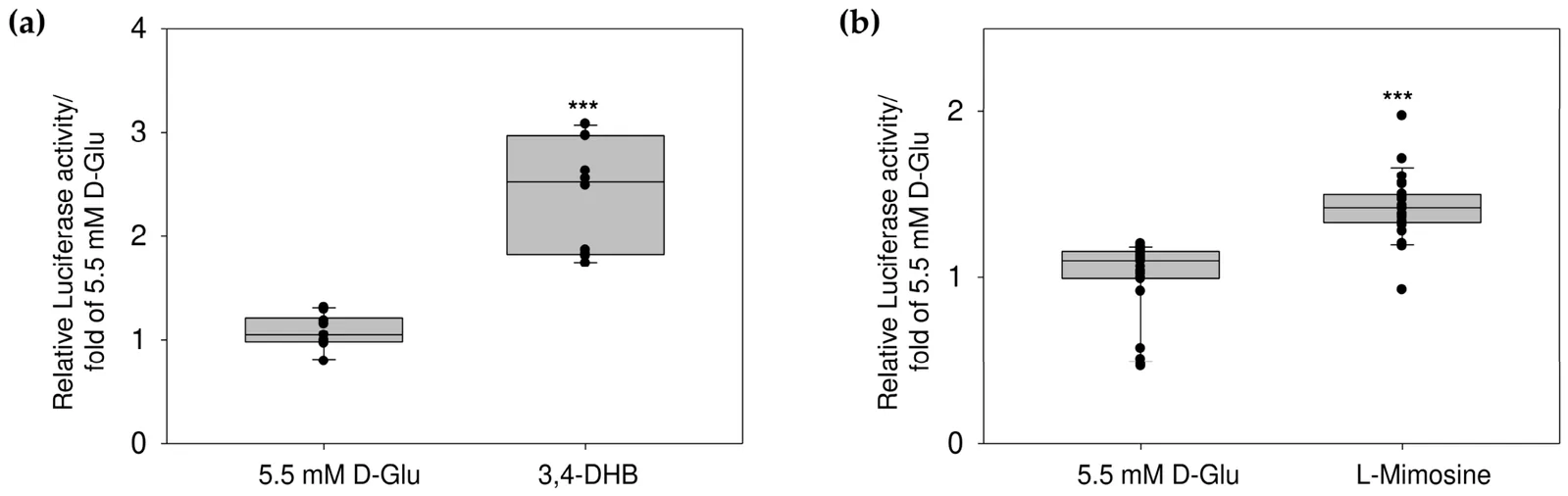
HIF PHD inhibitors induced MORG1-luciferase reporter activity in endogenously tagged HEK293 cells. Analysis of luciferase reporter activity. (**a**) Influence of 3,4-DHB on MORG1-dependent luciferase activity. Data represent 3 independent experiments: 2 experiments with 3 independently treated cell samples per treatment and 1 experiment with 4 independently treated cell samples per treatment (N = 10 biological replicates per treatment). *** *p* < 0.001, as determined by the Kruskal–Wallis test, followed by Dunn’s multiple comparison test. (**b**) Influence of L-Mimosine on MORG1-luciferase reporter activity. Data represent 4 independent experiments, with 6 independently treated cell samples per treatment (N = 24 biological replicates per treatment). *** *p* < 0.001, as analysed by the Kruskal–Wallis test, followed by Dunn’s multiple comparison test.

The goal of the endogenous h*MORG1*-luciferase tagging was to enable the screening of compounds that modulate *MORG1* expression in HEK293 cells. To validate successful endogenous *MORG1*-luciferase reporter integration in HEK293 cells, we investigated the effects of 3,4-dihydroxybenzoic acid (3,4-DHB) and L-Mimosine, two non-specific HIF-prolyl hydroxylase (HIF-PHDs) inhibitors that we previously demonstrated to induce the MORG1-dependent luciferase activity in transiently transfected HEK293 cells [27]. Serum-starved *MORG1*-luciferase-tagged HEK293 cells were either left untreated or stimulated for 24 h with 500 µM 3,4 -DHB or 500 µM L-Mimosine. Following stimulation, the cells were lysed, and the luciferase activity was measured. Our data showed that treatment with 3,4-DHB (Figure 1a) significantly increased luciferase reporter activity compared with untreated control cells. Similarly, L-Mimosine application significantly elevated luciferase activity (Figure 1b).

These observations confirmed our previous results obtained in transient transfection experiments in HEK293 cells and validate the functionality of the endogenously tagged *MORG1*-luciferase reporter system.

### 3.3. Influence of Increased D-Glucose Concentrations on MORG1-Luciferase Reporter Activity in Endogenously Tagged HEK293 Cells

**Figure 2:**
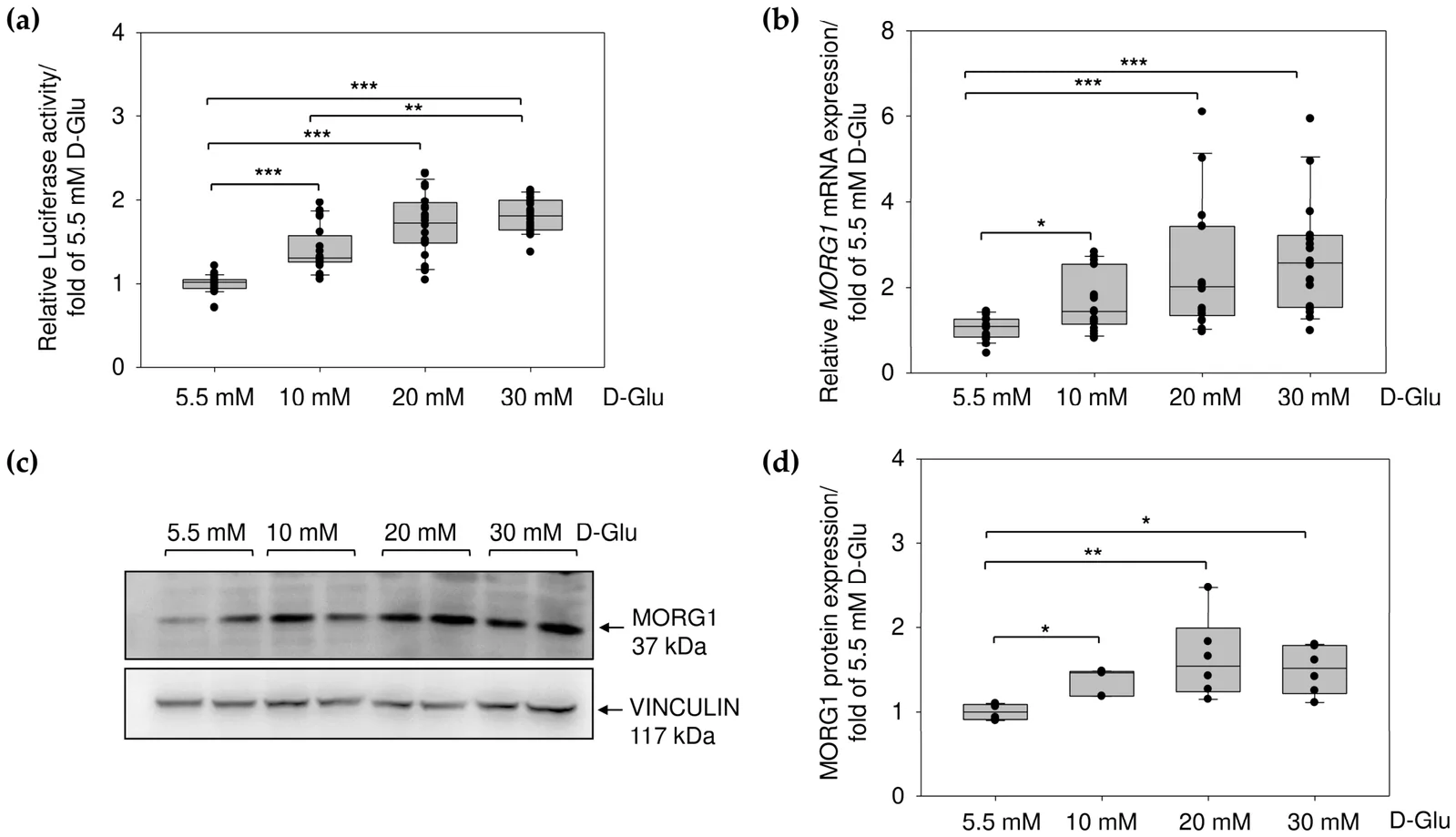
Influence of increased D-glucose concentrations on MORG1 expression in tagged HEK293 cells. *MORG1*-luciferase reporter tagged HEK293 cells were exposed for 24 h to 5.5 mM D-glucose (control condition), or to 10 mM, 20 mM, or 30 mM D-glucose. Afterwards, the cells were lysed and analysed for MORG1 expression using different assays. (**a**) Influence of different D-glucose concentrations on luciferase activity. The assays revealed that elevated D-glucose induced luciferase activity relative to 5.5 mM D-glucose-treated cells. Data represent 4–5 independent experiments, with 6 independently treated samples per treatment. N = 30 (independent biological replicates in the 5.5 mM D-Glu-treated experimental group), N = 24 (independent biological replicates in the 10 mM D-Glu-treated experimental group), N = 24 (independent biological replicates in the 20 mM D-Glu-treated experimental group), N = 30 (independent biological replicates in the 30 mM D-Glu-treated experimental group). *** *p* < 0.001 and ** *p* < 0.01 (** *p* = 0.005), as determined by the Kruskal–Wallis test, followed by all pairwise multiple comparisons using Dunn’s test. (**b**) Influence of different D-glucose concentrations on *MORG1* mRNA expression. Real-time PCR analyses. Data represent 3 independent experiments, with 6 independently treated samples per treatment (N = 18 independent biological replicates per experimental group). * *p* < 0.05 (* *p* = 0.045) and *** *p* < 0.001, as determined by the Kruskal–Wallis test, followed by all pairwise multiple comparisons using Dunn’s test. (**c**) Influence of different D-glucose concentrations on MORG1 protein expression. Representative images of MORG1 and VINCULIN protein expression are shown. VINCULIN protein expression was detected as a loading control. (**d**) Western blot densitometry. Protein expression was measured using ImageJ, and the results are presented graphically relative to 5.5 mM D-glucose-treated cells. Data represent 3 independent experiments, with 2 independently treated cell samples per treatment (N = 6 biological replicates per treatment). ** *p* < 0.01 (** *p* = 0.008); * *p* < 0.01 (* *p* = 0.022, 5.5 mM D-Glu vs. 30 mM D-Glu); and * *p* < 0.01 (* *p* = 0.042, 5.5 mM D-Glu vs. 10 mM D-Glu), as determined by the Kruskal–Wallis test, followed by all pairwise multiple comparisons using Dunn’s test.

Diabetes mellitus is characterised by prolonged periods of hyperglycemia. In previous studies using mouse models of type 1 and type 2 diabetes mellitus (T1DM and T2DM), we demonstrated that reduced *Morg1* expression in heterozygous *Morg1*-deficient mice improved renal function, reduced the albumin to creatinine ratio, and attenuated renal fibrosis [23,26,28]. These findings prompted us to investigate the effects of elevated glucose concentrations on *MORG1* expression using endogenous *MORG1-*luciferase reporter-tagged HEK293 cells. We also assessed whether our endogenously tagged HEK293 cells could serve as a screening platform for substances that modulate *MORG1* expression. Accordingly, we initially treated the cells with a physiological concentration of D-glucose (5.5 mM) or elevated D-glucose concentrations (10 mM, 20 mM, and 30 mM) for 24 h and then assessed luciferase activity. We found that increased D-glucose levels significantly enhanced luciferase activity compared with that in cells treated with 5.5 mM D-glucose (Figure 2a).

Next, we analysed the *MORG1* mRNA expression in tagged HEK293 cells under the same experimental conditions. We found that elevated glucose levels significantly increased *MORG1* mRNA compared with control-treated cells (Figure 2b). The endogenous *MORG1*-luciferase reporter generates a single bicistronic transcript encoding both *MORG1* and the firefly *luciferase* gene, separated by a P2A self-cleaving peptide; thus, two dusting proteins, MORG1 and luciferase, are produced. We, therefore, further examined whether increased glucose levels affect MORG1 protein expression. Western blot analyses revealed that treatment with 10, 20, and 30 mM D-glucose significantly increased MORG1 protein levels relative to physiological 5.5 mM D-glucose conditions (Figure 2c,d).

### 3.4. Influence of Elevated L-Glucose Concentrations on MORG1-Luciferase Reporter Activity in Endogenous Tagged HEK293 Cells

**Figure 3:**
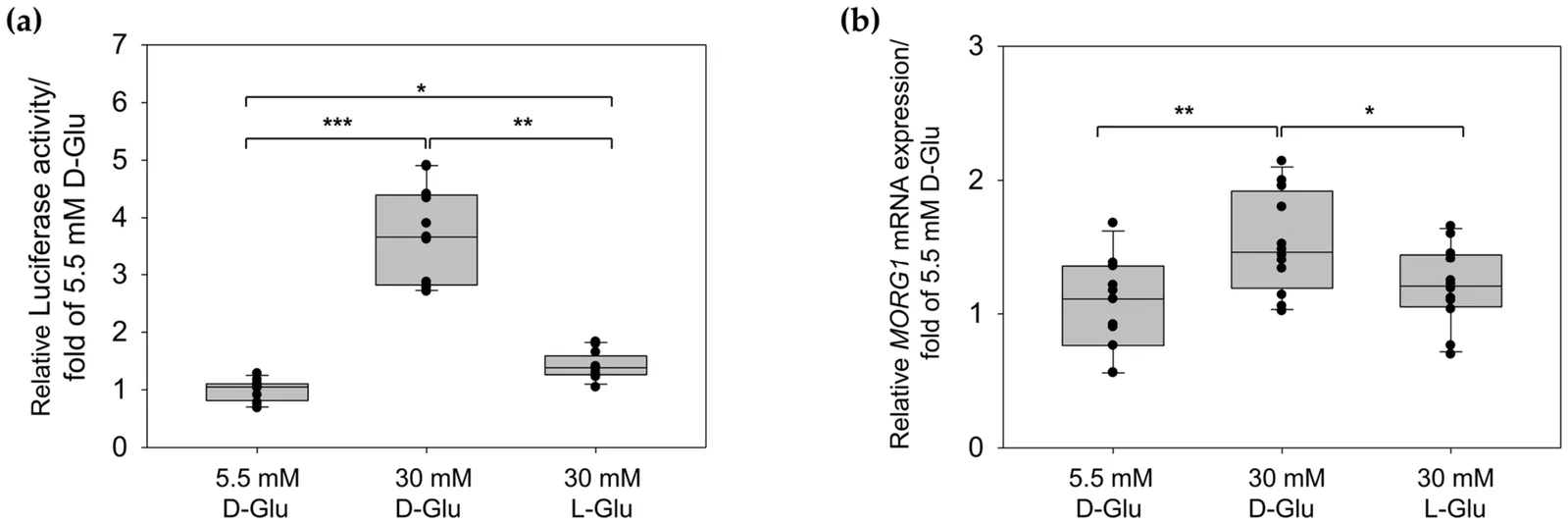
Effects of elevated L-glucose on MORG1 expression in tagged HEK293 cells. *MORG1*-luciferase reporter tagged HEK293 cells were treated with 5.5 mM D-glucose, 30 mM D-glucose, and 30 mM L-Glucose. The 5.5 mM D-glucose was supplemented with 24.5 mM L-glucose to match the osmolarity effect of 30 mM D-glucose for 24 h. The luciferase reporter activity and *MORG1* mRNA expression were analysed. (**a**) Luciferase activity assay from 2 independent experiments, each with 6 independently treated samples per treatment (N = 12 independent biological replicates per experimental group). The luciferase activity is presented as fold relative to the 5.5 mM D-glucose-treated cells. *** *p* < 0.001, ** *p* = 0.01, and * *p* = 0.039, as determined by the Kruskal–Wallis test, followed by all pairwise multiple comparisons using Dunn’s test. (**b**) Real-time PCR analyses of *MORG1* mRNA expression. Data represent 2 independent experiments, with 6 independently treated samples per treatment (N = 12 independent biological replicates per experimental group). The mRNA expression is presented as fold relative to 5.5 mM D-glucose. * *p* < 0.05 (* *p* = 0.030) and ** *p* < 0.01 (** *p* = 0.007), as determined by the one-way ANOVA analyses, followed by all pairwise multiple comparison Student–Newman–Keuls tests.

To exclude the possibility that the effects observed under high D-glucose concentrations were attributed to osmotic changes rather than specific metabolic effects, we treated *MORG1*-luciferase-tagged HEK293 cells with 30 mM L-glucose for 24 h. The luciferase reporter activity and *MORG1* mRNA expression to evaluate the effects of 30 mM D-glucose and 30 mM L-glucose. We observed that treatment with 30 mM D-glucose significantly elevated luciferase activity compared with 30 mM L-glucose and 5.5 mM D-glucose (Figure 3a). Importantly, incubation with 30 mM L-glucose did not increase luciferase activity relative to 5.5 mM D-glucose.

We further examined the effect of 30 mM L-glucose on *MORG1* mRNA expression. Consistent with the luciferase data, 30 mM L-glucose did not induce *MORG1* mRNA expression compared with the cells maintained in physiological D-glucose levels. Moreover, *MORG1* mRNA expression under 30 mM L-glucose was significantly lower than that observed in cells treated with 30 mM D-glucose (Figure 3b). These results demonstrate that the stimulatory effect of elevated D-glucose concentrations on *MORG1* mRNA expression is specific and not attributable to osmotic stress.

### 3.5. Time-Dependent Effect of 20 mM D-Glucose on MORG1-Luciferase Reporter Activity in Tagged HEK293 Cells

To investigate the temporal effects of elevated glucose on *MORG1* expression, we selected 20 mM D-glucose for time course experiments. This concentration reflects glucose levels that may occur in patients with poorly controlled diabetes mellitus.

**Figure 4:**
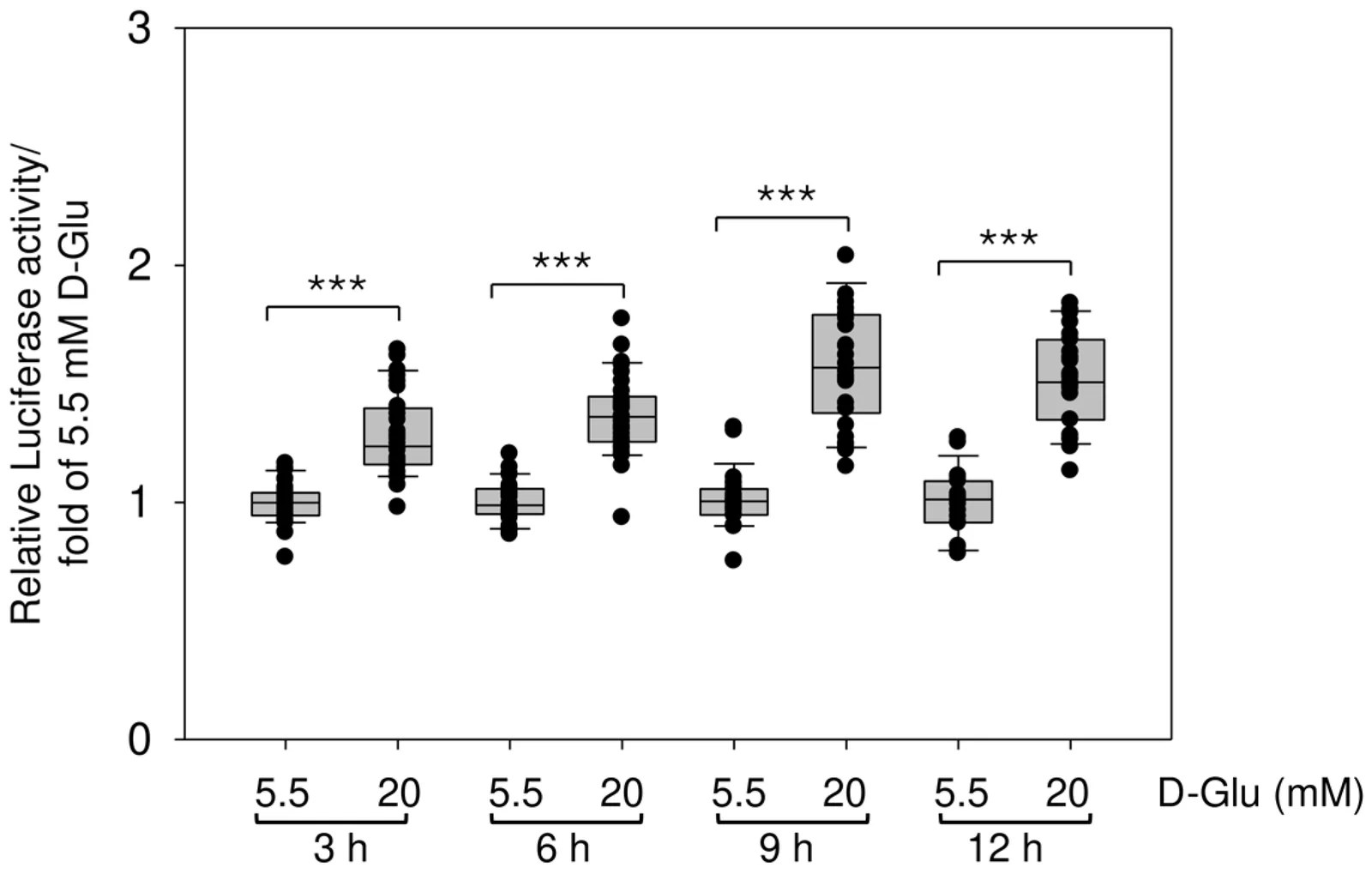
Time-dependent effect of 20 mM D-glucose on MORG1-dependent luciferase activity in *MORG1*-luciferase-tagged HEK293 cells. The cells were stimulated for different time periods with 5.5 mM or 20 mM D-glucose, followed by luciferase activity assays. Luciferase activity after 3 h D-glucose exposure was analysed in 5 independent experiments, each with 6 independently treated cell samples per treatment (N = 30 biological replicates per treatment). *** *p* < 0.001, as determined by a Kruskal–Wallis test followed by Dunn’s test for all pairwise comparisons. Luciferase activity after 6 h D-glucose exposure in 5 independent experiments, each with 6 independently treated cell samples per treatment (N = 30 biological replicates per treatment). *** *p* < 0.001, as determined by the Kruskal–Wallis test followed by Dunn’s test for all pairwise comparisons. Luciferase activity after 9 h D-glucose exposure was analysed in 5 independent experiments (3 experiments with 6 independently treated cell samples per treatment and 2 experiments with 4 independently treated cell samples per treatment) (N = 26 biological replicates per treatment). *** *p* < 0.001, as determined by the Kruskal–Wallis test followed by Dunn’s test for all pairwise comparisons. Luciferase activity after 12 h D-glucose exposure was analysed in 4 independent experiments, each with 5–6 independently treated cell samples per treatment (N = 23 biological replicates per treatment). *** *p* < 0.001 as determined by the Kruskal–Wallis test followed by Dunn’s test for all pairwise comparisons. The complete Kruskal–Wallis test, followed by all pairwise multiple comparisons using Dunn’s test, are shown in Supplementary Table S7.

*MORG1*-luciferase-tagged HEK293 cells were left untreated or incubated with 20 mM D-glucose, and luciferase activity was measured after 3, 6, 9 and 12 h of exposure. Our data were statistically analysed using the Scheirer–Ray–Hare test, a non-parametric method for assessing factorial effects, to evaluate the effects of glucose concentration, incubation time, and their interaction. A statistically significant main effect of glucose concentration was detected (H (1) = 144.553, *p* < 0.001), indicating a significant difference in luciferase activity between the 5.5 mM D-glucose control and the 20 mM D-glucose treatment. In contrast, incubation time did not show a statistically significant main effect (H (3) = 6.318, *p* = 0.097). Furthermore, the glucose concentration × incubation time interaction was not statistically significant (H (3) = 4.771, *p* = 0.189), providing no statistically significant evidence that the effect of glucose concentration varied across the investigated time points. The results of the Scheirer–Ray–Hare analysis are reported in Supplementary Table S6. Furthermore, the differences among the individual glucose concentration–time groups were assessed by a Kruskal–Wallis test followed by Dunn’s test for all pairwise comparisons (shown in Supplementary Table S7). Our results revealed that cells treated with 20 mM D-glucose have a significantly induced MORG1-dependent luciferase activity after 3 h (*** *p* < 0.001), 6 h (*** *p* < 0.001), 9 h (*** *p* < 0.001), and 12 h of exposure (*** *p* < 0.001) relative to cells maintained at physiological 5.5 mM D-glucose levels (Figure 4). The induction was as early as 3 h and persisted for a 12 h observation period, indicating a rapid and sustained transcriptional response to hyperglycaemic conditions.

### 3.6. Time-Dependent Effect of 20 mM D-Glucose on MORG1 mRNA Expression in Tagged HEK293 Cells

**Figure 5:**
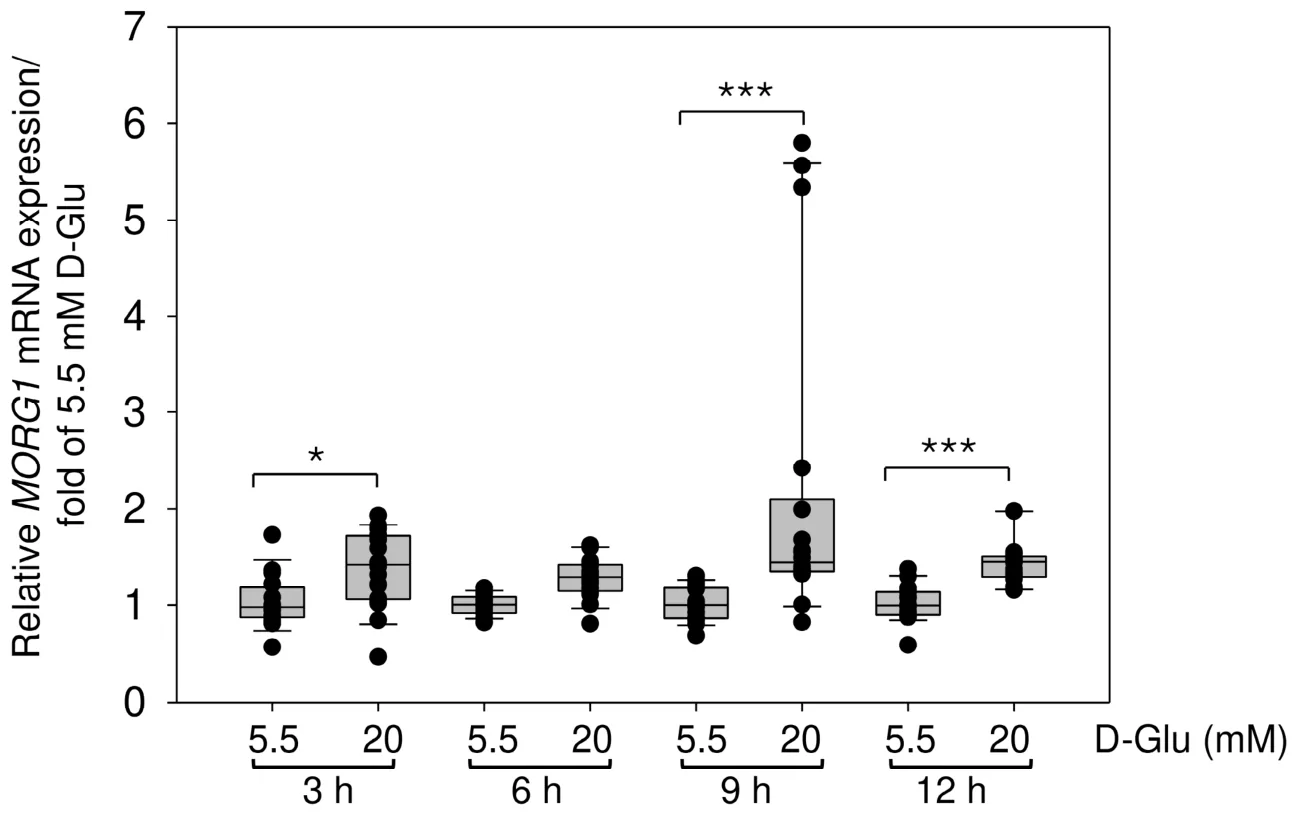
Time-dependent effect of 20 mM D-glucose on *MORG1* mRNA expression in *MORG1*-luciferase reporter-tagged HEK293 cells. Cells were stimulated for different time periods with 5.5 mM or 20 mM D-glucose, followed by RT-PCR analysis of *MORG1* mRNA expression. We analysed *MORG1* mRNA expression after 3 h of D-glucose exposure in 3 independent experiments, each with 4–6 independently treated cell samples per treatment. N = 16 biological replicates per treatment (5.5 mM D-Glu group) and N = 18 biological replicates per treatment (20 mM D-Glu group). * *p* = 0.027, as determined by all pairwise multiple comparisons using Dunn’s test. We analysed *MORG1* mRNA expression after 6 h of D-glucose exposure in 3 independent experiments, each with 5–6 independently treated cell samples per treatment. N = 18 biological replicates per treatment (5.5 mM D-Glu group) and N = 17 biological replicates per treatment (20 mM D-Glu group). No significant difference was detected between cells exposed to 5.5 mM and 20 mM D-glucose. We analysed *MORG1* mRNA expression after 9 h of D-glucose exposure in 3 independent experiments, each with 6 independently treated cell samples per treatment. N = 18 biological replicates per treatment. *** *p* < 0.001. The *MORG1* mRNA expression after 12 h D-glucose exposure was analysed in 3 independent experiments, each with 5–6 independently treated cell samples per treatment. N = 18 biological replicates per treatment (5.5 mM D-Glu group) and N = 17 biological replicates per treatment (20 mM D-Glu group). *** *p* < 0.001. The complete Kruskal–Wallis test, followed by all pairwise multiple comparisons using Dunn’s test, is shown in Supplementary Table S9.

We next investigated whether 20 mM D-glucose similarly affects *MORG1* mRNA expression in *MORG1*-luciferase-tagged HEK293 cells. We treated the cells with 20 mM D-glucose for 3 h, 6 h, 9 h, and 12 h, and measured *MORG1* mRNA levels by real-time PCR. The effects of glucose concentration and incubation time on *MORG1* mRNA expression were assessed using a Scheirer–Ray–Hare test. A Scheirer–Ray–Hare test revealed a statistically significant main effect of glucose concentration (H (1) = 59.125, *p* < 0.001), indicating a significant difference in *MORG1* mRNA expression between the 5.5 mM D-glucose control and the 20 mM D-glucose treatment. In contrast, incubation time did not have a statistically significant main effect (H (3) = 1.958, *p* = 0.581). Furthermore, no statistically significant glucose concentration × incubation time interaction was detected (H (3) = 1.676, *p* = 0.642), indicating that there was no statistically significant evidence that the effect of glucose concentration varied across the investigated time points. The results of the Scheirer–Ray–Hare analysis are reported in Supplementary Table S8. Analyses of the differences among the individual glucose concentration–time groups were determined by a Kruskal–Wallis test followed by Dunn’s all pairwise multiple comparisons method and are shown in Supplementary Table S9. Our data revealed that there was a significant difference in the *MORG1* mRNA expression in cells treated with 20 mM D-glucose after 3 h (* *p* < 0.05), 9 h (*** *p* < 0.001), and 12 h of exposure (*** *p* < 0.001) (Figure 5). We did not measure a significant difference after 6 h of treatment between the cells exposed to 5.5 mM and 20 mM D-glucose.

### 3.7. Time-Dependent Effect of 20 mM D-Glucose on MORG1 Protein Expression in Tagged HEK293 Cells

**Figure 6:**
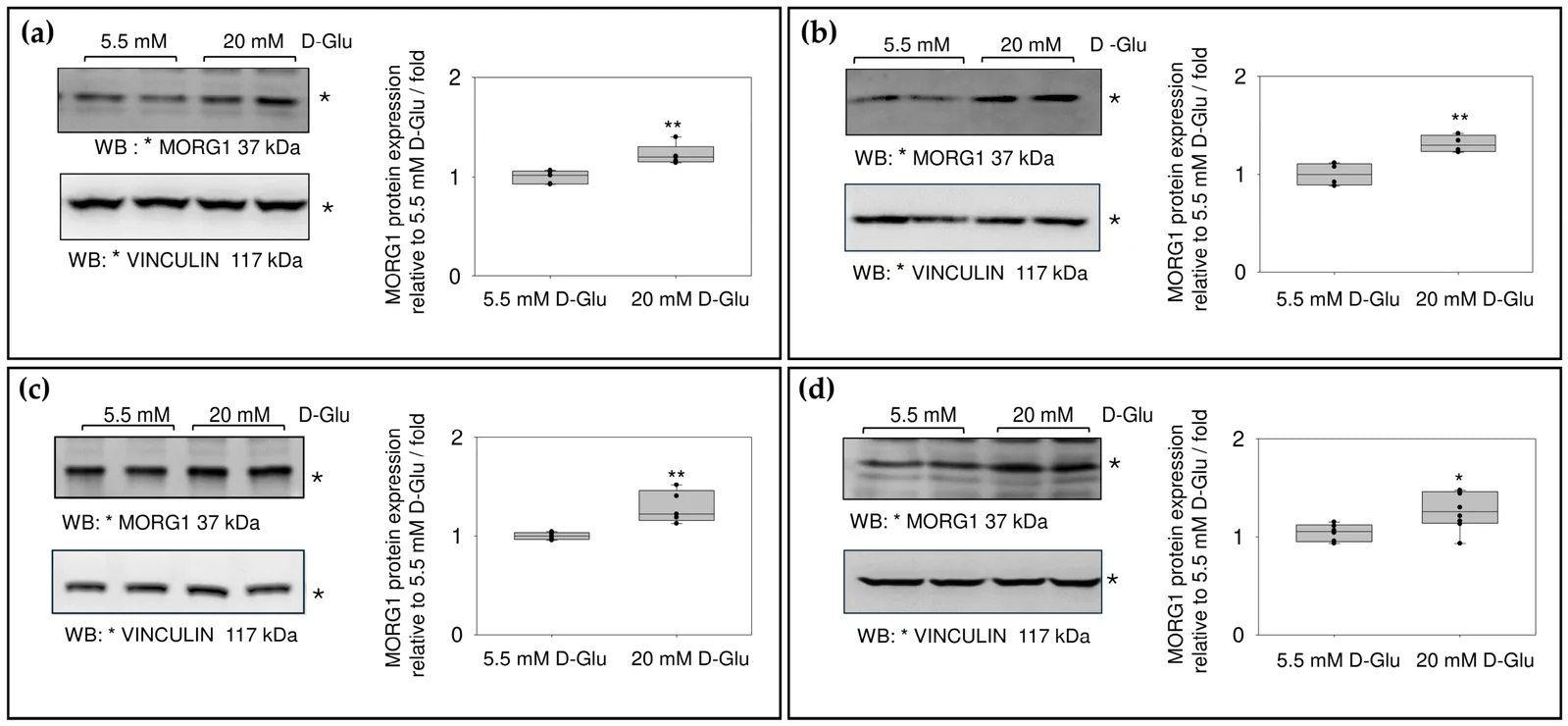
Time-dependent effect of 20 mM D-glucose on MORG1 protein expression in tagged HEK293 cells. The cells were stimulated for 24 h with 5.5 mM or 20 mM D-glucose, respectively, followed by Western blot analysis. (**a**) Detection of the MORG1 protein expression after 3 h of D-glucose exposure. Representative images of MORG1 and VINCULIN protein expression (loading control) are shown. We measured MORG1 protein expression using ImageJ and normalised it to the VINCULIN loading control. Protein expression is presented as fold differences relative to 5.5 mM D-glucose. The graph represents data from 2 independent experiments, each with 2 and 3 independently treated cell samples per treatment (N = 5 biological replicates per treatment). ** *p* = 0.004, as determined by one-way ANOVA, followed by all pairwise multiple comparisons using Tukey’s test. (**b**) Detection of the MORG1 protein expression after 6 h D-glucose exposure. Representative images are shown. MORG1 protein expression was normalised to the VINCULIN loading control and is presented as fold change relative to 5.5 mM D-glucose. The graph represents data from 2 independent experiments, each with 2 independently treated cell samples per treatment (N = 4 biological replicates per treatment). ** *p* = 0.005 as determined by one-way ANOVA, followed by all pairwise multiple comparison Tukey’s test. (**c**) Detection of MORG1 protein expression after 9 h D-glucose exposure. Representative images are shown. MORG1 protein expression was normalised to VINCULIN loading control and is presented as fold change relative to 5.5 mM D-glucose. The graph represents data from 2 independent experiments, with 2 and 3 independently treated cell samples per treatment (N = 5 biological replicates per treatment). ** *p* = 0.005, as determined by one-way ANOVA, followed by all pairwise multiple comparison Tukey’s test. (**d**) Detection of MORG1 protein expression after 12 h of D-glucose exposure. Representative images are shown. MORG1 protein expression was normalised to the VINCULIN loading control and is presented graphically as a fold difference relative to 5.5 mM D-glucose. The graph represents data from 2 independent experiments, each with 2 and 4 independently treated cell samples per treatment (N = 6 biological replicates per treatment for the 5.5 mM D-Glu group and N = 8 biological replicates per treatment for the 20 mM D-Glu group). * *p* = 0.023, as determined by one-way ANOVA, followed by all pairwise multiple comparison Tukey’s test. The asterisk (*) next to the Western blot images indicates the MORG1 or VINCULIN protein; the molecular weight of the proteins is shown under the corresponding representative image.

We further evaluated whether 20 mM D-glucose can elevate the MORG1 protein expression in a time-dependent manner. Accordingly, we treated the cells with 5.5 mM D-glucose or 20 mM D-glucose for the same time periods as for luciferase or real-time PCR analyses, and then detected MORG1 protein expression by immunoblotting. We found that exposure to 20 mM D-glucose for 3 h (Figure 6a), 6 h (Figure 6b), 9 h (Figure 6c), and 12 h (Figure 6d) significantly increased MORG1 protein expression relative to cells treated with normal physiological glucose concentration. Thus, we show that in *MORG1-*luciferase-tagged HEK293 cells, high glucose induced a slight but sustained and prolonged increase in MORG1 protein expression. We note that our analyses were performed with a limited number of samples, and MORG1 protein expression was moderately but significantly elevated under our experimental conditions.

### 3.8. Metformin Reduced the Glucose-Dependent MORG1-Luciferase Activity and MORG1 mRNA Expression in Tagged HEK293 Cells

**Figure 7:**
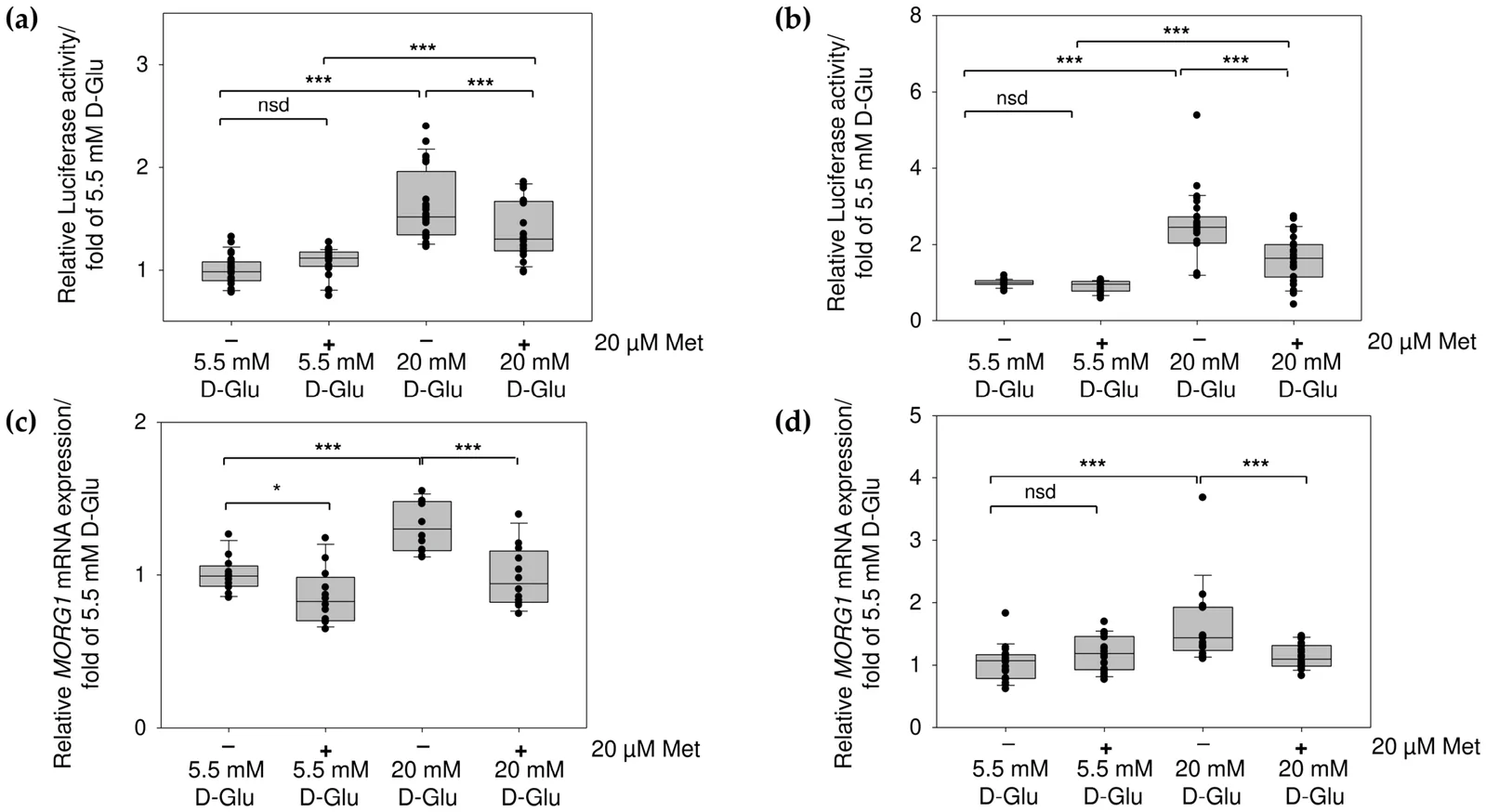
Metformin application reduced the high-glucose-stimulated MORG1-luciferase reporter activity and *MORG1* mRNA expression in *MORG1*-luciferase-tagged HEK293 cells. The cells were stimulated for 12 h and 24 h with 5.5 mM or 20 mM D-glucose in the presence or absence of 20 µM metformin, as shown on the graphs. After that, the cells were lysed and analysed as appropriate. (**a**) Detection of MORG1-luciferase activity after 12 h of treatment. Data represent 4 independent experiments, each with 6 independently treated cell samples per treatment (N = 24 biological replicates per treatment). *** *p* < 0.001, as determined by two-way ANOVA, followed by all pairwise multiple comparisons using the Holm–Sidak method. (**b**) Detection of the MORG1-luciferase reporter activity after 24 h treatments. Data represent 5 independent experiments, 4 experiments with 6 independently treated cell samples per treatment, and 1 experiment with 4 independently treated cell samples per treatment (N = 28 biological replicates per treatment). *** *p* < 0.001, as determined by two-way ANOVA, followed by all pairwise multiple comparisons using the Holm–Sidak method. (**c**) Detection of *MORG1* mRNA expression after 12 h treatments. Data represent 2 independent experiments, each with 6 independently treated cell samples per treatment (N = 12 biological replicates per treatment). * *p* < 0.040 and *** *p* < 0.001, as determined by two-way ANOVA, followed by all pairwise multiple comparisons using the Holm–Sidak method. (**d**) Detection of *MORG1* mRNA expression after 24 h treatments. Data represent 3 independent experiments, each with 5–6 independently treated cell samples per treatment (N = 18 biological replicates per treatment for the following groups, 5.5 mM D-Glu, 5.5 mM D-Glu + 20 µM met, and 20 mM D-Glu + 20 µM met; N = 17 biological replicates per treatment for the 20 mM D-Glu group). *** *p* < 0.001, nsd—not significant, as determined by two-way ANOVA, followed by all pairwise multiple comparisons using the Holm–Sidak method.

Metformin has been a first-line treatment for diabetic patients for decades [30], and it is accepted that it reduces blood glucose levels primarily by inhibiting hepatic gluconeogenesis [31,32]. However, the precise cellular mechanisms of metformin action remained incompletely understood. Clinically, diabetic patients typically receive doses of 1 g/day to 2 g/day, resulting in metformin plasma concentrations of 10–40 µM [33,34,35]. Therefore, to approximate clinically relevant conditions, we treated *MORG1*-luciferase-tagged HEK293 cells with 20 µM metformin for 12 and 24 h, alone or in combination with 20 mM D-glucose, to assess its effects on *MORG1* expression. Analysis of the luciferase activity revealed that 20 µM metformin alone slightly but not significantly elevated the MORG1-luciferase reporter activity after 12 h. In contrast, adding metformin with 20 mM D-glucose significantly reduced luciferase activity after 12 h of treatment compared with 5.5 mM D-glucose (Figure 7a). Furthermore, we analysed metformin effects on *MORG1*-luciferase reporter activity after 24 h in control and 20 mM D-glucose-exposed cells. After 24 h, metformin alone did not significantly alter luciferase activity relative to cells treated with 5.5 mM D-glucose, whereas it markedly attenuated D-glucose-induced luciferase reporter activity relative to cells treated with 20 mM D-glucose (Figure 7b).

We further examined the influence of 20 µM metformin on *MORG1* mRNA expression under the same experimental conditions. Our data showed that metformin alone significantly reduced *MORG1* mRNA levels in cells treated with normal glucose concentrations after 12 h (Figure 7c), but it did not affect mRNA expression after 24 h (Figure 7d). However, it significantly decreased *MORG1* mRNA expression after 12 h (Figure 7c) and 24 h (Figure 7d) treatment compared with 20 mM D-glucose-treated cells.

To exclude the possibility that the observed reduction in MORG1-luciferase activity or mRNA expression is related to metformin cytotoxic cellular effects, we also performed a cytotoxicity assay in cells treated with 5.5 mM D-glucose or 20 mM D-glucose in the presence or absence of 20 µM, 200 µM, 1 mM, or 5 mM metformin for 24 h. Analyses of LDH release showed that under our experimental conditions, these concentrations of metformin did not demonstrate cytotoxic effects on HEK293 cells, neither in 5.5 mM D-glucose nor in 20 mM D-glucose-treated cells, compared with the low control-treated cells (Supplementary Figure S3). On the other hand, we observed a drastic increase in LDH release in high control-treated cells (Supplementary Figure S3).

### 3.9. Metformin Reduced the Glucose-Dependent MORG1 Protein Expression in Tagged HEK293 Cells

**Figure 8:**
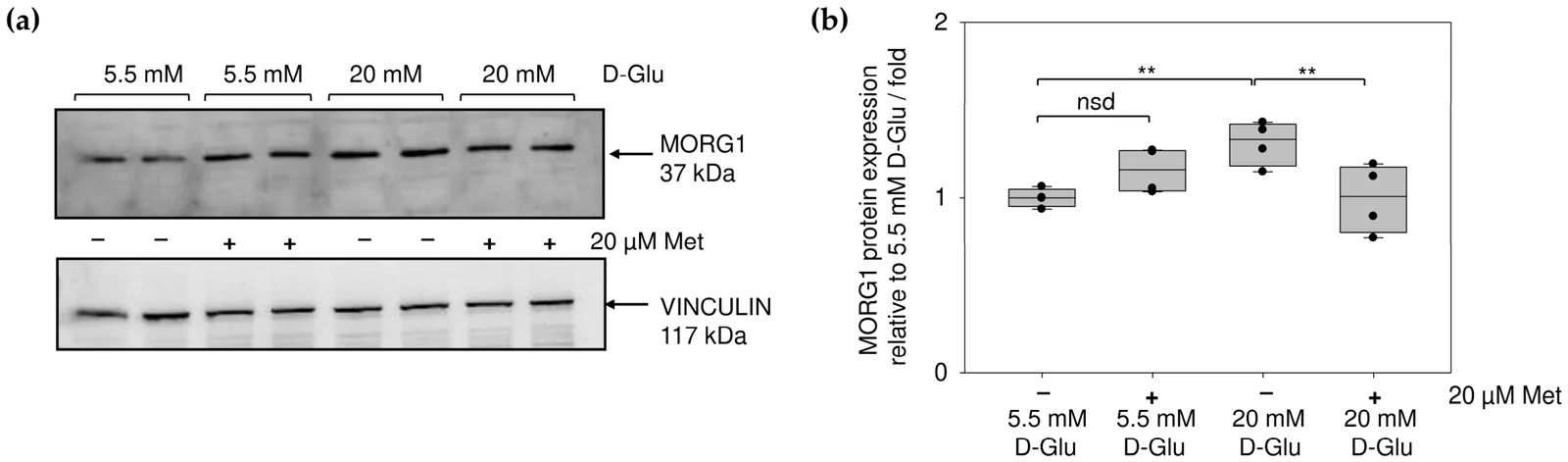
Metformin treatment reduced the high-glucose-dependent increase in MORG1 protein expression in tagged HEK293 cells. Cells were stimulated for 24 h with 5.5 mM or 20 mM D-glucose in the presence or absence of 20 µM metformin, as shown on the graph. Cells were then lysed and analysed by Western blotting. (**a**) Detection of MORG1 protein expression. Representative images from MORG1 and VINCULIN loading control are shown. (**b**) Western blot densitometry. MORG1 protein expression was measured using ImageJ software, normalised to VINCULIN expression, and presented as fold differences relative to 5.5 mM D-glucose. Data represent 2 independent experiments, each with 2 independently treated cell samples per experimental group (N = 4 biological replicates per treatment). ** *p* = 0.006 (20 mM D-Glu vs. 20 mM D-Glu + 20 µM Met) and ** *p* = 0.007 (5.5 mM D-Glu vs. 20 mM D-Glu). Significance shown on the graphs was determined by two-way ANOVA, followed by all pairwise multiple comparisons using the Holm–Sidak method; nsd—no significant difference.

Treatment with 20 µM metformin inhibited luciferase activity in *MORG1*-luciferase-tagged HEK293 cells and reduced *MORG1* mRNA expression. Therefore, we investigated whether 20 µM metformin for 24 h could attenuate the high-glucose-dependent elevation of MORG1 protein expression relative to cells treated with 20 mM D-glucose alone. We found that 20 µM metformin did not affect MORG1 expression in cells cultured under physiological D-glucose levels (Figure 8a,b). However, metformin significantly decreased MORG1 protein expression in cells treated with 20 mM D-glucose compared with cells exposed to 20 mM D-glucose alone (Figure 8a,b).

### 3.10. Dose-Dependent Effect of D-Glucose on MORG1 Expression in Parental HEK293 Cells

**Figure 9:**
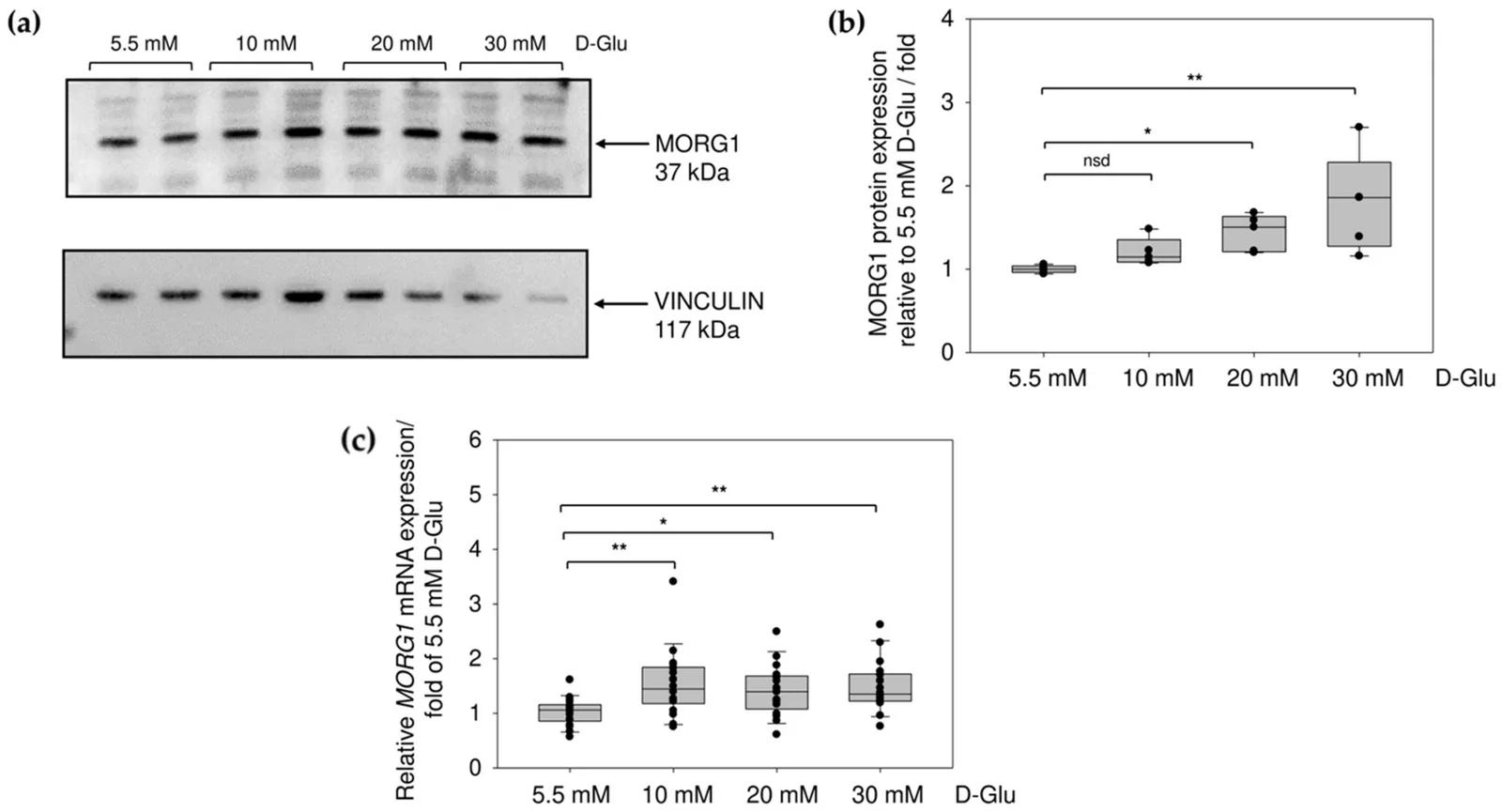
Elevated D-glucose concentrations induced MORG1 protein expression and *MORG1* mRNA expression in parental HEK293 cells. The cells were exposed for 24 h to 5.5 mM (control condition) or to 10 mM, 20 mM or 30 mM D-glucose. After that, the cells were lysed and analysed as appropriate. (**a**) Dose-dependent effect of D-glucose on MORG1 protein expression. Representative images of MORG1 protein expression and VINCULIN protein expression are shown. (**b**) Western blot densitometry. The protein expression was analysed using ImageJ software. MORG1 protein expression was normalised to VINCULIN expression and presented as fold differences relative to control. Data represent 3 independent experiments, each with 2, 2, and 1 independently treated cell samples per experimental group (N = 5 biological replicates per treatment). * *p* < 0.05 (* *p* = 0.023) and ** *p* < 0.01 (** *p* = 0.004), as determined by the Kruskal–Wallis test, followed by all pairwise multiple comparison Dunn’s test. (**c**) Real-time PCR analyses of the dose-dependent effect of D-glucose on *MORG1* mRNA expression in parental HEK293 cells. Data represent 3 independent experiments, each with 6 independently treated cell samples per experiment (N = 18 biological replicates per treatment in 5.5 mM D-Glu, 10 mM D-Glu, and 30 mM D-Glu groups; N = 17 biological replicates per treatment in the 20 mM D-Glu group). * *p* < 0.05 (* *p* = 0.01) and ** *p* < 0.01 (** *p* = 0.005, 5.5 mM D-Gu vs. 10 mM D-Glu). ** *p* < 0.01 (** *p* = 0.006, 5.5 mM D-Gu vs. 30 mM D-Glu), as determined by the Kruskal–Wallis test, followed by all pairwise multiple comparisons using Dunn’s test; nsd—no significant difference.

Based on our findings in endogenously *MORG1*-luciferase reporter-tagged HEK293 cells, we next investigated the effect of elevated D-glucose concentrations on MORG1 protein and *MORG1* mRNA expression in parental HEK293 cells. Our analyses revealed that 10 mM D-glucose increased but not to a significant extent compared with cells exposed to physiological D-glucose levels (Figure 9a,b). However, 20 mM and 30 mM D-glucose significantly increased its expression compared with the control conditions. Furthermore, we did not detect a significant difference in MORG1 protein expression between the higher D-glucose concentrations. We next investigated the influence of increased D-glucose concentrations on *MORG1* mRNA expression by real-time PCR analyses in parental HEK293 cells (Figure 9c). We found that elevated D-glucose concentrations significantly increased *MORG1* mRNA expression relative to control conditions, but we did not detect differences between the different high-glucose treatments. These observations confirm that the effects observed in tagged HEK293 cells are also present in the parental cell line.

### 3.11. Metformin Reduced the D-Glucose-Dependent MORG1 Protein Expression in Parental HEK293 Cells

**Figure 10:**
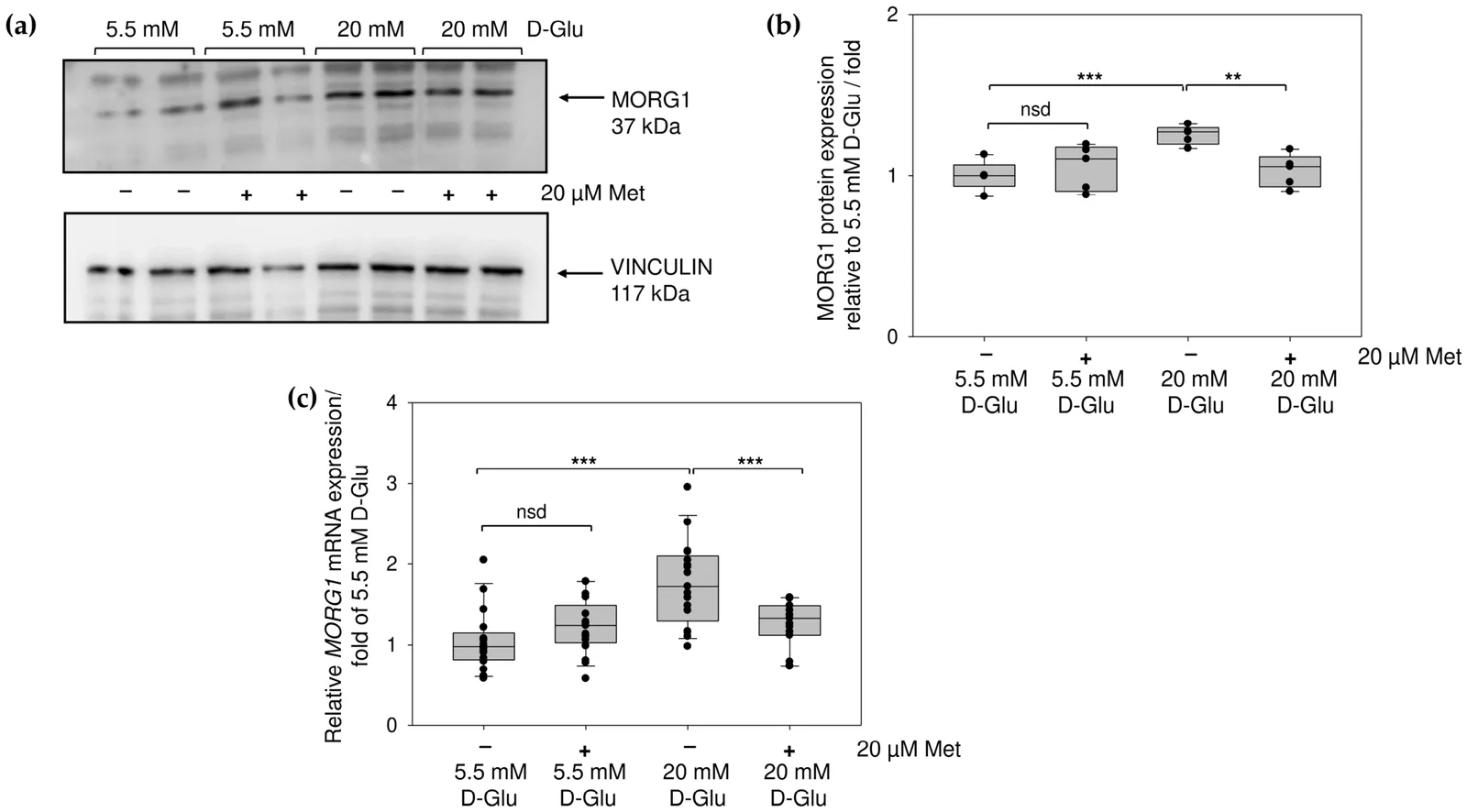
Metformin reduced the MORG1 protein and mRNA expression in parental HEK293 cells. Cells were treated for 24 h with 5.5 mM or 20 mM D-glucose in the presence or absence of 20 µM metformin. Cells were then lysed as appropriate and subjected to further analyses. (**a**) Effect of 20 µM metformin on MORG1 protein expression in HEK293 cells. Protein expression was analysed by Western blotting. Representative images are shown. VINCULIN expression was used as a loading control. (**b**) Western blot densitometry. Densitometry values were normalised to VINCULIN and then to 5.5 mM D-Glu-treated cells. Data represent 3 independent experiments, with 2, 2, and 1 independently treated cell samples per experimental group (N = 5 biological replicates per treatment); ** *p* < 0.01 (** *p* = 0.004). Significance shown on the graphs was evaluated with two-way ANOVA, followed by all pairwise comparisons using the Holm–Sidak method. (**c**) Effect of metformin on *MORG1* mRNA expression in HEK293 cells after 24 h exposure to 5.5 mM or 20 mM D-glucose in the presence or absence of 20 µM metformin. Data represent 3 independent experiments, with 2 × 6 and 1 × 5 independently treated cell samples per treatment (N = 17 biological replicates per treatment in 5.5 mM D-Glu, 5.5 mM D-Glu + 20 µM met, and 20 mM D-Glu treatment groups and N = 15 biological replicates per treatment in 20 mM D-Glu + 20 µM met group), *** *p* < 0.001; nsd—no significant differences. Significance was evaluated using two-way ANOVA, followed by all pairwise comparisons using the Holm–Sidak method.

We next investigated whether 20 µM metformin modulates MORG1 protein and mRNA expression in parental HEK293 cells, as detected in endogenously *MORG1*-luciferase-tagged HEK293 cells. We found that metformin alone did not alter MORG1 protein levels under physiological glucose conditions. However, it significantly reduced high-glucose-dependent MORG1 protein expression when applied simultaneously with 20 mM D-glucose in parental HEK293 cells (Figure 10a,b). Measurement of *MORG1* mRNA levels in parental HEK293 cells showed that metformin significantly attenuated high-glucose-dependent induction of *MORG1* mRNA expression (Figure 10c). These data were consistent with the results from the *MORG1*-luciferase-tagged HEK293 cells.

### 3.12. Influence of Elevated D-Glucose Levels and Metformin on MORG1 Expression in TKPTS Cells

**Figure 11:**
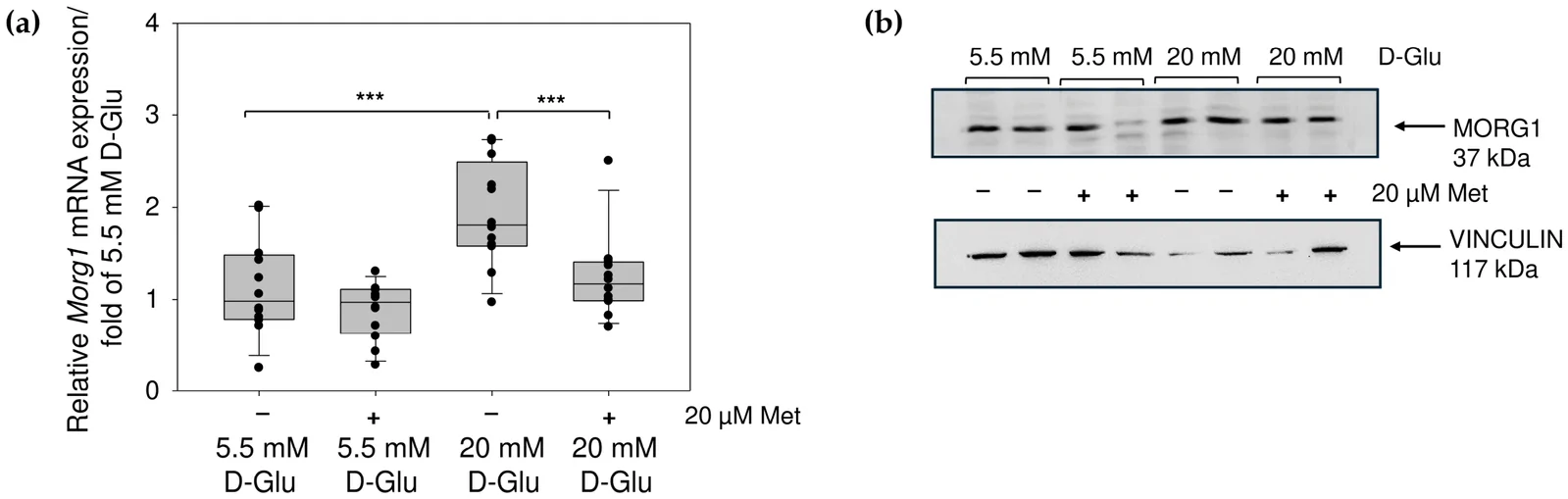
Influence of 20 mM D-glucose and metformin on MORG1 expression in renal proximal tubular TKPTS cells. TKPTS cells were treated with normal 5.5 mM D-glucose or 20 mM D-glucose in the presence or absence of 20 µM metformin for 24 h. Subsequently, cells were lysed, and *Morg1* mRNA expression was evaluated by real-time PCR, while protein levels were assessed by Western blot. (**a**) Influence of 20 mM D-glucose and 20 µM metformin on *Morg1* mRNA expression. Data represent 2 independent experiments, with 6 independently treated cell samples per treatment (N = 12 biological replicates per treatment). Statistical analyses were performed using two-way ANOVA, followed by all pairwise comparisons using the Holm–Sidak method. *** *p* < 0.001. (**b**) Representative images of MORG1 protein expression in TKPTS cells and the loading control VINCULIN.

Because parental HEK293 cells do not have the characteristics of renal proximal tubular cells, we used TKPTS cells, which are murine proximal tubular cells. We exposed them to 5.5 mM and 20 mM D-glucose, with or without 20 µM metformin, for 24 h. Although the suppliers suggested insulin supplementation, we omitted insulin during the stimulation period because it could influence the uptake of D-glucose and metformin into the cells. Our results revealed that *Morg1* mRNA expression was significantly increased in 20 mM D-glucose-treated TKPTS cells compared with 5.5 mM D-glucose-exposed cells (Figure 11a). Furthermore, adding 20 µM metformin to TKPTS cells for 24 h significantly reduced the higher glucose-dependent *Morg1* mRNA levels compared with the expression of *Morg1* in 20 mM D-glucose-treated cells (Figure 11a). We also collected preliminary data on MORG1 protein expression in TKPTS under the influence of elevated concentrations of D-glucose and clinically relevant metformin concentrations. The initial results are shown in Figure 11b. Further analyses are needed to evaluate the role of increased D-glucose on *Morg1* mRNA expression and the functional consequences of elevated MORG1 protein expression. Furthermore, we evaluated whether metformin shows cytotoxic effects on TKPTS cells when treated for 24 h with 20 µM, 200 µM, 1 mM, and 5 mM metformin under normal 5.5 mM D-glucose, as well as at 20 mM D-glucose. As shown in Supplementary Figure S4, metformin treatment does not show cytotoxic effects under our experimental conditions in TPKTS cells.

### 3.13. Influence of Low-Dose Metformin on CTGF and TGF-β1 Expression in HEK293 and TKPTS Cells

Although parental HEK293 are reported to express very low levels of the renal organic cation transporter OCT2 (SLC22A2) and the multidrug and toxin extrusion transporters MATE1 (SLC47A1) and MATE2-K (SLC47A2), making them a widely used host for heterologous transporter expression and functional uptake/efflux assays [36,37,38], we analysed the cells by real-time PCR for the expression of *OCT2* and *MATE-1* in HEK293 cells grown in DMEM medium containing 5.5 mM D-glucose and 0.5% heat-inactivated FBS. We detected low *OCT2* mRNA expression, characterised by an average Ct of 34, while *MATE-1* mRNA expression was characterised by an average Ct of 22.66. Although *OCT2* expression levels were very low, the melting curves were very specific (Supplementary Figure S5). These *OCT2* mRNA levels might be insufficient to support the functional transporter assays. Therefore, we propose that in HEK293 cells, metformin may be transported into the cells independently of OCT2, presumably via OCT3, PMAT, THTR2, SERT, and OCTN2, which represent potential contributors to metformin transport [39].

**Figure 12:**
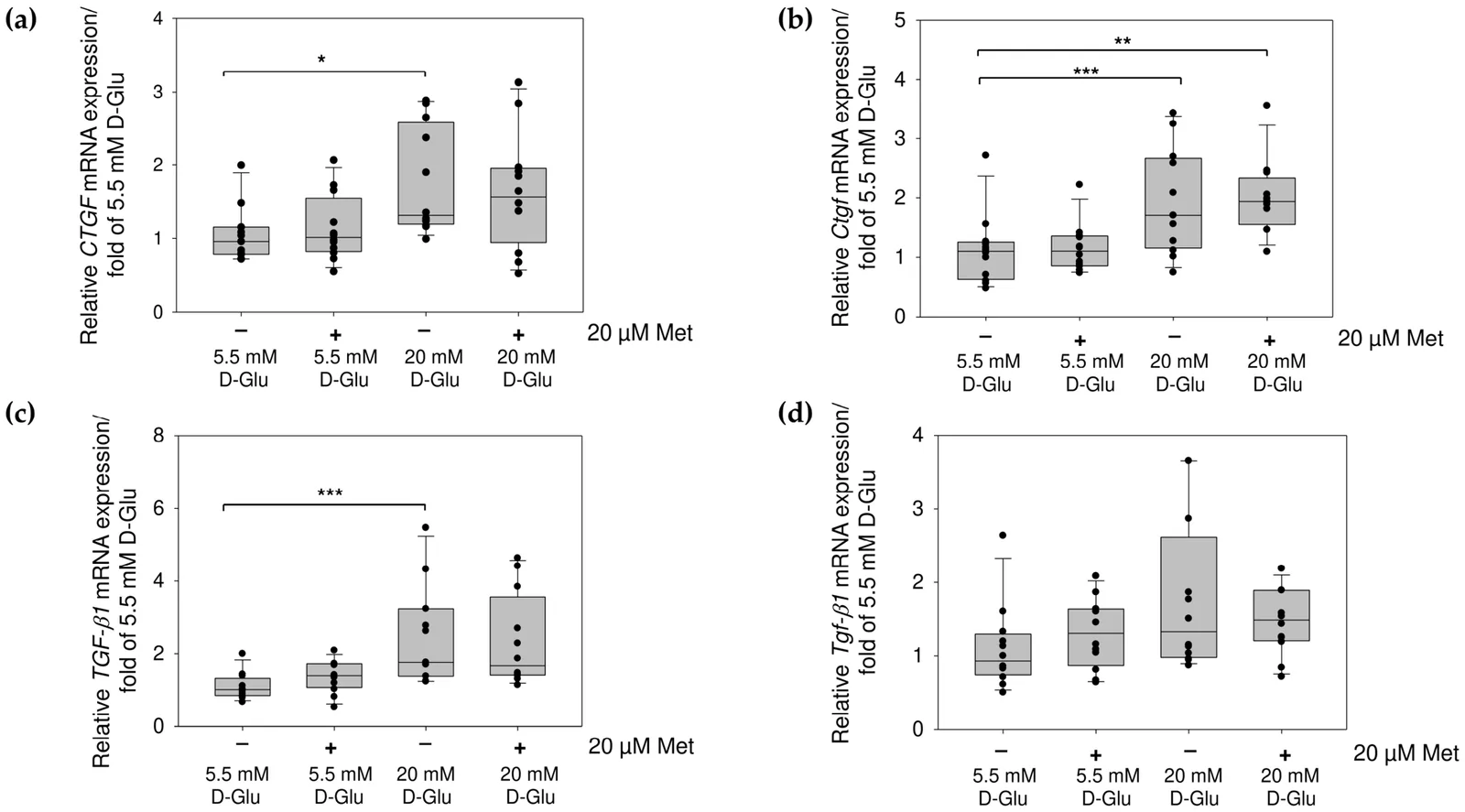
Influence of 20 mM D-glucose and metformin on *CTGF* and *TGF-β1* expression in parental HEK293 cells and in renal proximal tubular TKPTS cells. Cells were exposed to 5.5 mM or 20 mM D-glucose in the presence or absence of 20 µM metformin for 24 h. Total RNA was then isolated, and the corresponding cDNAs were subjected to real-time PCR analyses. (**a**) Detection of *CTGF* mRNA expression in HEK293 cells. Data represent 2 independent experiments, with 6 independently treated cell samples per treatment (N = 12 biological replicates per treatment); * *p* < 0.05 (*p* = 0.012), as determined by two-way ANOVA, followed by all pairwise multiple comparisons using the Holm–Sidak method. (**b**) Detection of the *Ctgf* mRNA expression in TKPTS cells. Data represent 2 independent experiments, with 6 independently treated cell samples per treatment (N = 12 biological replicates per treatment); *** *p* < 0.001. ** *p* < 0.01 (*p* = 0.008), as determined by two-way ANOVA, followed by all pairwise multiple comparisons using the Holm–Sidak method. (**c**) Detection of *TGF-β1* mRNA expression in HEK293 cells. Data represent 2 independent experiments, with 6 independently treated cell samples per treatment (N = 12 biological replicates per treatment). *** *p* < 0.001, as determined by two-way ANOVA, followed by all pairwise multiple comparisons using the Holm–Sidak method. (**d**) Detection of *Tgf-β1* mRNA expression in TKPTS cells. Data represent 2 independent experiments, with 6 independently treated cell samples per treatment (N = 12 biological replicates per treatment). The statistical analyses were evaluated using two-way ANOVA, followed by all pairwise multiple comparisons using the Holm–Sidak method. There was no significant difference between the glucose and metformin treatments.

To identify mechanisms underlying elevated *MORG1* expression in HEK293 cells and TKPTS under high-glucose conditions, and metformin’s partial suppression of this effect, we performed real-time PCR analyses of *CTGF* and *TGF-β1* expression in HEK293 and TKPTS cells. Our data revealed that while 20 mM D-glucose significantly increased *CTGF* expression in HEK293 cells and TKPTS cells (Figure 12a,b) compared with the 5.5 mM D-glucose-treated cells, 20 µM metformin did not affect their mRNA expression. Furthermore, real-time PCR analyses of *TGF-β1* revealed similar effects. We measured increased expression of *TGF-β1* in both cell lines, with a significant increase in HEK23 cells treated with 20 mM D-glucose compared with 5.5 mM D-glucose (*** *p* < 0.001) (Figure 12c,d). In contrast, although metformin treatment decreased *CTGF* and *TGF-β1* mRNA levels, it did not reverse the high-glucose effect in either HEK293 or TKPTS cells under our experimental conditions.

### 3.14. Influence of Low-Dose Metformin on the Phosphorylation of AMPK in HEK293 and TKPTS Cells

It is well documented that higher glucose levels suppressed the phosphorylation and activation of AMPK in many different cellular systems [40]. In addition, there may be a potential connection between AMPK and MORG1, supported by MORG1’s known roles in signalling pathways closely associated with cellular metabolism and stress responses [19]. Therefore, we performed a pilot experiment in HEK293 cells and TKPTS cells and examined the effects of 20 mM D-glucose and 20 µM metformin on AMPK Thr172 phosphorylation (pAMPK) under our experimental conditions. Molnar, Z. et al. [41] demonstrated that metformin can activate pAMPK in HEK293 cells, using Thr172 phosphorylation as a readout for the interaction between high glucose, metformin, AMPK, and mTOR signalling in HEK293 cells [41].

**Figure 13:**
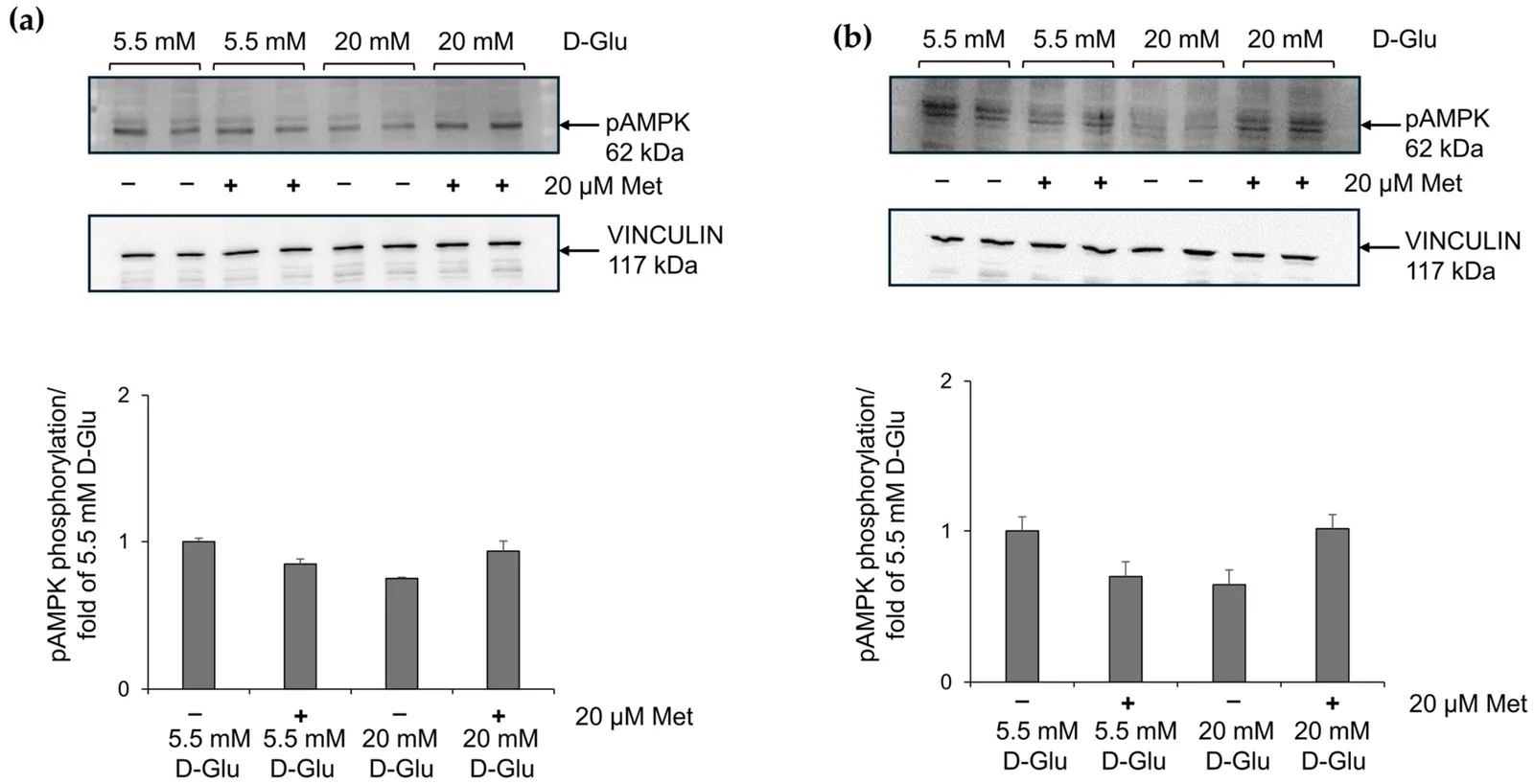
Influence of 20 mM D-glucose and metformin on AMPK Thr172 phosphorylation in parental HEK293 cells and in renal proximal tubular TKPTS cells. Cells were exposed to 5.5 mM or 20 mM D-glucose, with or without 20 µM metformin, for 24 h. After that, total protein was isolated, and the corresponding pAMPK Thr172 was evaluated by Western blot analyses. (**a**) Detection of pAMPK Thr172 in HEK293 cells. Data represent one experiment, with 2 independently treated cell samples per treatment (N = 2 independent biological replicates per treatment). The corresponding pAMPK Thr172 Western blot image is shown. We present VINCULIN expression as a loading control by Western blot, as stripping the corresponding membrane and detecting total AMPK was not reliable for measuring protein expression in ImageJ. (**b**) Detection of the pAMPK Thr172 in HEK293 cells. Data represent one experiment, with 2 independently treated cell samples per treatment (N = 2 independent biological replicates per treatment). The corresponding pAMPK Thr172 Western blot image is shown. We present VINCULIN expression by Western blot as a loading control, as stripping the corresponding membrane followed by detection of total AMPK was not possible to use for measurement in ImageJ.

Based on these findings, we conducted a preliminary experiment using two samples per group, treated under our experimental conditions in HEK293 and TKPTS cells, and investigated the phosphorylated state of Thr172 in AMPK by Western blot analysis. As shown in Figure 13, we observed reduced pAMPK Thr172 phosphorylation in cells treated with 20 mM D-glucose compared with those treated with 5.5 mM D-glucose. In contrast, metformin treatment in the presence of 20 mM D-glucose increased pAMPK Thr172 phosphorylation in both cell types compared with 20 mM D-glucose alone (Figure 13a,b). The total AMPK expression could not be reliably detected by Western blotting because the detection required stripping and re-probing the membrane, and the antibodies against pAMPK and total AMPK were generated in the same host. Therefore, we analysed VINCULIN expression on the same membranes and used it as a loading control for HEK293 cells (Figure 13a) and TKPTS cells (Figure 13b). These preliminary findings require further validation and experiments, including studies in TKPTS cells and animal models of T1DM and T2DM. Further investigation is also required to determine whether AMPK signalling is directly involved in the regulation of MORG1 expression or whether the observed changes reflect an indirect association between these signalling pathways.

## 4. Discussion

Previously, we reported that heterozygous *Morg1*-deficient mice exhibited reduced renal injury in experimental animal models of type 1 and 2 diabetes mellitus [24,25]. Furthermore, these mice displayed attenuated renal damage in models of systemic hypoxia [23] and ischemia–reperfusion [26] and were protected against experimentally induced focal cerebral ischemia compared with wild-type mice [42]. *Morg1* heterozygosity was associated with reduced inflammatory markers, decreased extracellular matrix accumulation, ameliorated renal fibrosis due to lower levels of profibrotic factors, including TGF-β1, CTGF, and SNAI1, and improved renal structure and function in both T1DM and T1DM animal models [24,25]. Collectively, these observations suggested a link between reduced MORG1 expression and improved renal outcomes following diabetic or ischaemic injury.

Therefore, we hypothesised that inhibiting *MORG1* expression may be a therapeutic strategy to ameliorate diabetic kidney injury and preserve renal function in diabetes mellitus. To identify potential inducers or inhibitors of MORG1 expression, we generated and endogenously tagged *MORG1*-luciferase reporter system in HEK293 cells using CRISPR/Cas9-mediated genome editing. This approach enabled monitoring of endogenous *MORG1* transcriptional activation and provided a platform to identify compounds that modulate *MORG1* transcriptional activity through luciferase-based detection.

The initial evaluation of the CRISPR/Cas9-mediated tagged HEK293 cell system was performed by comparing the effects of two non-specific PHD inhibitors on MORG1-luciferase activity, which were already shown to induce MORG1-dependent luciferase activity in transiently transfected parental HEK293 cells [27]. Consistent with our previous findings, both inhibitors induced *MORG1*-luciferase activation in tagged HEK293 cells.

Type 2 diabetes mellitus is associated with elevated D-glucose concentration, a hallmark of the disease [1]. Despite previously observed renoprotective effects associated with reduced *Morg1* expression, little is known about the regulation of *MORG1* under hyperglycaemic conditions. To address this question, we explore newly generated endogenous *MORG1*-luciferase reporter-tagged HEK293 cells. We investigated whether higher glucose concentrations modulate *MORG1* mRNA and protein expression by exposing tagged HEK293 cells to increasing D-glucose concentrations. Our results show that 10, 20, and 30 mM D-glucose increased *MORG1* mRNA and protein expression. We found that high glucose levels significantly augmented luciferase activity, *MORG1* mRNA and protein expression in endogenous-tagged HEK293 cells. Although the magnitude of these effects was modest, *MORG1* mRNA and protein expression increased rapidly. It remained elevated under hyperglycaemic conditions, suggesting that MORG1 may participate in the early cellular adaptation mechanisms triggered by elevated glucose levels.

Importantly, using L-glucose as an osmotic control demonstrated that the observed increase in *MORG1* expression was glucose-specific and not attributed to osmotic stress. This finding indicates that glucose metabolism itself, rather than changes in osmolarity, contributes to the regulation of *MORG1* expression.

Furthermore, in time-dependent experiments using tagged HEK293 cells exposed to 20 mM D-glucose, a concentration frequently observed in patients with poorly controlled diabetes, we observed that *MORG1* transcription was activated within 3 h of glucose exposure and remained elevated for at least 12 and 24 h. These findings suggest that MORG1 may act as an early responder to hyperglycaemia, linking glucose signalling to downstream pathways involved in cellular stress responses or renal pathology. Concordant increases in *MORG1* mRNA, luciferase reporter activity, and protein levels were also detected in parental HEK293 cells. In agreement with these observations, our preliminary data from TKPTS cells treated with 20 mM D-glucose showed increased *Morg1* mRNA expression compared with cells treated with 5.5 mM D-glucose. Nevertheless, further in vivo and in vitro studies are needed to determine the functional consequences of the elevated MORG1 expression in response to increased D-glucose exposure.

Metformin remains a first-line medical treatment for patients with T2DM. Although it has been used for several decades, primarily to reduce blood glucose levels in diabetic patients by inhibiting hepatic gluconeogenesis [31,32,43], its precise cellular mechanisms of action remain incompletely understood. Clinically, diabetic patients typically receive doses of 1 g/day to 2 g/day, resulting in metformin plasma concentrations of 10–40 µM [33,35]. Nevertheless, many studies investigating the cellular effects of metformin use concentrations between 1 and 10 mM, which is difficult to achieve in diabetic patients on metformin [44,45,46]. The discrepancy between the clinical concentrations and those used in cell culture has been explicitly discussed in the literature [47,48,49,50]. The use of millimolar range of metformin concentrations in some studies is largely because a measurable effect of metformin treatment requires concentrations higher than those that are clinically relevant. Studying cellular mechanisms, such as mitochondrial complex inhibition, requires millimolar metformin concentrations [51,52], which are not applicable in patients. Furthermore, metformin, a hydrophilic, positively charged molecule, depends on transporters such as OCT2 for cellular uptake, while in patients, the cells are exposed to metformin within a complex physiological environment [39]. Therefore, the metformin concentrations used matter, as summarised by He, L. and Wondisford, F.E. [47]. In the present study, we aimed to assess the effects of metformin under clinically relevant conditions in HEK293 cells and TKPTS cells. Therefore, we selected 20 µM metformin for our experiments. This concentration falls within the range of metformin plasma concentrations reported in patients receiving therapeutic doses, which are generally in the low micromolar range (~10–40 µM). We were not interested in comparing our study with studies that employed metformin concentrations in the millimolar range, which are considered supra-pharmacological and may not accurately reflect systemic exposure in patients. Our results show that metformin, at clinically relevant concentrations, selectively attenuates glucose-induced MORG1-luciferase reporter activity, *MORG1* mRNA, and MORG1 protein expression after 24 h, without significantly affecting basal levels under physiological glucose conditions. This effect was observed in both *MORG1*-luciferase-tagged and parental HEK293 cells, suggesting that metformin may interfere with glucose-induced signalling pathways rather than directly downregulating *MORG1* expression under normal physiological conditions. This aligned with known pleiotropic effects of metformin, including inhibition of cellular glucose levels by modulation of stress-responsive signalling pathways. Previous studies have shown that physiological D-glucose levels enhanced metformin cytotoxic effects in cancer cells compared with 25 mM D-glucose-exposed cells [53]. The authors reported that in MDAMB2341, MCF7, and SKBR3 breast cancer cells, increasing metformin concentrations were associated with increased cytotoxic effects when the cells were grown under lower D-glucose levels compared with cells exposed to 10 mM or 25 mM D-glucose.

Furthermore, 8 mM metformin reduced ATP levels in low-glucose-treated cells but did not affect MDAMB2341, MCF7, or PA-I cells exposed to 25 mM D-glucose [53]. Several groups have reported that metformin’s anti-cancer effects are associated with increased cytotoxicity at low glucose concentrations [50,54,55,56]. Although we analysed possible cytotoxic effects of 20 µM, 200 µM, 1 mM, and 5 mM metformin in HEK293 cells and TKPTS cells under 5.5 mM or 20 mM D-glucose exposure, we did not detect a cytotoxic effect of metformin in either HEK293 or TKPTS cells. These observations suggest that metformin’s mechanisms of action in healthy cells and tissues may differ substantially from those operating in cancer cells and tissues.

In this regard, it would be of interest to study MORG1 expression in cancer cells and to evaluate the role of metformin-mediated regulation of MORG1 expression in cancer progression.

Several studies have demonstrated antifibrotic effects of metformin in cellular, animal, and clinical settings, associated with reduced expression of TGF-β1, TNF-α, and α-SMA [45,57,58,59,60]. These observations are reminiscent of our previous findings in heterozygous *Morg1*-deficient mice, which also exhibited reduced expression of profibrotic markers and attenuated renal fibrosis [25]. Given MORG1’s participation in multiple signalling pathways, including ERK1/2 [17], and its as-yet-undefined physiological function, the finding of Shen et al. [58] that administration of metformin in animal and cellular experiments inhibited ERK signalling activation and attenuated renal fibrosis [58] could link the reduced renal fibrosis observed in heterozygous *Morg1* mice [25] to decreased *MORG1* expression. Furthermore, a recent study reported that metformin treatment in adult Wistar male rats induced autophagy via activation of the AMPK signalling pathway, which showed a protective effect against kidney ischaemia–reperfusion-induced injury in rats [61]. In previous studies analysing the I/R model in male mice, we found that *Morg1* heterozygous male animals were protected against I/R-related renal injury compared with wild-type mice [26]. Reduced AMP-activated protein kinase phosphorylation (pAMPK) is characteristic of diabetic disease, and increased pAMPK levels are a promising target for controlling or improving T2DM-related complications [62,63,64,65,66]. Recent evidence has identified MORG1 as an important regulator of mTORC1 signalling and autophagy. Abudu et al. demonstrated that MORG1 interacts with active Rag GTPases and limits their ability to recruit mTORC1 to the lysosomal membrane, thereby restricting mTORC1 activation [19]. *MORG1* depletion resulted in increased mTORC1 activity and reduced basal autophagy, indicating that MORG1 promotes basal autophagy indirectly through suppression of mTORC1 signalling [19]. These findings are of particular interest in the context of our observations, given that reduced *Morg1* expression was associated with renoprotection in our experimental models. Moreover, because mTORC1 is a central regulator of cellular responses to nutrient availability and metabolic stress, the glucose-dependent regulation of *MORG1* mRNA and protein expressionobserved in our study may have functional consequences for mTORC1 activity and autophagy. In this regard, metformin activates AMPK, which can suppress mTORC1 signalling and promote autophagy. Thus, the observation that metformin selectively attenuated glucose-induced *MORG1* mRNA and protein expression raises the possibility that MORG1 may participate in the metabolic signalling network connecting glucose availability, AMPK, mTORC1, and autophagy. However, this hypothesis requires experimental validation, particularly because we did not directly assess AMPK or mTORC1 activity in the present study.

In contrast, when analysing the effect of metformin on *MORG1* expression, we observed that a general suppression of glucose-induced profibrotic gene expression did not accompany it. In both HEK293 and TKPTS cells, elevated D-glucose concentrations increased the mRNA expression of *TGF-β1* and *CTGF,* consistent with the established role of hyperglycaemia in promoting profibrotic responses in renal cells. However, treatment with metformin did not significantly attenuate the glucose-induced expression of either *TGF-β1* or *CTGF* under our experimental conditions. In contrast, metformin markedly reduced glucose-induced *MORG1* mRNA expression in both cell types. These findings suggest that metformin’s effect on MORG1 is not simply a consequence of broad inhibition of the cellular response to high glucose. This distinction matters when considering the potential relationship between MORG1 and metformin’s renoprotective effects.

Nevertheless, the absence of an inhibitory effect of metformin on *TGF-β1* and *CTGF* expression in our cellular models suggests that metformin’s regulation of MORG1 may occur independently of these classical profibrotic pathways, at least under the conditions investigated in the present study. Moreover, our in vitro data suggest that reducing *MORG1* alone, particularly in response to metformin, does not necessarily result in an immediate reduction in *TGF-β1* or *CTGF* expression. This discrepancy may reflect differences between acute cellular responses and chronic tissue-level responses, as well as differences between isolated renal cells and the complex cellular environment of the kidney in vivo.

Overall, our data identified MORG1 as a glucose-responsive gene in human embryonic kidney-derived HEK293 cells and provide evidence that metformin can modulate this response. Given the previous observations that reduced MORG1 expression confers renoprotective effects in diabetic mouse models [27,28], our findings suggest a potential mechanistic link among metformin, hyperglycaemia, *MORG1* expression, and diabetic kidney injury.

In addition, the concordance between findings in endogenously *MORG1*-luciferase-tagged HEK293 cells and parental HEK293 cells demonstrates the utility of CRISPR/Cas9-mediated genome editing for integrating reporter genes. In our study, the firefly luciferase reporter enabled the monitoring of *MORG1* expression and highlights the potential of this approach for screening compounds that modulate *MORG1* gene expression. Our current findings, together with our previous studies from animal models of T1DM and T2DM, suggest that MORG1 could be a potential therapeutic target for treatment and prevention of diabetic kidney injury.

Although HEK293 cells were selected as a human embryonic kidney-derived cell model, an important limitation of the present study is that these cells do not represent kidney tubular epithelial cells. Therefore, the findings should be further validated in human kidney tubular cells. In addition, the observed increase in MORG1 expression was relatively modest, and further studies are needed to determine whether these transcriptional changes translate into biologically relevant functions. Thus, the effects of high glucose and metformin on *MORG1* expression should also be investigated in human kidney tubular cells. Further studies should explore the upstream signalling pathways mediating glucose-dependent regulation of *MORG1* mRNA and protein expression and assess the functional significance of its modulation in renal cell types [43].

### Study Limitations

The primary objective of the present study was to evaluate the functionality of the endogenously introduced *MORG1*-luciferase reporter in HEK293 cells generated by CRISPR/Cas9 genome editing. HEK293 cells provide a useful model system for investigating the regulation of the MORG1-reporter; however, they are not an appropriate cellular model for evaluating the functional consequences of *MORG1* elevated expression under diabetic conditions. Therefore, an important limitation of the present study is that HEK293 cells do not represent kidney tubular epithelial cells. Consequently, the findings should be further validated in murine or human kidney tubular cells.

Although we performed preliminary experiments in TKPTS (murine proximal tubular) cells and investigated the effects of 20 mM D-glucose and 20 µM metformin on *Morg1* mRNA expression, which showed a similar trend to that observed in HEK293 cells, the lack of in vivo validation remains an additional limitation. Therefore, our findings should be interpreted with caution.

Furthermore, our observations do not establish the direct functional consequences of major glucose-dependent changes in *MORG1* expression. In particular, the detected effect was relatively modest, and further studies are required to determine whether these transcriptional changes translate into biologically relevant effects in renal cells and tissues.

In addition, our preliminary analyses of AMPK phosphorylation in HEK293 and TKPTS cells indicate that AMPK signalling may be associated with the effects of metformin and high glucose on *MORG1* expression. However, these observations are preliminary and do not establish a direct mechanistic role for AMPK in the regulation of MORG1. Further studies are, therefore, required to validate these findings and to determine whether and how AMPK signalling is involved in regulating *MORG1* expression. Such studies should include experiments in relevant renal tubular cell models, animal models of T1DM and T2DM, and, where appropriate, human diabetic samples.

## 5. Conclusions

Overall, our study identifies MORG1 as a glucose-responsive gene in HEK293 cells and provides evidence that metformin can modulate this response. Given previous observations that reduced *Morg1* expression confers renoprotective effects in diabetic mouse models [25,26], our findings suggest a potential mechanistic link between hyperglycaemia, metformin action, *MORG1* expression, and diabetic kidney injury, warranting further investigations. Our findings support a potential role of MORG1 as a therapeutic target for the treatment and prevention of diabetes-related kidney damage.

Furthermore, the successful generation and validation of an endogenous MORG1–luciferase reporter cell line demonstrates the utility of CRISPR/Cas9-mediated reporter gene integration for studying gene regulation and screening compounds that modulate *MORG1* gene expression.

Diabetes mellitus is a multifactorial disease in which hyperglycaemia is only one of several pathogenic stimuli. Other contributors to disease progression include dysregulation of angiotensin II signalling, accumulation of advanced glycated end products (AGEs), increased production of reactive oxygen species (ROS), enhanced inflammatory signalling, epigenetic alterations, and metabolic memory. Therefore, our observation should be further evaluated in patients with diabetes, including those receiving insulin and/or metformin therapy.

## Data Availability

The original contributions presented in this study are included in the article and Supplementary Material. Further inquiries can be directed to the corresponding author.

## Supplementary Materials

The following supporting information can be downloaded at: https://www.mdpi.com/article/10.3390/cimb48090934/s1.
