# Supplementary material for: Metformin Attenuates the High-Glucose Effect on MORG1 Expression in HEK293 Cells
Source: Curr Issues Mol Biol. 2026 Sep 13;48(9):934. doi: 10.3390/cimb48090934 (PMC13604954; doi:10.3390/cimb48090934)

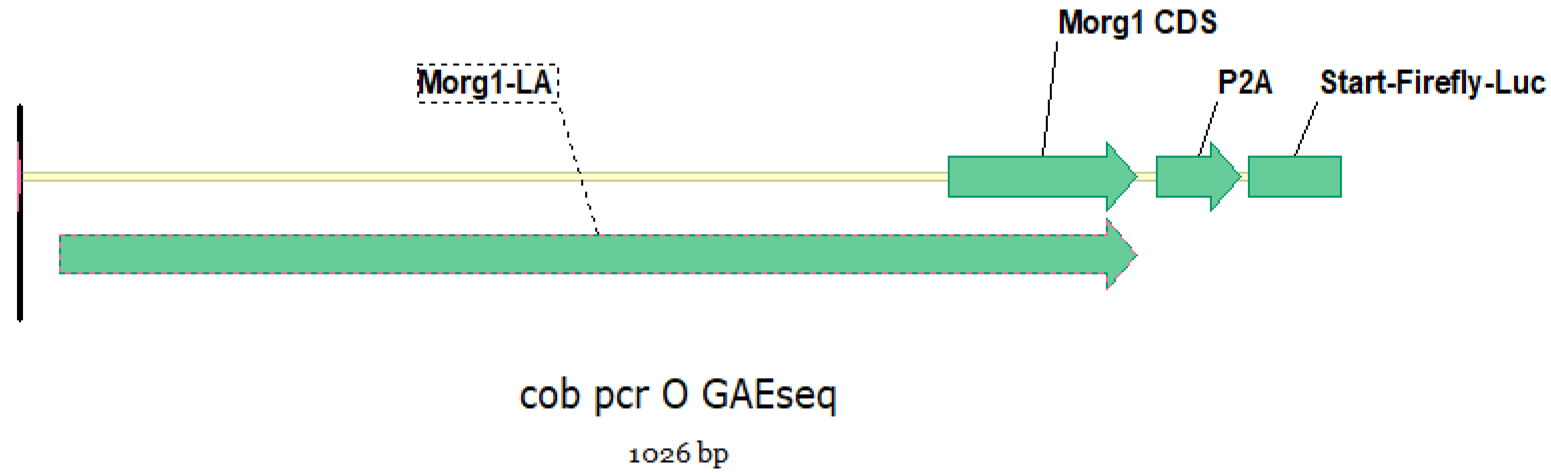


**Figure S1.** Sequencing result and annotations of the PCR-product from genomic DNA of endogenous HEK293-MORG1-firefly luciferase tagging. The graph was prepared by the use of VNTI Advance 9.1 software based on annealing of the genomic MORG1 sequence and the DNA sequences from the sequencing files. The sequencing analyses were performed with the following sequencing primers: forward primer 5′-CATGGTCTAGAAGCAGAGCCAAGAGG-3′ that binds to a sequence upstream from the integration cassette. The corresponding sequencing results are shown in Table S3.

Morg1-LA - Morg1 genomic sequence Left Arm; Morg1 CDS - Morg1 coding sequence; P2A - peptide 2A sequence, Start Firefly-Luc - start codon of the Firefly-Luciferase gene. Cob pcr O GAEseq – this is the name of the sequencing probe that was sent for sequencing to Eurofins Genomic, Ebersberg, Germany.

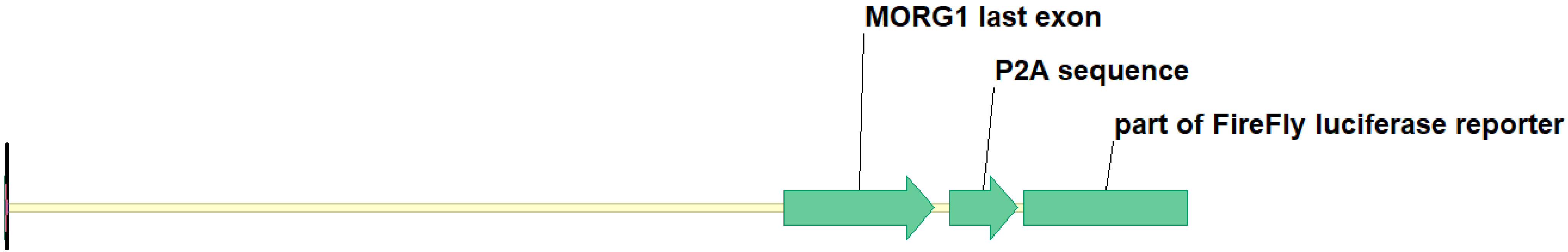


**Figure S2**. Sequencing result of a PCR product amplified from genomic DNA of endogenous HEK293-MORG1-Firefly Luciferase tagging. The PCR product was amplified and subcloned into pJET1.2 vector plasmid. The sequencing results confirmed that the coding sequence of *MORG1* last exon is tagged in frame with a P2A linking sequence followed by the Firefly luciferase reporter gene, demonstrating the correct integration of the inserted cassette into the genome locus of *MORG1* gene. Sequencing result and annotations of the PCR-product from genomic DNA of endogenous HEK293-MORG1-firefly Luciferase tagging. The graph was prepared using the VNTI Advance 9.1 software. The sequencing analyses were performed using purified plasmid DNA from pJET1.2 - PCR fragment, with pJET1.2 forward and pJET1.2 reverse primers, provided from the Eurofins Genomics, Ebersberg, Germany. The sequencing results are shown in Table S5.

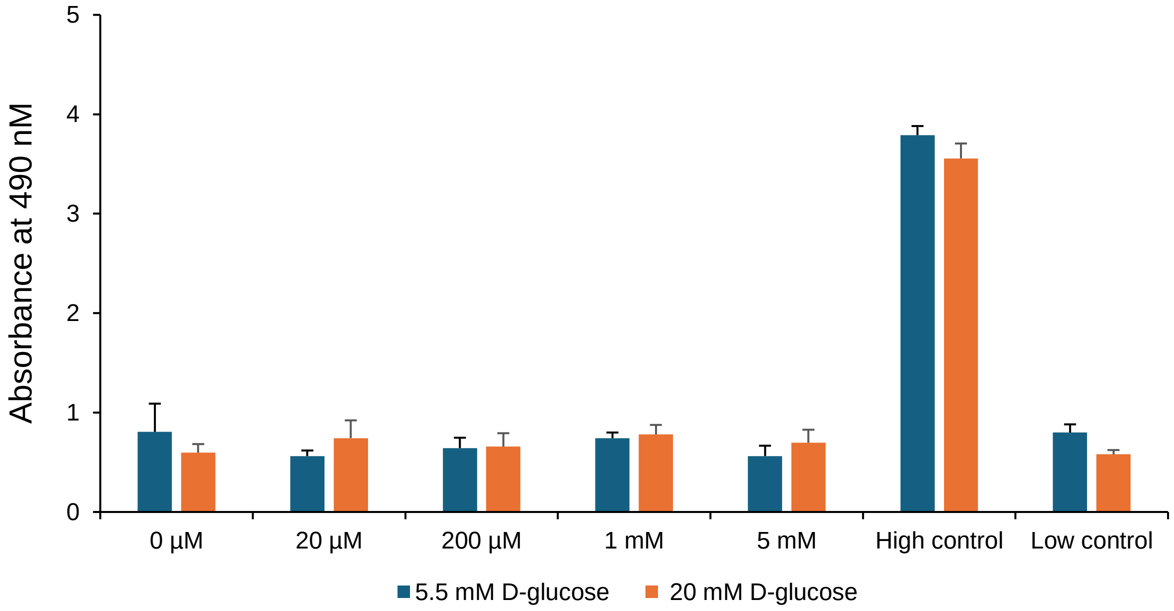


**Figure S3.** Analyses of metformin cytotoxicity in 5 mM- and 20 mM D-glucose treated HEK293 cells.The cytotoxic effect of metformin was assessed by the aid of LDH –Cytox assay in HEK293 cells. The metformin cellular toxicity was analysed at cells incubated with 5.5 mM D-glucose alone or in the presence of 20 μM, 200 μM, 1 mM or 5 mM metformin for 24 h ( ) as well as in HEK293 cells exposed to 20 mM D-glucose alone or in the presence of 20 μM, 200 μM, 1 mM or 5 mM metformin for 24 h ( ). The low control is equal to the untreated cells, whereas the high control represents cells, which were lysed before the assay and LDH was released in the supernatant and serves as a positive control for the assay. The low control and high control were performed for 5.5 mM- as well as for 20 mM D-glucose treated cells. All treatments were performed in triplicates. The absorbance of formazan, which is proportional to LDH released in the cell culture supernatant, was measured at 490 nM and the average of the assays is graphically presented.

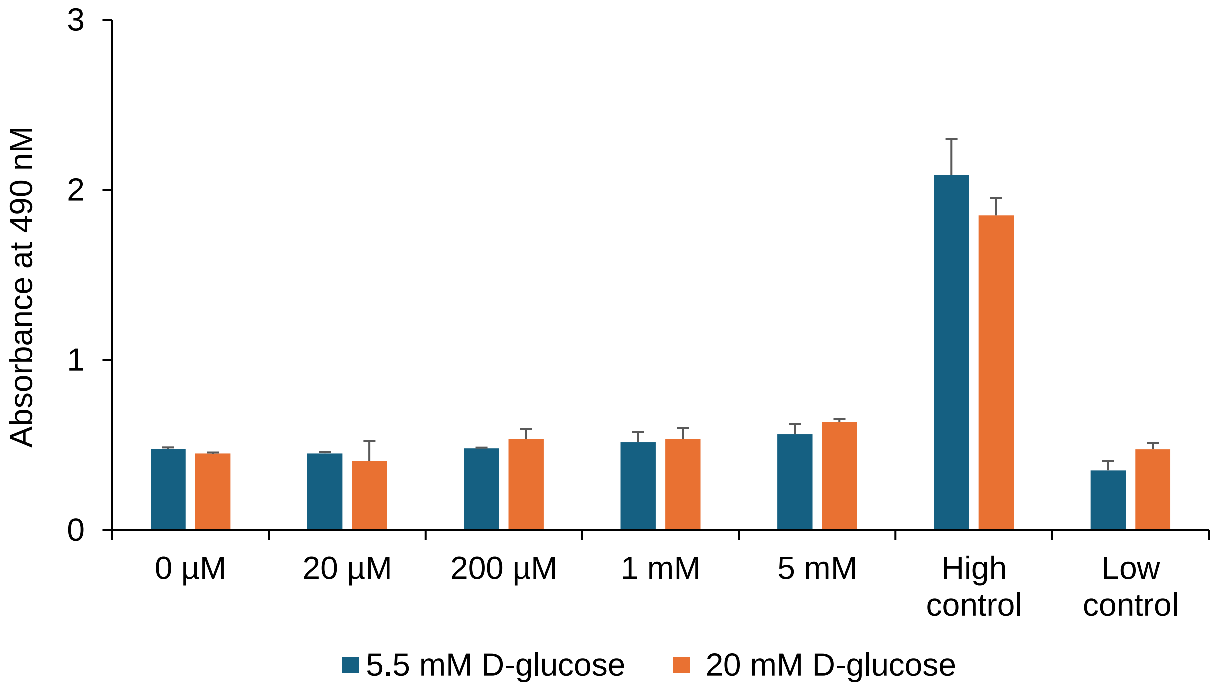


**Figure S4.** Analyses of metformin cytotoxicity in 5 mM- and 20 mM D-glucose treated TKPTS cells.The cytotoxic effect of metformin was assessed by the aid of LDH –Cytox assay in TKPTS cells. The metformin cellular toxicity was analysed at cells incubated with 5.5 mM D-glucose alone or in the presence of 20 µM, 200 µM, 1 mM or 5 mM metformin for 24 h ( ) as well as in TKPTS cells exposed to 20 mM D-glucose alone or in the presence of 20 µM, 200 µM, 1 mM or 5 mM metformin for 24 h ( ). The low control is equal to the untreated cells, whereas the high control represents cells, which were lysed before the assay and LDH was released in the supernatant and serves as a positive control for the assay. The low control and high control were performed for 5.5 mM- as well as for 20 mM D-glucose treated cells. All treatments were performed in triplicates. The absorbance of formazan, which is proportional to LDH released in the cell culture supernatant, was measured at 490 nM and the average of the assays is graphically presented.

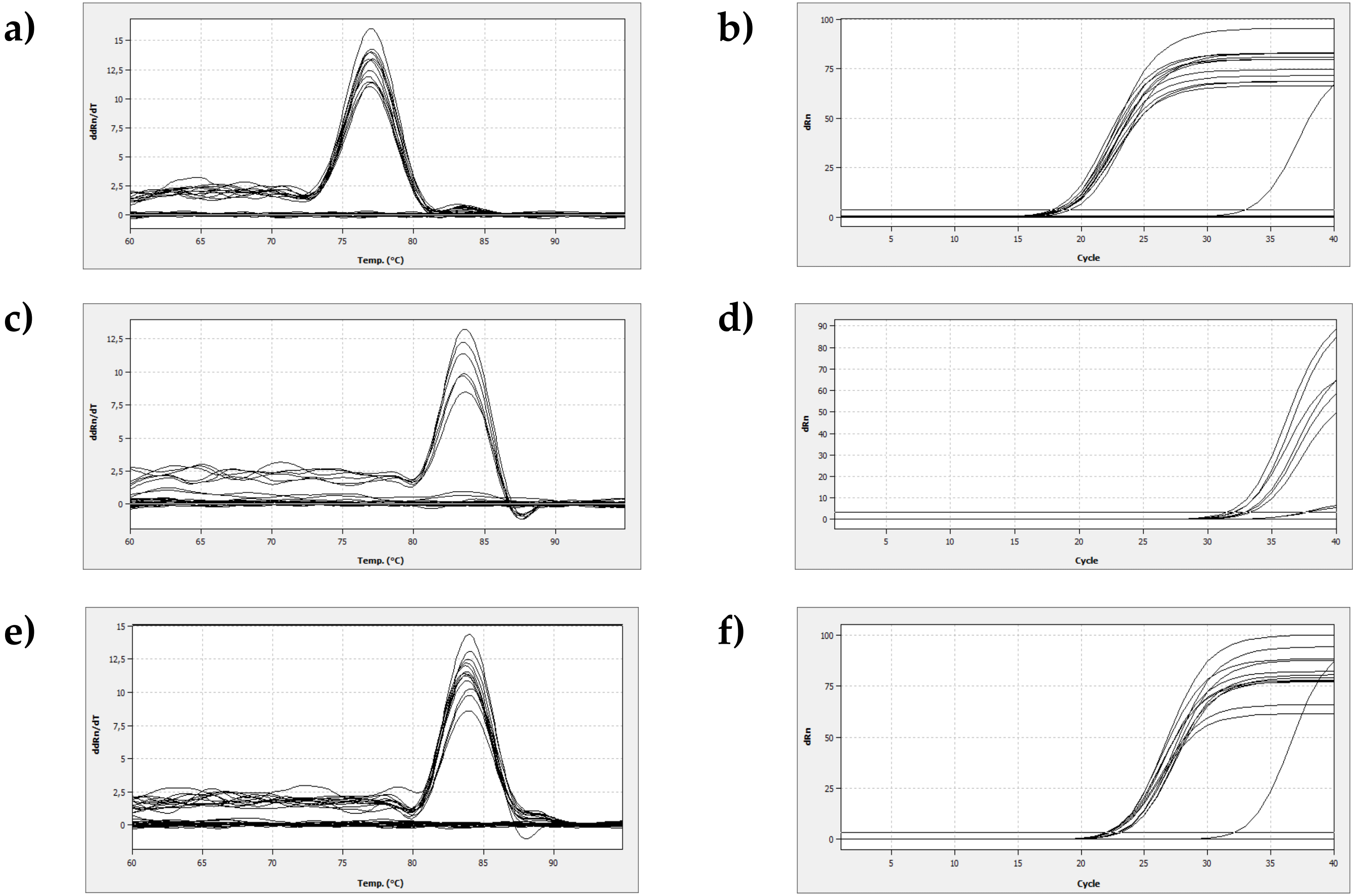


**Figure S5.** Analyses of *OCT-2, MATE-1* and *HPRT* mRNA expression in parental HEK293 cells by real time PCR. Analyses were performed from HEK293 cells treated with DMEM medium containing 5.5 mM D-glucose, 0.5 % heat inactivated FCS for 24 h. (**a**) melting curve of the house keeping gene *HPRT;* (**b**) expression profiles of *HPRT;* (**c**) melting curve of *OCT2*; (**d**) expression profiles of *OCT2* (**e**) melting curve of *MATE-1*; (**f**). expression profiles of *MATE-1.*

*HPRT-* Hypoxanthine-Guanine Phosphoribosyltransferase; *OCT-2-* organic cation transporter - 2; *MATE-1-* multidrug and toxin extrusion - 1

**Table S1**. Primer sequences used for qRT-PCR amplification analyses of human cDNAs. *HPRT1* = hypoxanthine phosphoribosyl transferase 1, *CTGF* = connective tissue growth factor, *TGF-β1* = transforming growth factor-β1, *OCT-2* = organic cation transporter-2, *MATE-1* = multidrug and toxin extrusion-1.

| **Primer** | **Sequence (5'- 3')** |
|---|---|
| *HPRT* forward | AGTGTTGGATATAAGCCAGA |
| *HPRT* reverse | CAAATCCCTGAAGTATTCATT |
| *MORG1* forward | AGGAATACAAGCTGGACTG |
| *MORG1* reverse | GCTCCATCCTCTGCCTCATAG |
| *OCT-2* forward | GAATTTGTTGGGCGGAGATAT |
| *OCT-2* reverse | AGGTATGCACCAGTAATAGAG |
| *MATE-1* forward | GTCAGCAGGCTCAGGTACAC |
| *MATE-1* reverse | CTGCTGATCTGACTGAGGCTC |
| *CTGF* forward | CTTGCGAAGCTGACCTGGAAGA |
| *CTGF* reverse | CCGTCGGTACATACTCCACAGA |
| *TGF -β1* forward | TACCTGAACCCGTGTTGCTCTC |
| *TGF -β1* reverse | GTTGCTGAGGTATCGCCAGGAA |

**Table S2**. Primer sequences used for qRT-PCR analyses of murine cDNAs.

*Hprt1* = hypoxanthine phosphoribosyl transferase 1, *Ctgf* = connective tissue growth factor, *Tgf-β1* = transforming growth factor-β1

| **Primer** | **Sequence (5'- 3')** |
|---|---|
| *Hprt* forward | TGCTCGAGATGTCATGAAGG |
| *Hprt* reverse | TATGTCCCCCGTTGACTGAT |
| *Morg1* forward | CCTATCACCTGCACCTGCTT |
| *Morg1* reverse | CACTTTCCCGTCTTCAGAGC |
| *Ctgf* forward | CCTGGTCCAGACCACAGAGT |
| *Ctgf* reverse | TTTTCCTCCAGGTCAGCTTC |
| *Tgf β*-1 forward | AAGGGCTACCATGCCAACTT |
| *Tgf β*-1 reverse | CGGGTTGTGTTGGTTGTAGA |

Sequence: SEQ10023617_A2_G11_0

Samples: 17007
Bases: 1064
Average spacing: 16.0
Average quality >= 10: 19, 20: 42, 30: 991

Quality: 0 - 9, 10 - 19, 20 - 29, >= 30


02.11.2023

Table S3. Sequencing results of the MORG1–firefly luciferase tagging PCR product amplified from HEK293 cells. Sequencing analyses using the forward sequencing primer.

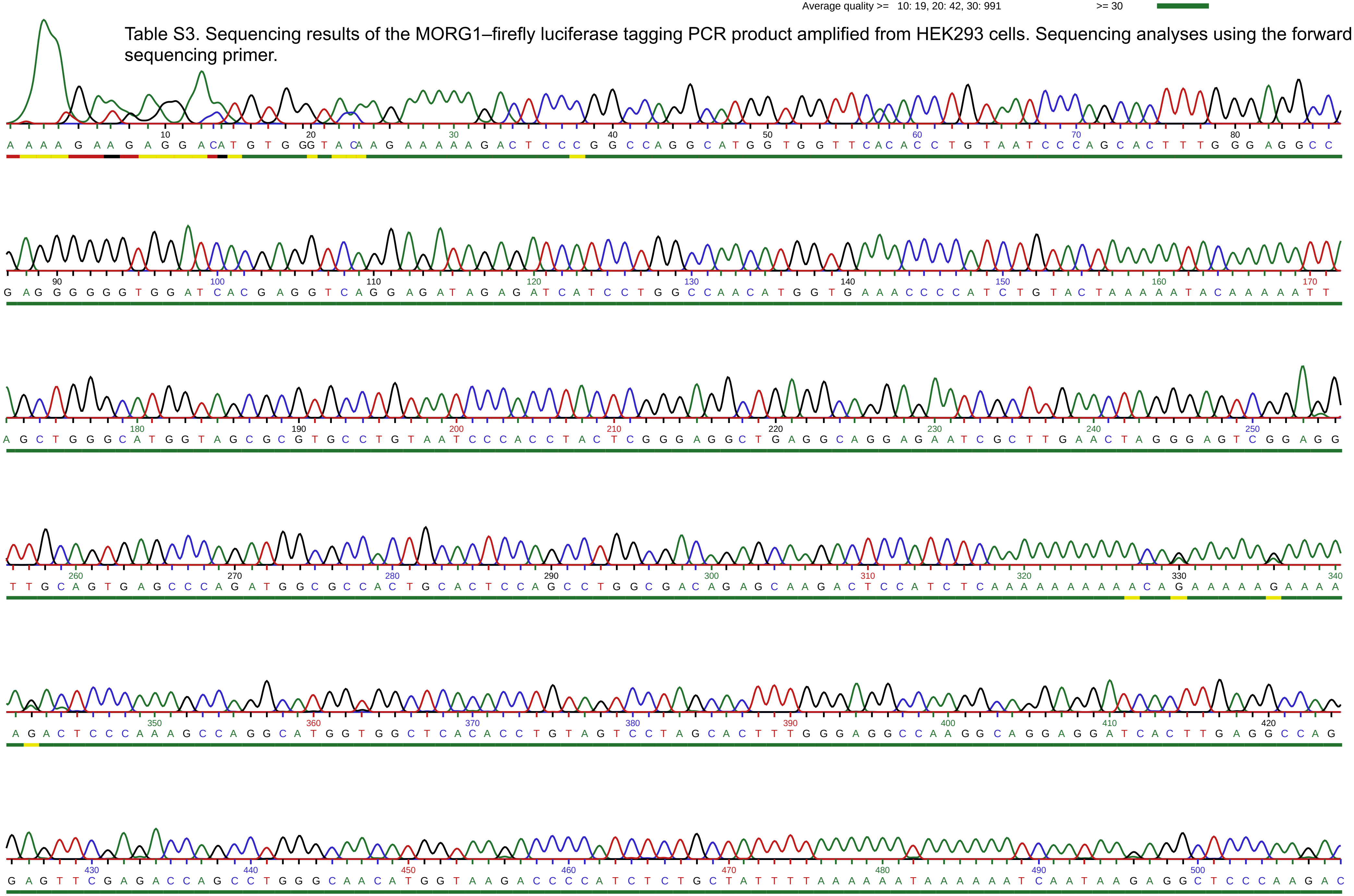

Sequence: SEQ10023617_A2_G11_0

| | | | | |
|---|---|---|---|---|
| Samples: | 17007 | Quality: | 0 - 9 | |
| Bases: | 1064 | | 10 - 19 | |
| Average spacing: | 16.0 | | 20 - 29 | |
| Average quality >= | 10: 19, 20: 42, 30: 991 | | >= 30 | |



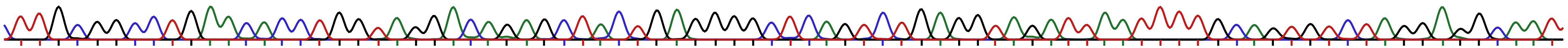

510 520 530 540 550 560 570 580 590

T T G C G G C C T G A A C A C C T G G T A G G A C A G A G C T A C T G A G G G G C T C A G T C T G A G G T A G A T T A A T T T T G C A G T G T C T A G G A G G C A A T

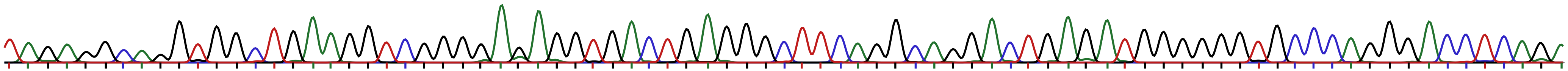

600 610 620 630 640 650 660 670

T A G A G G C A G G T G G C T G A A G G T C G G G G A G A G G T G A C T G A G G G C T T C A G G C A G G A C T G A G A T G G G G G G T G C C C A G G G A C C T C A G A

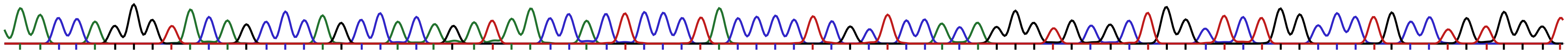

680 690 700 710 720 730 740 750

A A C C A G G G T A C A G C C C A G C C A C A G A T A A C C A C T C C C T A C C C C T C G C T C C A C A G G G T G C G C T G G C T C T G G C C C T G C C T G T G G G T

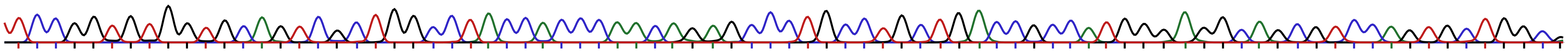

760 770 780 790 800 810 820 830 840

T C C G G T G T G G T G C A G T C G C T G G C C T A C C A C C C A A C A G A G C C C T G C C T G A C C G C C A T G G G A G G C A G C G T C C A G T G C T G G C G

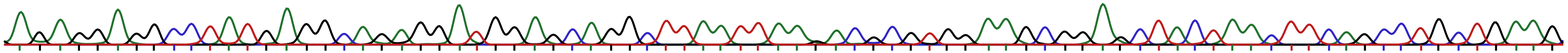

850 860 870 880 890 900 910 920

A G A G G A G G C C T A T G A G G C A G A G G A T G G A G C A G G C T T A A T T A A G A C G C G T G G A A G C G G A G C T A C T A A C T T C A G C C T G C T G A A G

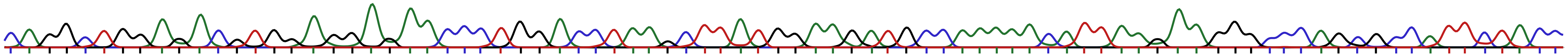

930 940 950 960 970 980 990 1000

C A G G C T G G A G A C G T G G A G G A G A A C C C T G G A C C T A A G C T T A T G G A A G A T G C C A A A A A C A T T A A G A A G G G C C C A G C G C C A T T C T A C C

Sequence: SEQ10023617_A2_G11_0

| | | | | |
|---|---|---|---|---|
| Samples: | 17007 | Quality: | 0 - 9 | |
| Bases: | 1064 | | 10 - 19 | |
| Average spacing: | 16.0 | | 20 - 29 | |
| Average quality >= | 10: 19, 20: 42, 30: 991 | | >= 30 | |


02.11.2023

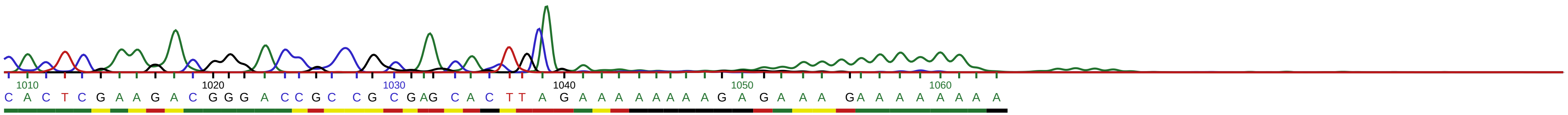

Sequence: SEQ10023617_A2_H11_0

| | | | | | |
|---|---|---|---|---|---|
| Samples: | 16487 | Quality: | 0 - 9 | black | Page: 1 / 3 |
| Bases: | 1084 | | 10 - 19 | red | 02.11.2023 |
| Average spacing: | 16.0 | | 20 - 29 | yellow | |
| Average quality >= | 10: 37, 20: 16, 30: 1001 | | >= 30 | green | |

Table S4. Sequencing results of the MORG1–firefly luciferase tagging PCR product amplified from HEK293 cells. Sequencing analyses using the reverse primer.

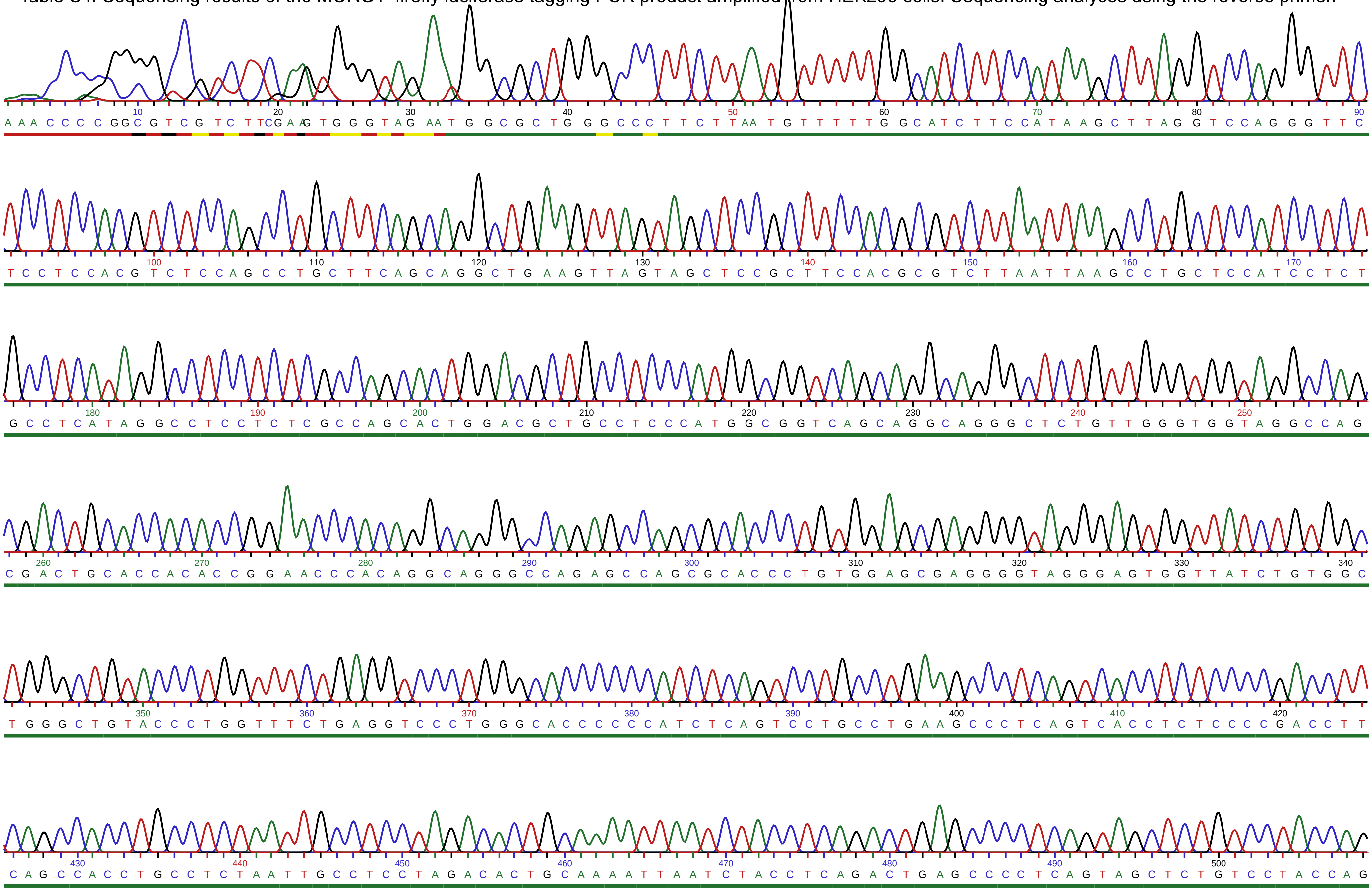

Sequence: SEQ10023617_A2_H11_0

| | | | | | |
|---|---|---|---|---|---|
| Samples: | 16487 | Quality: | 0 - 9 | | Page: 2 / 3 |
| Bases: | 1084 | | 10 - 19 | | 02.11.2023 |
| Average spacing: | 16.0 | | 20 - 29 | | |
| Average quality >= | 10: 37, 20: 16, 30: 1001 | | >= 30 | | |

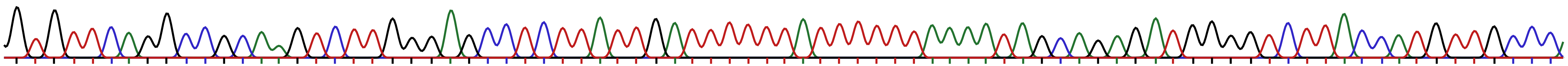

510 520 530 540 550 560 570 580 590

G T G T T C A G G C C G C A A G T C T T G G G A G C C T C T T A T T G A T T T T T T A T T T T T T A A A A T A G C A G A G A T G G G G T C T T A C C A T G T T G C C C

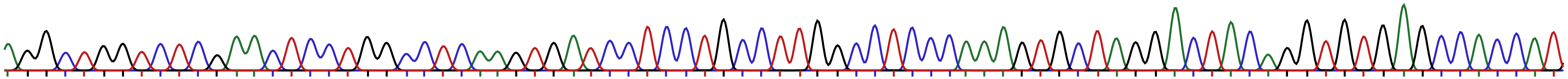

600 610 620 630 640 650 660 670

A G G C T G G T C T C G A A C T C C T G G C C T C A A G T G A T C C T C C T G C C T T G G C C T C C C A A A G T G C T A G G A C T A C A G G T G T G A G C C A C C A T

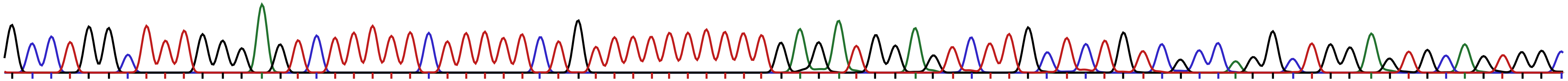

680 690 700 710 720 730 740 750

G C C T G G C T T T G G G A G T C T T T T T C T T T T T C T G T T T T T T T T T G A G A T G G A G T C T T G C T C T G T C G C C A G G C T G G A G T G C A G T G G C

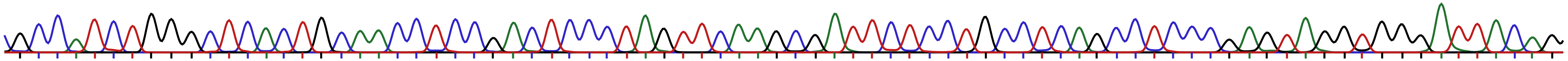

760 770 780 790 800 810 820 830 840

G C C A T C T G G G C T C A C T G C A A C C T C C G A C T C C C T A G T T C A A G C G A T T C T C C T G C C T C A G C C T C C C G A G T A G G T G G G A T T A C A G

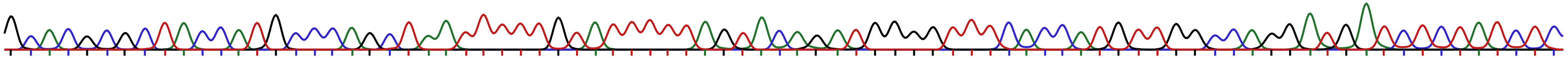

850 860 870 880 890 900 910 920

G C A C G C G C T A C C A T G C C C A G C T A A T T T T T G T A T T T T T A G T A C A G A T G G G G T T T C A C C A T G T T G G C C A G G A T G A T C T C T A T C T C

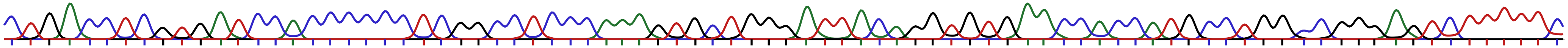

930 940 950 960 970 980 990 1000

C T G A C C T C G T G A T C C A C C C C C T C G G C C T C C C A A A G T G C T G G G A T T A C A G G T G T G A A C C A C C A T G C C T G G C C G G G A G T C T T T T T C

Sequence: SEQ10023617_A2_H11_0

| | | | | | |
|---|---|---|---|---|---|
| Samples: | 16487 | Quality: | 0 - 9 | (black) | Page: 3 / 3 |
| Bases: | 1084 | | 10 - 19 | (red) | 02.11.2023 |
| Average spacing: | 16.0 | | 20 - 29 | (yellow) | |
| Average quality >= | 10: 37, 20: 16, 30: 1001 | | >= 30 | (green) | |

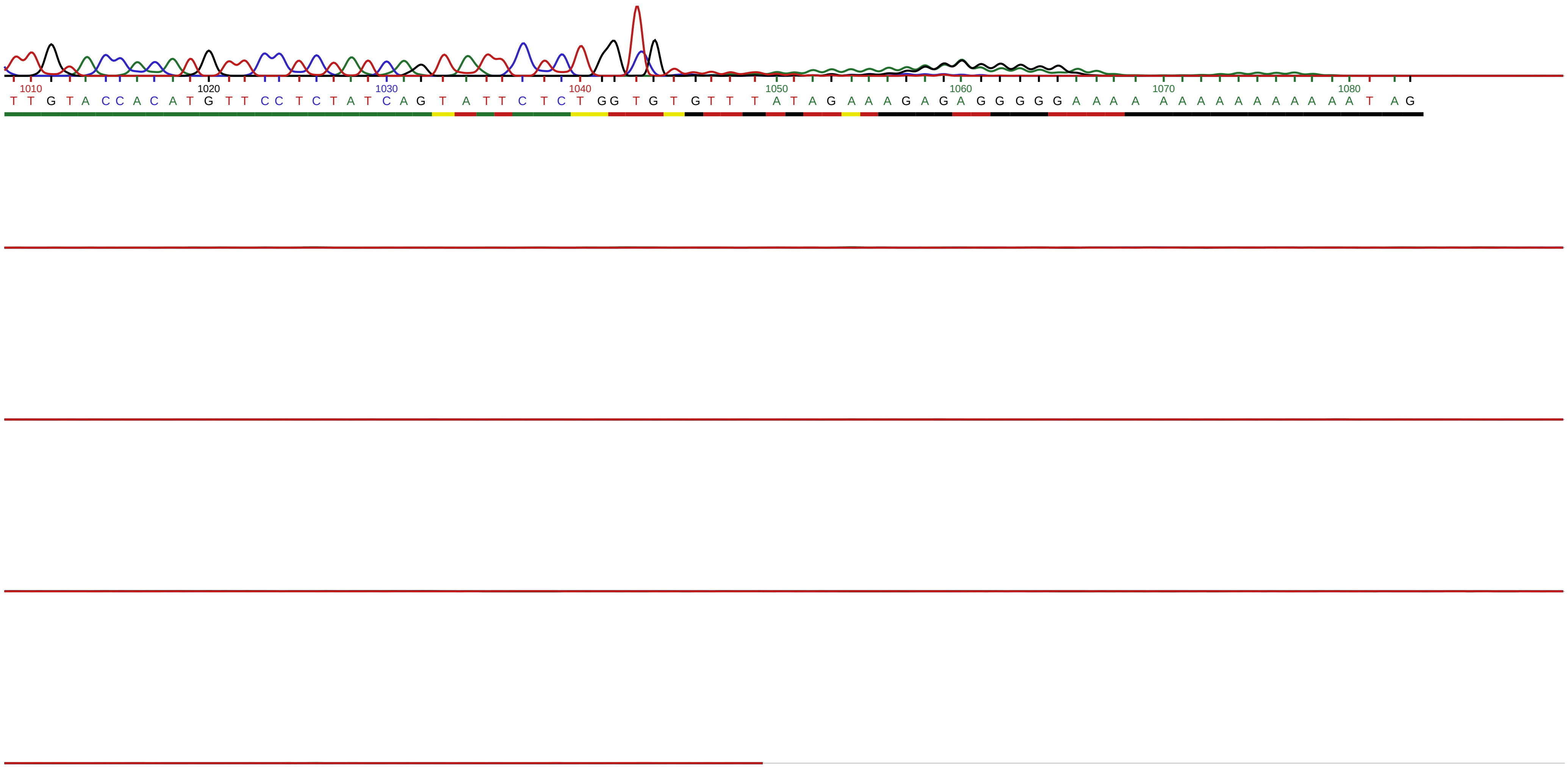

Table S5. Sequencing results of the MORG1–firefly luciferase tagging PCR product amplified from HEK293 cells and cloned into the pJET1.2 vector. Sequencing file 11108453951 was generated using the Forward pJET1.2 F primer provided by the company. The obtained sequence was aligned with the genomic MORG1 locus to verify the expected sequence.

```
11108453951.fasta
            10        20        30        40        50        60        70        80        90       100
        *    *    *    *    *    *    *    *    *    *    *    *    *    *    *    *    *    *    *    *

                                                   48 RsaI
                    17 TaqI                  42 NlaIII
                   16 XhoI                  41 PciI                   67 HaeIII     81 Hpy8I
                   16 AvaI                  41 NspI                 65 HpaII 74 NlaIII
                   ||                       ||     |                | |      |      |
  1 CCCGATTTCGGAAGGCTCGAGTTTTTCAGCAAGATGAGGAACATGTGGTACAAGAAAAAGACTCCCGGCCAGGCATGGTGGTTCACACCTGTAATCCCAG 100
    P  D  F  G  R  L  E  F  F  S  K  M  R  N  M  W  Y  K  K  K  T  P  G  Q  A  W  W  F  T  P  V  I  P  A
     P  I  S  E  G  S  S  F  S  A  R  *  G  T  C  G  T  R  K  R  L  P  A  R  H  G  G  S  H  L  *  S  Q
      R  F  R  K  A  R  V  F  Q  Q  D  E  E  H  V  V  Q  E  K  D  S  R  P  G  M  V  V  H  T  C  N  P  S
    GGGCTAAAGCCTTCCGAGCTCAAAAAGTCGTTCTACTCCTTGTACACCATGTTCTTTTTCTGAGGGCCGGTCCGTACCACCAAGTGTGGACATTAGGGTC


           110       120       130       140       150       160       170       180       190       200
        *    *    *    *    *    *    *    *    *    *    *    *    *    *    *    *    *    *    *    *

                               128 BssSI                         162 NlaIII        180 RsaI            200 AluI
              111 HaeIII    125 DpnI              147 DpnI 156 HaeIII             179 TatI         196 MluCI
              |             |  |                  |        |     |                ||               |   |
101 CACTTTGGGAGGCCGAGGGGGGTGGATCACGAGGTCAGGAGATAGAGATCATCCTGGCCAACATGGTGAAACCCCATCTGTACTAAAAATACAAAAATTA 200
      L  W  E  A  E  G  G  G  S  R  G  Q  E  I  E  I  I  L  A  N  M  V  K  P  H  L  Y  *  K  Y  K  N  *
    H  F  G  R  P  R  G  V  D  H  E  V  R  R  *  R  S  S  W  P  T  W  *  N  P  I  C  T  K  N  T  K  I  S
     T  L  G  G  R  G  G  W  I  T  R  S  G  D  R  D  H  P  G  Q  H  G  E  T  P  S  V  L  K  I  Q  K  L
    GTGAAACCCTCCGGCTCCCCCCACCTAGTGCTCCAGTCCTCTATCTCTAGTAGGACCGGTTGTACCACTTTGGGGTAGACATGATTTTTATGTTTTTAAT


           210       220       230       240       250       260       270       280       290       300
        *    *    *    *    *    *    *    *    *    *    *    *    *    *    *    *    *    *    *    *
                   216 Cac8I
                  215 BstUI
                 214 HhaI
          207 NlaIII                    237 AvaI                        269 BfaI        285 HpyCH4V
          |      |||                    |                               |               |
201 GCTGGGCATGGTAGCGCGTGCCTGTAATCCCACCTACTCGGGAGGCTGAGGCAGGAGAATCGCTTGAACTAGGGAGTCGGAGGTTGCAGTGAGCCCAGAT 300
     L  G  M  V  A  R  A  C  N  P  T  Y  S  G  G  *  G  R  R  I  A  *  T  R  E  S  E  V  A  V  S  P  D
      W  A  W  *  R  V  P  V  I  P  P  T  R  E  A  E  A  G  E  S  L  E  L  G  S  R  R  L  Q  *  A  Q  M
    A  G  H  G  S  A  C  L  *  S  H  L  L  G  R  L  R  Q  E  N  R  L  N  *  G  V  G  G  C  S  E  P  R  W
    CGACCCGTACCATCGCGCACGGACATTAGGGTGGATGAGCCCTCCGACTCCGTCCTCTTAGCGAACTTGATCCCTCAGCCTCCAACGTCACTCGGGTCTA


           310       320       330       340       350       360       370       380       390       400
        *    *    *    *    *    *    *    *    *    *    *    *    *    *    *    *    *    *    *    *
            309 HpyCH4V
     302 HhaI
    301 SfoI
    301 NarI
    301 KasI
    301 HaeII                                                                            386 NlaIII
    ||      |                                                                            |
301 GGCGCCACTGCACTCCAGCCTGGCGACAGAGCAAGACTCCATCTCAAAAAAAAAACAGAAAAAGAAAAAGACTCCCAAAGCCAGGCATGGTGGCTCACAC 400
    G  A  T  A  L  Q  P  G  D  R  A  R  L  H  L  K  K  K  T  E  K  E  K  D  S  Q  S  Q  A  W  W  L  T  P
     A  P  L  H  S  S  L  A  T  E  Q  D  S  I  S  K  K  K  Q  K  K  K  K  K  T  P  K  A  R  H  G  G  S  H
      R  H  C  T  P  A  W  R  Q  S  K  T  P  S  Q  K  K  N  R  K  R  K  R  L  P  K  P  G  M  V  A  H  T
    CCGCGGTGACGTGAGGTCGGACCGCTGTCTCGTTCTGAGGTAGAGTTTTTTTTTTGTCTTTTTCTTTTTCTGAGGGTTTCGGTCCGTACCACCGAGTGTG


           410       420       430       440       450       460       470       480       490       500
        *    *    *    *    *    *    *    *    *    *    *    *    *    *    *    *    *    *    *    *
                                                            457 TaqI
            409 BfaI      423 HaeIII    437 DpnI  447 HaeIII  459 BsaI         476 NlaIII
            |             |             |         |         | |                |
401 CTGTAGTCCTAGCACTTTGGGAGGCCAAGGCAGGAGGATCACTTGAGGCCAGGAGTTCGAGACCAGCCTGGGCAACATGGTAAGACCCCATCTCTGCTAT 500
      V  V  L  A  L  W  E  A  K  A  G  G  S  L  E  A  R  S  S  R  P  A  W  A  T  W  *  D  P  I  S  A  I
    L  *  S  *  H  F  G  R  P  R  Q  E  D  H  L  R  P  G  V  R  D  Q  P  G  Q  H  G  K  T  P  S  L  L  F
     C  S  P  S  T  L  G  G  Q  G  R  R  I  T  *  G  Q  E  F  E  T  S  L  G  N  M  V  R  P  H  L  C  Y
    GACATCAGGATCGTGAAACCCTCCGGTTCCGTCCTCCTAGTGAACTCCGGTCCTCAAGCTCTGGTCGGACCCGTTGTACCATTCTGGGGTAGAGACGATA


           510       520       530       540       550       560       570       580       590       600
        *    *    *    *    *    *    *    *    *    *    *    *    *    *    *    *    *    *    *    *
                                                                                                   596 MluCI
     502 MseI                                542 HaeIII                                          594 MseI
    501 DraI                               540 AciI                 565 AluI                    593 AseI
    ||                                     | |                      |                           || |
501 TTTAAAAAATAAAAAATCAATAAGAGGCTCCCAAGACTTGCGGCCTGAACACCTGGTAGGACAGAGCTACTGAGGGGCTCAGTCTGAGGTAGATTAATTT 600
     L  K  N  K  K  S  I  R  G  S  Q  D  L  R  P  E  H  L  V  G  Q  S  Y  *  G  A  Q  S  E  V  D  *  F
      *  K  I  K  N  Q  *  E  A  P  K  T  C  G  L  N  T  W  *  D  R  A  T  E  G  L  S  L  R  *  I  N  F
    F  K  K  *  K  I  N  K  R  L  P  R  L  A  A  *  T  P  G  R  T  E  L  L  R  G  S  V  *  G  R  L  I  L
    AAATTTTTTATTTTTTAGTTATTCTCCGAGGGTTCTGAACGCCGGACTTGTGGACCATCCTGTCTCGATGACTCCCCGAGTCAGACTCCATCTAATTAAA


           610       620       630       640       650       660       670       680       690       700
        *    *    *    *    *    *    *    *    *    *    *    *    *    *    *    *    *    *    *    *
            609 BfaI         626 PaqCI
    601 HpyCH4V      618 MluCI
    |       |        |       |
601 TGCAGTGTCTAGGAGGCAATTAGAGGCAGGTGGCTGAAGGTCGGGGAGAGGTGACTGAGGGCTTCAGGCAGGACTGAGATGGGGGGTGCCCAGGGACCTC 700
    C  S  V  *  E  A  I  R  G  R  W  L  K  V  G  E  R  *  L  R  A  S  G  R  T  E  M  G  G  A  Q  G  P  Q
     A  V  S  R  R  Q  L  E  A  G  G  *  R  S  G  R  G  D  *  G  L  Q  A  G  L  R  W  G  V  P  R  D  L
```

```
       Q  C  L  G  G  N  *  R  Q  V  A  E  G  R  G  E  V  T  E  G  F  R  Q  D  *  D  G  G  C  P  G  T  S
     ACGTCACAGATCCTCCGTTAATCTCCGTCCACCGACTTCCAGCCCCTCTCCACTGACTCCCGAAGTCCGTCCTGACTCTACCCCCCACGGGTCCCTGGAG


             710       720       730       740       750       760       770       780       790       800
         *    *    *    *    *    *    *    *    *    *    *    *    *    *    *    *    *    *    *    *
                                                                  762 Cac8I                 788 HpaII
               711 RsaI                                         760 HhaI   771 HaeIII      787 BsaWI 797 HpyCH4V
               |                                                | |        |               ||        |
 701 AGAAACCAGGGTACAGCCCAGCCACAGATAACCACTCCCTACCCCTCGCTCCACAGGGTGCGCTGGCTCTGGCCCTGCCTGTGGGTTCCGGTGTGGTGCA 800
       K  P  G  Y  S  P  A  T  D  N  H  S  L  P  L  A  P  Q  G  A  L  A  L  A  L  P  V  G  S  G  V  V  Q
     R  N  Q  G  T  A  Q  P  Q  I  T  T  P  Y  P  S  L  H  R  V  R  W  L  W  P  C  L  W  V  P  V  W  C  S
      E  T  R  V  Q  P  S  H  R  *  P  L  P  T  P  R  S  T  G  C  A  G  S  G  P  A  C  G  F  R  C  G  A
     TCTTTGGTCCCATGTCGGGTCGGTGTCTATTGGTGAGGGATGGGGAGCGAGGTGTCCCACGCGACCGAGACCGGGACGGACACCCAAGGCCACACCACGT


             810       820       830       840       850       860       870       880       890       900
         *    *    *    *    *    *    *    *    *    *    *    *    *    *    *    *    *    *    *    *
                                               843 NlaIII
           807 HaeIII                      839 AciI                             876 HaeIII
        804 Cac8I                 830 Cac8I   842 NcoI             863 Cac8I   875 StuI               898 Cac8I
        |  |                      |        |  ||                   |           ||                     |
 801 GTCGCTGGCCTACCACCCAACAGAGCCCTGCCTGCTGACCGCCATGGGAGGCAGCGTCCAGTGCTGGCGAGAGGAGGCCTATGAGGCAGAGGATGGAGCA 900
      S  L  A  Y  H  P  T  E  P  C  L  L  T  A  M  G  G  S  V  Q  C  W  R  E  E  A  Y  E  A  E  D  G  A
       R  W  P  T  T  Q  Q  S  P  A  C  *  P  P  W  E  A  A  S  S  A  G  E  R  R  P  M  R  Q  R  M  E  Q
     V  A  G  L  P  P  N  R  A  L  P  A  D  R  H  G  R  Q  R  P  V  L  A  R  G  G  L  *  G  R  G  W  S  R
     CAGCGACCGGATGGTGGGTTGTCTCGGGACGGACGACTGGCGGTACCCTCCGTCGCAGGTCACGACCGCTCTCCTCCGGATACTCCGTCTCCTACCTCGT


             910       920       930       940       950       960       970       980       990      1000
         *    *    *    *    *    *    *    *    *    *    *    *    *    *    *    *    *    *    *    *
            908 MseI
          906 MluCI                                               962 HpyCH4IV
        904 PacI  914 BstUI    927 AluI                        959 BsmBI                  986 AluI
        904 MseI 913 MluI  923 AciI          941 Cac8I 951 Cac8I 961 BmgBI               985 HindIII
        | | |    ||         |   |             |         |       | ||                      ||
 901 GGCTTAATTAAGACGCGTGGAAGCGGAGCTACTAACTTCAGCCTGCTGAAGCAGGCTGGAGACGTGGAGGAGAACCCTGGACCTAAGCTTATGGAAGATG 1000
     G  L  I  K  T  R  G  S  G  A  T  N  F  S  L  L  K  Q  A  G  D  V  E  E  N  P  G  P  K  L  M  E  D  A
      A  *  L  R  R  V  E  A  E  L  L  T  S  A  C  *  S  R  L  E  T  W  R  R  T  L  D  L  S  L  W  K  M
       L  N  *  D  A  W  K  R  S  Y  *  L  Q  P  A  E  A  G  W  R  R  G  G  E  P  W  T  *  A  Y  G  R  C
     CCGAATTAATTCTGCGCACCTTCGCCTCGATGATTGAAGTCGGACGACTTCGTCCGACCTCTGCACCTCCTCTTGGGACCTGGATTCGAATACCTTCTAC


            1010      1020      1030      1040      1050      1060      1070      1080
         *    *    *    *    *    *    *    *    *    *    *    *    *    *    *    *
                                                                1060 Cac8I
                                                             1057 HpaII
                                                            1056 NgoMIV
                                                            1056 NaeI
                              1026 HhaI                     1056 Cac8I
                        1020 HaeIII                        1055 SgrAI    1069 HpyCH4V
                       1019 ApaI                           1055 MreI  1066 AluI
               1011 MseI     1025 HaeII       1042 TaqI   1054 AciI  1065 PvuII
               |       ||    ||               |           ||||  |    ||  |
1001 CCAAAAAACATTAAGAAAGGGCCCAGCGCCATTCTACCCACTCGAAAACGGGACCGCCGGCGAGCAGCTGCACAAAGCCT 1080
       K  K  H  *  E  R  A  Q  R  H  S  T  H  S  K  T  G  P  P  A  S  S  C  T  K  P
     P  K  N  I  K  K  G  P  S  A  I  L  P  T  R  K  R  D  R  R  R  A  A  A  Q  S
      Q  K  T  L  R  K  G  P  A  P  F  Y  P  L  E  N  G  T  A  G  E  Q  L  H  K  A
     GGTTTTTTGTAATTCTTTCCCGGGTCGCGGTAAGATGGGTGAGCTTTTGCCCTGGCGGCCGCTCGTCGACGTGTTTCGGA
```

**Table S6**. Schreier-Ray-Hare Analyses of the time dependent effect of 20 mM D-glucose on luciferase activity in *MORG1*-luciferase tagged HEK293 cells. The analyses were performed using a Scheirer-Ray-Hare Analysis as a non-parametric alternative to a two-way ANOVA.

# Scheirer-Ray-Hare Analysis

RM

2026-08-31

## Table of Contents


Setup ........ 1
Install and load required packages ........ 1
Import data ........ 2
Check column names ........ 2
Clean and prepare the data ........ 3
Data structure ........ 4
Number of samples per experimental group ........ 4
Scheirer-Ray-Hare test ........ 5
Interpretation of the results ........ 5
Boxplot: Measurements by glucose concentration ........ 6
Boxplot: Measurements by time ........ 7
Boxplot: Glucose concentration and time ........ 8
Alternative combined boxplot ........ 9
Summary statistics ........ 10
Save results ........ 11
Session information ........ 11


## Setup

## Install and load required packages

Install the packages below if they are not already installed.

```r
required_packages <- c(
  "readxl",
  "rcompanion",
  "ggplot2",
  "dplyr"
)

installed <- required_packages %in% rownames(installed.packages())

if (any(!installed)) {
  install.packages(required_packages[!installed])
}

library(readxl)
library(rcompanion)
library(ggplot2)
library(dplyr)
```

# Import data

Change "your_data.xlsx" to the name or full path of your Excel file.

```r
data <- read_excel("Time dependent MORG1- Luciferase assays (1).xlsx")
```

```r
head(data)
```

```
## # A tibble: 6 × 4
##   `Sample #` `Glucose concentration (mM)` `Time  (h)` Measurements
##        <dbl>                        <dbl>       <dbl>        <dbl>
## 1          1                          5.5           3        1.16
## 2          2                          5.5           3        1.03
## 3          3                          5.5           3        0.996
## 4          4                          5.5           3        0.945
## 5          5                          5.5           3        0.924
## 6          6                          5.5           3        0.941
```

## Check column names

```r
names(data)
```

```
## [1] "Sample #"                    "Glucose concentration (mM)"
## [3] "Time  (h)"                   "Measurements"
```

The expected Excel structure is:

| Samples # | D-Glucose concentration (mM) | Time (h) | Measurements |
|---|---|---|---|
| 1 | 5.5 mM | 3 h | 0.89378516 |
| 2 | 5.5 mM | 3 h | 1.07773315 |
| 3 | 5.5 mM | 3 h | 0.97806347 |

# Clean and prepare the data

This section renames the columns and removes units such as `mM` and `h`.

```r
# Rename columns
names(data) <- c(
  "Sample",
  "Glucose",
  "Time",
  "Measurement"
)

# Extract numeric glucose concentration
data$Glucose_numeric <- as.numeric(
  gsub("[^0-9.]", "", data$Glucose)
)

# Extract numeric time
data$Time_numeric <- as.numeric(
  gsub("[^0-9.]", "", data$Time)
)

# Convert independent variables to factors
data$Glucose <- factor(data$Glucose_numeric)

data$Time <- factor(
  data$Time_numeric,
  levels = sort(unique(data$Time_numeric))
)

# Ensure measurement is numeric
data$Measurement <- as.numeric(data$Measurement)

# Remove rows containing missing values
data <- data %>%
  filter(
    !is.na(Glucose),
    !is.na(Time),
    !is.na(Measurement)
  )

# Inspect cleaned data
head(data)
```

```
## # A tibble: 6 × 6
##   Sample Glucose Time  Measurement Glucose_numeric Time_numeric
##    <dbl> <fct>   <fct>       <dbl>           <dbl>        <dbl>
## 1      1 5.5     3           1.16              5.5            3
## 2      2 5.5     3           1.03              5.5            3
## 3      3 5.5     3           0.996             5.5            3
```

```
## 4       4 5.5     3           0.945              5.5            3
## 5       5 5.5     3           0.924              5.5            3
## 6       6 5.5     3           0.941              5.5            3
```

# Data structure

```
str(data)

## tibble [218 × 6] (S3: tbl_df/tbl/data.frame)
##  $ Sample         : num [1:218] 1 2 3 4 5 6 7 8 9 10 ...
##  $ Glucose        : Factor w/ 2 levels "5.5","20": 1 1 1 1 1 1 1 1 1 1 ...
##  $ Time           : Factor w/ 4 levels "3","6","9","12": 1 1 1 1 1 1 1 1 1
1 ...
##  $ Measurement    : num [1:218] 1.164 1.029 0.996 0.945 0.924 ...
##  $ Glucose_numeric: num [1:218] 5.5 5.5 5.5 5.5 5.5 5.5 5.5 5.5 5.5 5.5 ..
.
##  $ Time_numeric   : num [1:218] 3 3 3 3 3 3 3 3 3 3 ...

summary(data)

##      Sample      Glucose    Time     Measurement     Glucose_numeric
##  Min.   : 1.00   5.5:109   3 :66   Min.   :0.7534   Min.   : 5.50
##  1st Qu.: 7.00   20 :109   6 :54   1st Qu.:0.9972   1st Qu.: 5.50
##  Median :14.00             9 :66   Median :1.1486   Median :12.75
##  Mean   :14.28             12:32   Mean   :1.2155   Mean   :12.75
##  3rd Qu.:21.00                     3rd Qu.:1.4000   3rd Qu.:20.00
##  Max.   :30.00                     Max.   :2.0413   Max.   :20.00
##   Time_numeric
##  Min.   : 3.000
##  1st Qu.: 3.000
##  Median : 6.000
##  Mean   : 6.881
##  3rd Qu.: 9.000
##  Max.   :12.000
```

# Number of samples per experimental group

This table shows the number of measurements for every combination of glucose concentration and time.

```
with(data, table(Glucose, Time))

##        Time
## Glucose  3  6  9 12
##     5.5 33 27 33 16
##     20  33 27 33 16
```

# Scheirer-Ray-Hare test

The Scheirer-Ray-Hare test is a non-parametric alternative to a two-way ANOVA.

The test evaluates:

1. The effect of **D-Glucose concentration**
2. The effect of **Time**
3. The **interaction between D-Glucose concentration and Time**

```
srh_result <- scheirerRayHare(
  Measurement ~ Glucose + Time + Glucose:Time,
  data = data
)

##
## DV:  Measurement
## Observations:  218
## D:  1
## MS total:  3978.5

srh_result

##                 Df Sum Sq       H  p.value
## Glucose          1 575105 144.553 0.000000
## Time             3  25135   6.318 0.097136
## Glucose:Time     3  18983   4.771 0.189326
## Residuals      210 244112
```

# Interpretation of the results

The Scheirer-Ray-Hare output contains a p-value for:

- `Glucose`
- `Time`
- `Glucose:Time`

A p-value below 0.05 is commonly considered statistically significant.

```
srh_result %>%
  mutate(
    Significance = case_when(
      `p.value` < 0.001 ~ "***",
      `p.value` < 0.01  ~ "**",
      `p.value` < 0.05  ~ "*",
      TRUE              ~ "ns"
    )
  )

##                 Df Sum Sq       H  p.value Significance
## Glucose          1 575105 144.553 0.000000            1
```

```
## Time             3  25135   6.318 0.097136            2
## Glucose:Time     3  18983   4.771 0.189326            2
## Residuals      210 244112                             2
```

## Boxplot: Measurements by glucose concentration

This plot shows the distribution of measurements for each glucose concentration.

```
ggplot(
  data,
  aes(
    x = Glucose,
    y = Measurement
  )
) +
  geom_boxplot(
    outlier.shape = NA
  ) +
  geom_jitter(
    width = 0.15,
    alpha = 0.7
  ) +
  labs(
    title = "Measurements by D-Glucose Concentration",
    x = "D-Glucose concentration (mM)",
    y = "Measurement"
  ) +
  theme_classic()
```

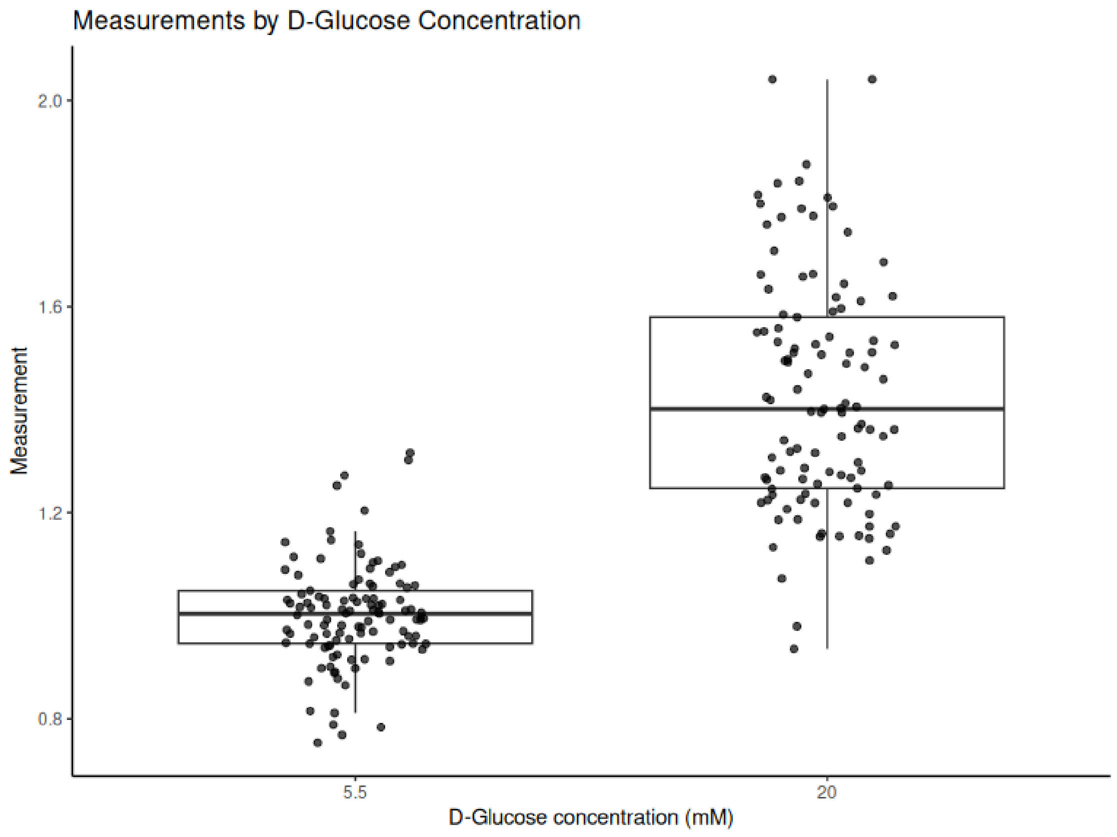


## Boxplot: Measurements by time

This plot shows the distribution of measurements at each time point.

```
ggplot(
  data,
  aes(
    x = Time,
    y = Measurement
  )
) +
  geom_boxplot(
    outlier.shape = NA
  ) +
  geom_jitter(
    width = 0.15,
    alpha = 0.7
  ) +
  labs(
    title = "Measurements by Time",
    x = "Time (h)",
    y = "Measurement"
  ) +
  theme_classic()
```

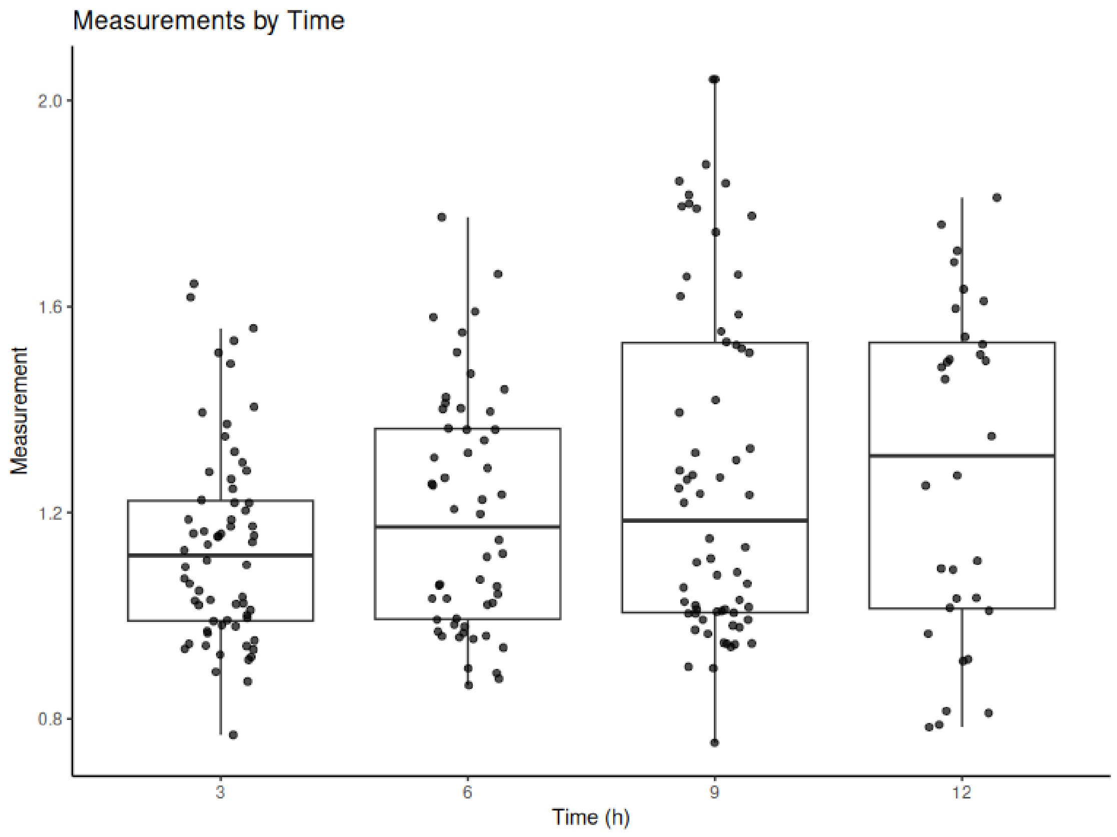


## Boxplot: Glucose concentration and time

This plot displays the measurements for every combination of glucose concentration and time.

```
ggplot(
  data,
  aes(
    x = Glucose,
    y = Measurement,
    group = interaction(Glucose, Time)
  )
) +
  geom_boxplot(
    outlier.shape = NA
  ) +
  geom_jitter(
    aes(shape = Time),
    width = 0.15,
    alpha = 0.7
  ) +
  facet_wrap(
    ~ Time
  ) +
  labs(
```

```
    title = "Measurements by D-Glucose Concentration and Time",
    x = "D-Glucose concentration (mM)",
    y = "Measurement"
  ) +
  theme_classic()
```

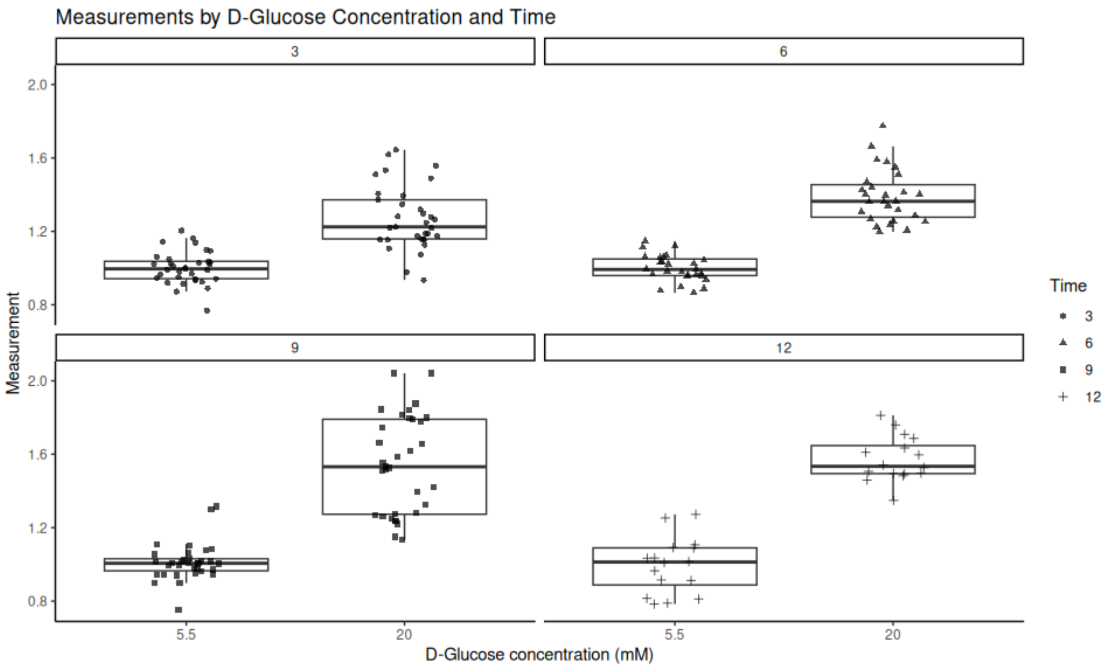


# Alternative combined boxplot

This plot places all experimental groups on one x-axis.

```
data <- data %>%
  mutate(
    Group = interaction(
      Glucose,
      Time,
      sep = " | "
    )
  )

ggplot(
  data,
  aes(
    x = Group,
    y = Measurement
  )
) +
  geom_boxplot(
    outlier.shape = NA
  ) +
  geom_jitter(
```

```
    width = 0.15,
    alpha = 0.7
  ) +
  labs(
    title = "Measurements by Experimental Group",
    x = "D-Glucose concentration | Time",
    y = "Measurement"
  ) +
  theme_classic() +
  theme(
    axis.text.x = element_text(
      angle = 45,
      hjust = 1
    )
  )
```

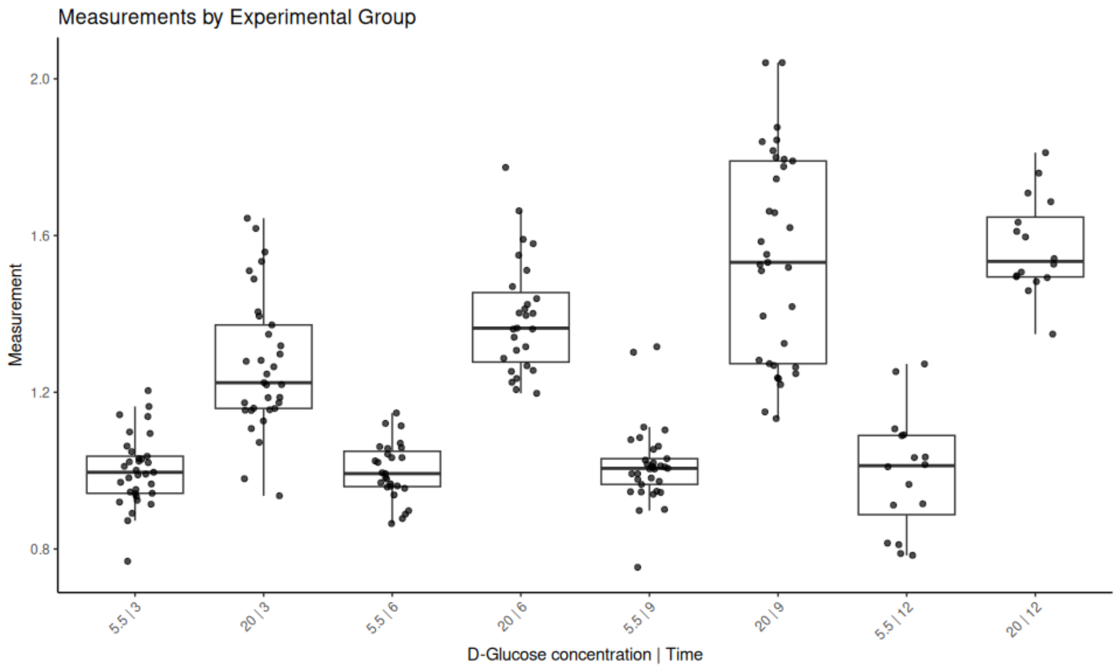


# Summary statistics

Calculate descriptive statistics for each glucose concentration and time combination.

```
summary_statistics <- data %>%
  group_by(
    Glucose,
    Time
  ) %>%
  summarise(
    N = n(),
    Mean = mean(Measurement),
    SD = sd(Measurement),
    Median = median(Measurement),
```

```
    Minimum = min(Measurement),
    Maximum = max(Measurement),
    .groups = "drop"
  )

summary_statistics

## # A tibble: 8 × 8
##   Glucose Time      N  Mean     SD Median Minimum Maximum
##   <fct>   <fct> <int> <dbl>  <dbl>  <dbl>   <dbl>   <dbl>
## 1 5.5     3        33 1.00  0.0898  0.996   0.769    1.20
## 2 5.5     6        27 0.999 0.0732  0.992   0.865    1.15
## 3 5.5     9        33 1.01  0.102   1.01    0.753    1.32
## 4 5.5     12       16 0.994 0.152   1.01    0.784    1.27
## 5 20      3        33 1.27  0.173   1.22    0.936    1.64
## 6 20      6        27 1.39  0.145   1.36    1.20     1.77
## 7 20      9        33 1.54  0.266   1.53    1.13     2.04
## 8 20      12       16 1.57  0.123   1.53    1.35     1.81
```

# Save results

Optionally save the statistical results and summary statistics.

```
write.csv(
  srh_result,
  "Scheirer_Ray_Hare_results.csv",
  row.names = FALSE
)

write.csv(
  summary_statistics,
  "summary_statistics.csv",
  row.names = FALSE
)
```

# Session information

```
sessionInfo()

## R version 4.5.0 (2025-04-11)
## Platform: x86_64-pc-linux-gnu
## Running under: Ubuntu 24.04 LTS
##
## Matrix products: default
## BLAS:   /usr/lib/x86_64-linux-gnu/openblas-pthread/libblas.so.3
## LAPACK: /usr/lib/x86_64-linux-gnu/openblas-pthread/libopenblasp-r0.3.26.so
;  LAPACK version 3.12.0
##
## locale:
##  [1] LC_CTYPE=en_US.UTF-8       LC_NUMERIC=C
```

```
##  [3] LC_TIME=en_US.UTF-8        LC_COLLATE=en_US.UTF-8
##  [5] LC_MONETARY=en_US.UTF-8    LC_MESSAGES=en_US.UTF-8
##  [7] LC_PAPER=en_US.UTF-8       LC_NAME=C
##  [9] LC_ADDRESS=C               LC_TELEPHONE=C
## [11] LC_MEASUREMENT=en_US.UTF-8 LC_IDENTIFICATION=C
##
## time zone: Etc/UTC
## tzcode source: system (glibc)
##
## attached base packages:
## [1] stats     graphics  grDevices utils     datasets  methods   base
##
## other attached packages:
## [1] dplyr_1.1.4      ggplot2_3.5.2    rcompanion_2.5.0 readxl_1.4.5
##
## loaded via a namespace (and not attached):
##  [1] gld_2.6.7           gtable_0.3.6        xfun_0.52
##  [4] coin_1.4-3          lattice_0.22-7      tzdb_0.5.0
##  [7] generics_0.1.4      vctrs_0.6.5         tools_4.5.0
## [10] stats4_4.5.0        parallel_4.5.0      sandwich_3.1-1
## [13] tibble_3.3.0        proxy_0.4-27        pkgconfig_2.0.3
## [16] Matrix_1.7-3        data.table_1.17.4   RColorBrewer_1.1-3
## [19] lifecycle_1.0.4     rootSolve_1.8.2.4   compiler_4.5.0
## [22] farver_2.1.2        Exact_3.3           codetools_0.2-20
## [25] htmltools_0.5.8.1   DescTools_0.99.60   class_7.3-23
## [28] yaml_2.3.10         pillar_1.10.2       MASS_7.3-65
## [31] boot_1.3-31         multcomp_1.4-28     tidyselect_1.2.1
## [34] digest_0.6.37       nortest_1.0-4       mvtnorm_1.3-3
## [37] labeling_0.4.3      forcats_1.0.0       splines_4.5.0
## [40] fastmap_1.2.0       grid_4.5.0          lmom_3.2
## [43] expm_1.0-0          cli_3.6.5           magrittr_2.0.3
## [46] utf8_1.2.6          survival_3.8-3      TH.data_1.1-3
## [49] e1071_1.7-16        readr_2.1.5         libcoin_1.0-10
## [52] withr_3.0.2         scales_1.4.0        rmarkdown_2.29
## [55] httr_1.4.7          matrixStats_1.5.0   cellranger_1.1.0
## [58] zoo_1.8-14          modeltools_0.2-24   hms_1.1.3
## [61] evaluate_1.0.3      knitr_1.50          haven_2.5.5
## [64] lmtest_0.9-40       rlang_1.1.6         Rcpp_1.0.14
## [67] glue_1.8.0          rstudioapi_0.17.1   R6_2.6.1
## [70] plyr_1.8.9          fs_1.6.6            multcompView_0.1-10
```

**Table S7.** Time-dependent effect of 20 mM D-glucose on luciferase activity in *MORG1*-luciferase-tagged HEK293 cells. Differences among treatment groups were analysed using the Kruskal–Wallis one-way analysis of variance by ranks, followed by Dunn's multiple-comparison post-hoc test. Among the statistically significant differences, those between the 5.5 mM D-glucose and 20 mM D-glucose treatment groups at 3, 6, 9, and 12 h are specifically indicated by underlining and are shown on the graph in Figure 4.

**Kruskal-Wallis One Way Analysis of Variance on Ranks**

**Data source:** Luciferase assays _Time dependent .JNB

**Normality Test (Shapiro-Wilk):** Failed (P < 0.050)

| Group | N | Missing | Median | 25% | 75% |
|---|---|---|---|---|---|
| 3 h-5.5 mM | 33 | 3 | 0.999 | 0.945 | 1.040 |
| 3 h-20 mM | 33 | 3 | 1.235 | 1.159 | 1.397 |
| 6 h-5.5 mM | 34 | 4 | 0.988 | 0.951 | 1.057 |
| 6 h-20 mM | 34 | 4 | 1.361 | 1.255 | 1.447 |
| 9 h-5.5 mM | 30 | 4 | 1.005 | 0.947 | 1.056 |
| 9 h -20 mM | 30 | 4 | 1.568 | 1.377 | 1.791 |
| 12 h -5.5 mM | 26 | 3 | 1.012 | 0.915 | 1.089 |
| 12 h -20 mM | 26 | 3 | 1.507 | 1.348 | 1.686 |

H = 153.801 with 7 degrees of freedom. (P = <0.001)

The differences in the median values among the treatment groups are greater than would be expected by chance; there is a statistically significant difference (P = <0.001)

To isolate the group or groups that differ from the others use a multiple comparison procedure.

**All Pairwise Multiple Comparison Procedures (Dunn's Method) :**

| Comparison | Diff of Ranks | Q | P | P<0.050 |
|---|---|---|---|---|
| 9 h -20 mM vs 6 h-5.5 mM | 126.556 | 7.488 | <0.001 | Yes |
| 9 h -20 mM vs 3 h-5.5 mM | 125.256 | 7.411 | <0.001 | Yes |
| 9 h -20 mM vs 9 h-5.5 mM | 122.308 | 6.991 | <0.001 | Yes |
| 9 h -20 mM vs 12 h -5.5 mM | 122.293 | 6.773 | <0.001 | Yes |
| 9 h -20 mM vs 3 h-20 mM | 45.123 | 2.670 | 0.212 | No |
| 9 h -20 mM vs 6 h-20 mM | 29.056 | 1.719 | 1.000 | Do Not Test |
| 9 h -20 mM vs 12 h -20 mM | 5.423 | 0.300 | 1.000 | Do Not Test |
| 12 h -20 mM vs 6 h-5.5 mM | 121.133 | 6.929 | <0.001 | Yes |
| 12 h -20 mM vs 3 h-5.5 mM | 119.833 | 6.855 | <0.001 | Yes |
| 12 h -20 mM vs 9 h-5.5 mM | 116.885 | 6.474 | <0.001 | Yes |
| 12 h -20 mM vs 12 h -5.5 mM | 116.870 | 6.283 | <0.001 | Yes |
| 12 h -20 mM vs 3 h-20 mM | 39.700 | 2.271 | 0.648 | Do Not Test |
| 12 h -20 mM vs 6 h-20 mM | 23.633 | 1.352 | 1.000 | Do Not Test |
| 6 h-20 mM vs 6 h-5.5 mM | 97.500 | 5.987 | <0.001 | Yes |
| 6 h-20 mM vs 3 h-5.5 mM | 96.200 | 5.907 | <0.001 | Yes |
| 6 h-20 mM vs 9 h-5.5 mM | 93.251 | 5.518 | <0.001 | Yes |
| 6 h-20 mM vs 12 h -5.5 mM | 93.236 | 5.333 | <0.001 | Yes |
| 6 h-20 mM vs 3 h-20 mM | 16.067 | 0.987 | 1.000 | Do Not Test |
| 3 h-20 mM vs 6 h-5.5 mM | 81.433 | 5.000 | <0.001 | Yes |
| 3 h-20 mM vs 3 h-5.5 mM | 80.133 | 4.920 | <0.001 | Yes |
| 3 h-20 mM vs 9 h-5.5 mM | 77.185 | 4.567 | <0.001 | Yes |

| | | | | |
|---|---|---|---|---|
| 3 h-20 mM vs 12 h -5.5 mM | 77.170 | 4.414 | <0.001 | Yes |
| 12 h -5.5 mM vs 6 h-5.5 mM | 4.264 | 0.244 | 1.000 | No |
| 12 h -5.5 mM vs 3 h-5.5 mM | 2.964 | 0.170 | 1.000 | Do Not Test |
| 12 h -5.5 mM vs 9 h-5.5 mM | 0.0151 | 0.000834 | 1.000 | Do Not Test |
| 9 h-5.5 mM vs 6 h-5.5 mM | 4.249 | 0.251 | 1.000 | Do Not Test |
| 9 h-5.5 mM vs 3 h-5.5 mM | 2.949 | 0.174 | 1.000 | Do Not Test |
| 3 h-5.5 mM vs 6 h-5.5 mM | 1.300 | 0.0798 | 1.000 | Do Not Test |

Note: The multiple comparisons on ranks do not include an adjustment for ties.

**Table S8**. Scheirer-Ray-Hare Analyses of the time dependent effect of 20 mM D-glucose on *MORG1* mRNA expression in *MORG1*-luciferase tagged HEK293 cells. The analyses were performed using a Scheirer-Ray-Hare Analysis as a non-parametric alternative to a two-way ANOVA.

# Scheirer-Ray-Hare Analysis

RM

2026-08-31

## Table of Contents


Setup ........ 1
Install and load required packages ........ 1
Import data ........ 2
Check column names ........ 2
Clean and prepare the data ........ 3
Data structure ........ 4
Number of samples per experimental group ........ 4
Scheirer-Ray-Hare test ........ 5
Interpretation of the results ........ 5
Boxplot: Measurements by glucose concentration ........ 6
Boxplot: Measurements by time ........ 7
Boxplot: Glucose concentration and time ........ 8
Alternative combined boxplot ........ 9
Summary statistics ........ 10
Save results ........ 11
Session information ........ 11


## Setup

## Install and load required packages

Install the packages below if they are not already installed.

```
required_packages <- c(
  "readxl",
```

```
  "rcompanion",
  "ggplot2",
  "dplyr"
)

installed <- required_packages %in% rownames(installed.packages())

if (any(!installed)) {
  install.packages(required_packages[!installed])
}

library(readxl)
library(rcompanion)
library(ggplot2)
library(dplyr)
```

# Import data

Change "your_data.xlsx" to the name or full path of your Excel file.

```
data <- read_excel("Time dependent RT-PCR of MORG1 expression (1).xlsx")
#data <- read_excel("Time dependent MORG1- Luciferase assays (1).xlsx")

head(data)

## # A tibble: 6 × 4
##   `Samples #` `D-Glucose concentration  (mM)` `Time (h)` Measurements
##         <dbl> <chr>                           <chr>             <dbl>
## 1           1 5.5 mM                          3 h               0.894
## 2           2 5.5 mM                          3 h               1.08
## 3           3 5.5 mM                          3 h               0.978
## 4           4 5.5 mM                          3 h               1.34
## 5           5 5.5 mM                          3 h               0.978
## 6           6 5.5 mM                          3 h               0.811
```

## Check column names

```
names(data)

## [1] "Samples #"                       "D-Glucose concentration  (mM)"
## [3] "Time (h)"                        "Measurements"
```

The expected Excel structure is:

| Samples # | D-Glucose concentration (mM) | Time (h) | Measurements |
|---|---|---|---|
| 1 | 5.5 mM | 3 h | 0.89378516 |
| 2 | 5.5 mM | 3 h | 1.07773315 |
| 3 | 5.5 mM | 3 h | 0.97806347 |

# Clean and prepare the data

This section renames the columns and removes units such as `mM` and `h`.

```
# Rename columns
names(data) <- c(
  "Sample",
  "Glucose",
  "Time",
  "Measurement"
)

# Extract numeric glucose concentration
data$Glucose_numeric <- as.numeric(
  gsub("[^0-9.]", "", data$Glucose)
)

# Extract numeric time
data$Time_numeric <- as.numeric(
  gsub("[^0-9.]", "", data$Time)
)

# Convert independent variables to factors
data$Glucose <- factor(data$Glucose_numeric)

data$Time <- factor(
  data$Time_numeric,
  levels = sort(unique(data$Time_numeric))
)

# Ensure measurement is numeric
data$Measurement <- as.numeric(data$Measurement)

# Remove rows containing missing values
data <- data %>%
  filter(
    !is.na(Glucose),
    !is.na(Time),
    !is.na(Measurement)
  )

# Inspect cleaned data
head(data)
```

```
## # A tibble: 6 × 6
##   Sample Glucose Time  Measurement Glucose_numeric Time_numeric
##    <dbl> <fct>   <fct>       <dbl>           <dbl>        <dbl>
## 1      1 5.5     3           0.894             5.5            3
## 2      2 5.5     3           1.08              5.5            3
## 3      3 5.5     3           0.978             5.5            3
```

```
## 4      4 5.5     3           1.34              5.5            3
## 5      5 5.5     3           0.978             5.5            3
## 6      6 5.5     3           0.811             5.5            3
```

# Data structure

```
str(data)

## tibble [140 × 6] (S3: tbl_df/tbl/data.frame)
##  $ Sample         : num [1:140] 1 2 3 4 5 6 7 8 9 10 ...
##  $ Glucose        : Factor w/ 2 levels "5.5","20": 1 1 1 1 1 1 1 1 1 1 ...
##  $ Time           : Factor w/ 4 levels "3","6","9","12": 1 1 1 1 1 1 1 1 1
1 ...
##  $ Measurement    : num [1:140] 0.894 1.078 0.978 1.336 0.978 ...
##  $ Glucose_numeric: num [1:140] 5.5 5.5 5.5 5.5 5.5 5.5 5.5 5.5 5.5 5.5 ..
.
##  $ Time_numeric   : num [1:140] 3 3 3 3 3 3 3 3 3 3 ...

summary(data)

##      Sample       Glucose   Time     Measurement     Glucose_numeric
##  Min.   : 1.000   5.5:70   3 :34   Min.   :0.4625   Min.   : 5.50
##  1st Qu.: 5.000   20 :70   6 :35   1st Qu.:0.9796   1st Qu.: 5.50
##  Median : 9.000            9 :36   Median :1.1684   Median :12.75
##  Mean   : 9.264            12:35   Mean   :1.2926   Mean   :12.75
##  3rd Qu.:14.000                    3rd Qu.:1.4009   3rd Qu.:20.00
##  Max.   :18.000                    Max.   :5.7998   Max.   :20.00
##   Time_numeric
##  Min.   : 3.000
##  1st Qu.: 6.000
##  Median : 9.000
##  Mean   : 7.543
##  3rd Qu.: 9.750
##  Max.   :12.000
```

# Number of samples per experimental group

This table shows the number of measurements for every combination of glucose concentration and time.

```
with(data, table(Glucose, Time))

##        Time
## Glucose  3  6  9 12
##     5.5 16 18 18 18
##     20  18 17 18 17
```

# Scheirer-Ray-Hare test

The Scheirer-Ray-Hare test is a non-parametric alternative to a two-way ANOVA.

The test evaluates:

1. The effect of **D-Glucose concentration**
2. The effect of **Time**
3. The **interaction between D-Glucose concentration and Time**

```
srh_result <- scheirerRayHare(
  Measurement ~ Glucose + Time + Glucose:Time,
  data = data
)

##
## DV:  Measurement
## Observations:  140
## D:  0.9999606
## MS total:  1645

srh_result

##                 Df Sum Sq      H p.value
## Glucose          1  97256 59.125 0.00000
## Time             3   3220  1.958 0.58121
## Glucose:Time     3   2756  1.676 0.64235
## Residuals      132 125411
```

# Interpretation of the results

The Scheirer-Ray-Hare output contains a p-value for:

- `Glucose`
- `Time`
- `Glucose:Time`

A p-value below 0.05 is commonly considered statistically significant.

```
srh_result %>%
  mutate(
    Significance = case_when(
      `p.value` < 0.001 ~ "***",
      `p.value` < 0.01  ~ "**",
      `p.value` < 0.05  ~ "*",
      TRUE              ~ "ns"
    )
  )

##                 Df Sum Sq      H p.value Significance
## Glucose          1  97256 59.125 0.00000            1
```

```
## Time           3   3220  1.958 0.58121          2
## Glucose:Time   3   2756  1.676 0.64235          2
## Residuals    132 125411                         2
```

## Boxplot: Measurements by glucose concentration

This plot shows the distribution of measurements for each glucose concentration.

```
ggplot(
  data,
  aes(
    x = Glucose,
    y = Measurement
  )
) +
  geom_boxplot(
    outlier.shape = NA
  ) +
  geom_jitter(
    width = 0.15,
    alpha = 0.7
  ) +
  labs(
    title = "Measurements by D-Glucose Concentration",
    x = "D-Glucose concentration (mM)",
    y = "Measurement"
  ) +
  theme_classic()
```

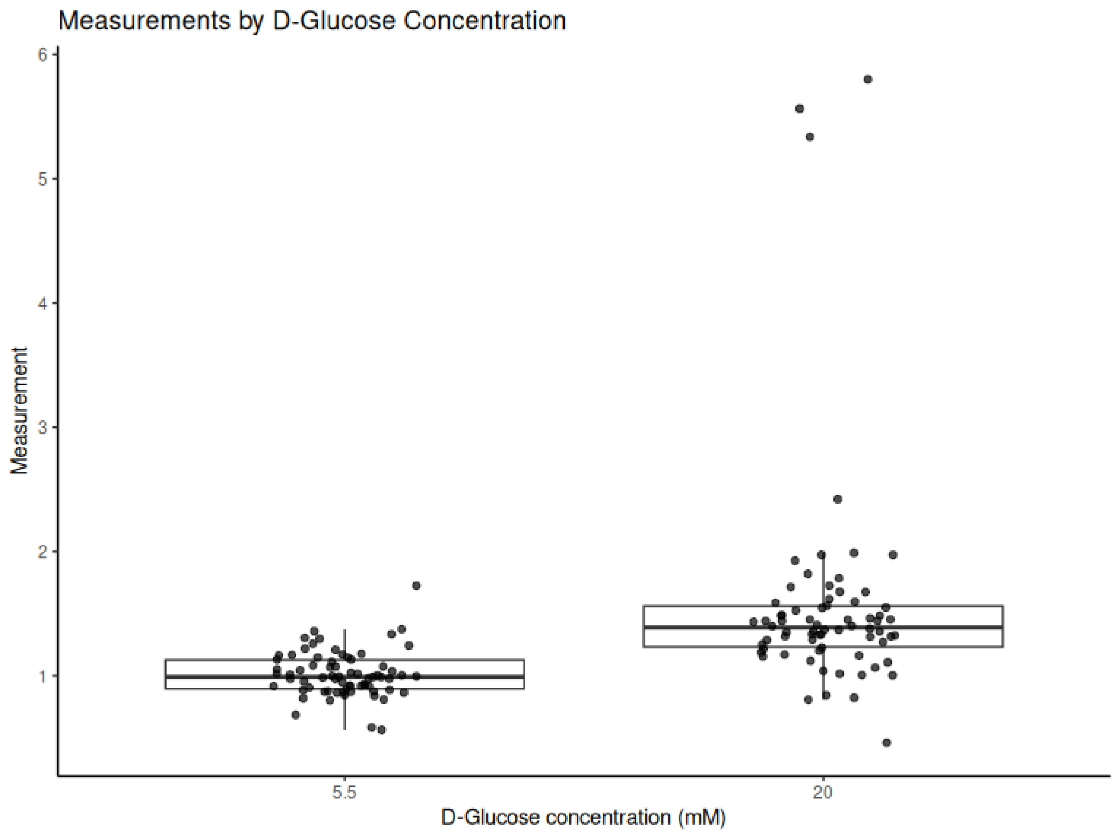


## Boxplot: Measurements by time

This plot shows the distribution of measurements at each time point.

```
ggplot(
  data,
  aes(
    x = Time,
    y = Measurement
  )
) +
  geom_boxplot(
    outlier.shape = NA
  ) +
  geom_jitter(
    width = 0.15,
    alpha = 0.7
  ) +
  labs(
    title = "Measurements by Time",
    x = "Time (h)",
    y = "Measurement"
  ) +
  theme_classic()
```

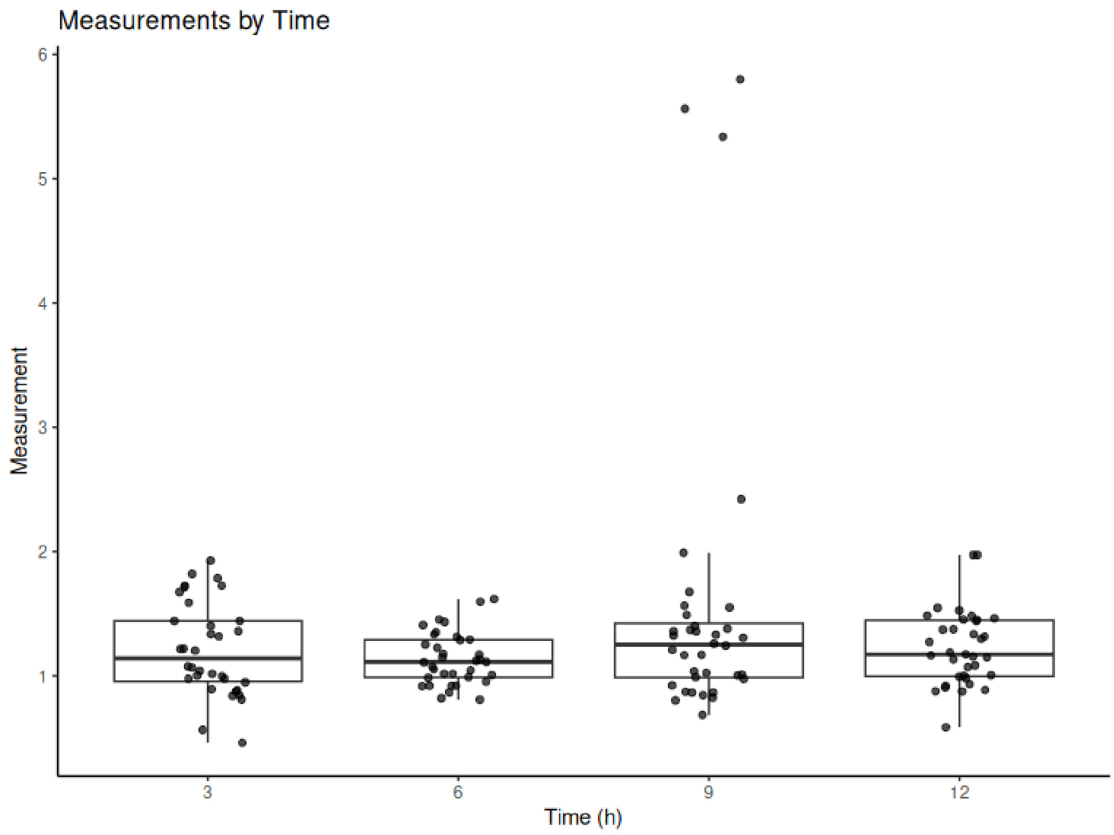


## Boxplot: Glucose concentration and time

This plot displays the measurements for every combination of glucose concentration and time.

```
ggplot(
  data,
  aes(
    x = Glucose,
    y = Measurement,
    group = interaction(Glucose, Time)
  )
) +
  geom_boxplot(
    outlier.shape = NA
  ) +
  geom_jitter(
    aes(shape = Time),
    width = 0.15,
    alpha = 0.7
  ) +
  facet_wrap(
    ~ Time
  ) +
  labs(
```

```
    title = "Measurements by D-Glucose Concentration and Time",
    x = "D-Glucose concentration (mM)",
    y = "Measurement"
  ) +
  theme_classic()
```

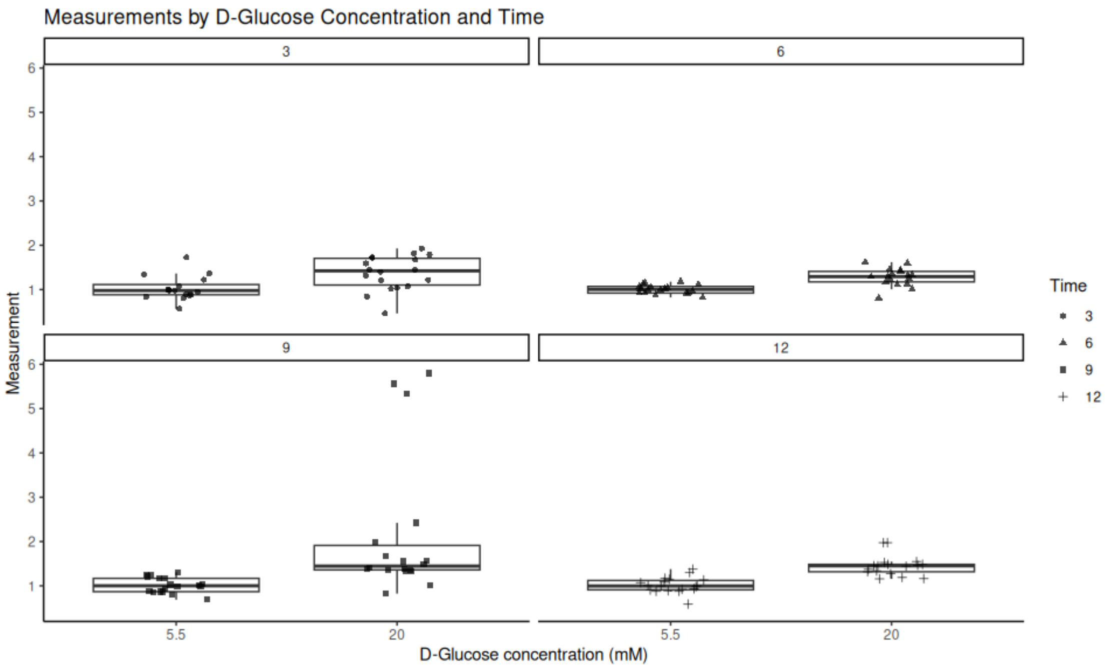


# Alternative combined boxplot

This plot places all experimental groups on one x-axis.

```
data <- data %>%
  mutate(
    Group = interaction(
      Glucose,
      Time,
      sep = " | "
    )
  )

ggplot(
  data,
  aes(
    x = Group,
    y = Measurement
  )
) +
  geom_boxplot(
    outlier.shape = NA
  ) +
  geom_jitter(
```

```
    width = 0.15,
    alpha = 0.7
  ) +
  labs(
    title = "Measurements by Experimental Group",
    x = "D-Glucose concentration | Time",
    y = "Measurement"
  ) +
  theme_classic() +
  theme(
    axis.text.x = element_text(
      angle = 45,
      hjust = 1
    )
  )
```

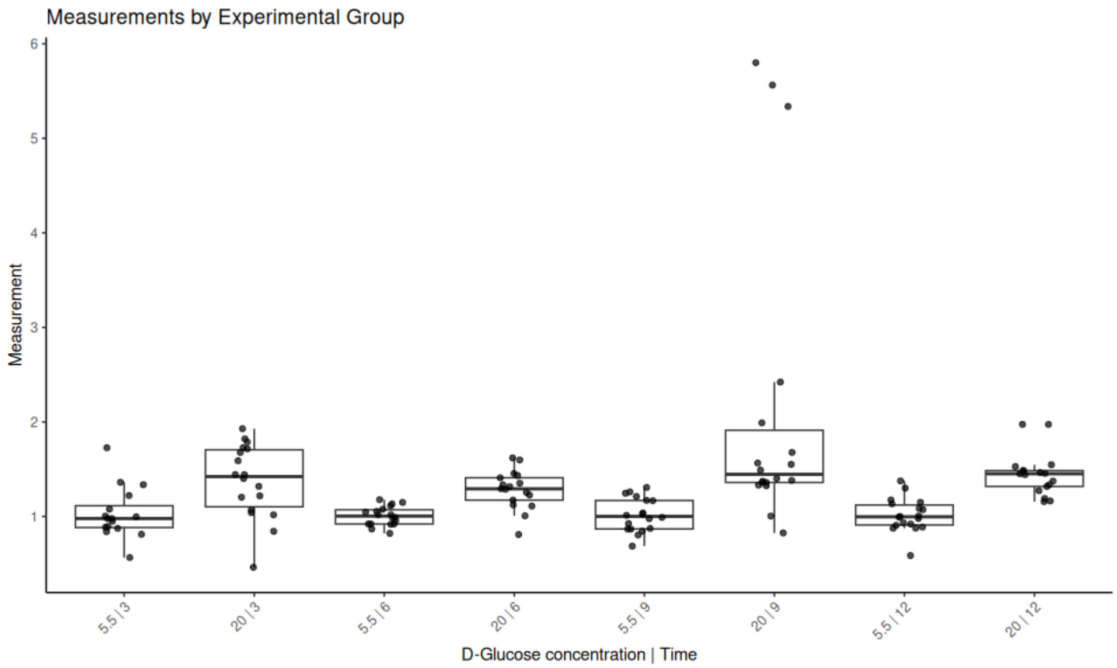


# Summary statistics

Calculate descriptive statistics for each glucose concentration and time combination.

```
summary_statistics <- data %>%
  group_by(
    Glucose,
    Time
  ) %>%
  summarise(
    N = n(),
    Mean = mean(Measurement),
    SD = sd(Measurement),
    Median = median(Measurement),
```

```
    Minimum = min(Measurement),
    Maximum = max(Measurement),
    .groups = "drop"
  )

summary_statistics
```

```
## # A tibble: 8 × 8
##   Glucose Time      N  Mean    SD Median Minimum Maximum
##   <fct>   <fct> <int> <dbl> <dbl>  <dbl>   <dbl>   <dbl>
## 1 5.5     3        16  1.03 0.271  0.978   0.565    1.73
## 2 5.5     6        18  1.00 0.101  1.00    0.821    1.18
## 3 5.5     9        18  1.01 0.178  1.00    0.686    1.31
## 4 5.5     12       18  1.01 0.179  0.997   0.587    1.38
## 5 20      3        18  1.37 0.388  1.42    0.462    1.93
## 6 20      6        17  1.28 0.203  1.29    0.809    1.62
## 7 20      9        18  2.15 1.61   1.45    0.825    5.80
## 8 20      12       17  1.45 0.233  1.45    1.16     1.97
```

# Save results

Optionally save the statistical results and summary statistics.

```
write.csv(
  srh_result,
  "Scheirer_Ray_Hare_results.csv",
  row.names = FALSE
)

write.csv(
  summary_statistics,
  "summary_statistics.csv",
  row.names = FALSE
)
```

# Session information

```
sessionInfo()
```

```
## R version 4.5.0 (2025-04-11)
## Platform: x86_64-pc-linux-gnu
## Running under: Ubuntu 24.04 LTS
##
## Matrix products: default
## BLAS:   /usr/lib/x86_64-linux-gnu/openblas-pthread/libblas.so.3
## LAPACK: /usr/lib/x86_64-linux-gnu/openblas-pthread/libopenblasp-r0.3.26.so
;  LAPACK version 3.12.0
##
## locale:
##  [1] LC_CTYPE=en_US.UTF-8       LC_NUMERIC=C
```

```
##  [3] LC_TIME=en_US.UTF-8        LC_COLLATE=en_US.UTF-8
##  [5] LC_MONETARY=en_US.UTF-8    LC_MESSAGES=en_US.UTF-8
##  [7] LC_PAPER=en_US.UTF-8       LC_NAME=C
##  [9] LC_ADDRESS=C               LC_TELEPHONE=C
## [11] LC_MEASUREMENT=en_US.UTF-8 LC_IDENTIFICATION=C
##
## time zone: Etc/UTC
## tzcode source: system (glibc)
##
## attached base packages:
## [1] stats     graphics  grDevices utils     datasets  methods   base
##
## other attached packages:
## [1] dplyr_1.1.4      ggplot2_3.5.2    rcompanion_2.5.0 readxl_1.4.5
##
## loaded via a namespace (and not attached):
##  [1] gld_2.6.7          gtable_0.3.6       xfun_0.52
##  [4] coin_1.4-3         lattice_0.22-7     tzdb_0.5.0
##  [7] generics_0.1.4     vctrs_0.6.5        tools_4.5.0
## [10] stats4_4.5.0       parallel_4.5.0     sandwich_3.1-1
## [13] tibble_3.3.0       proxy_0.4-27       pkgconfig_2.0.3
## [16] Matrix_1.7-3       data.table_1.17.4  RColorBrewer_1.1-3
## [19] lifecycle_1.0.4    rootSolve_1.8.2.4  compiler_4.5.0
## [22] farver_2.1.2       Exact_3.3          codetools_0.2-20
## [25] htmltools_0.5.8.1  DescTools_0.99.60  class_7.3-23
## [28] yaml_2.3.10        pillar_1.10.2      MASS_7.3-65
## [31] boot_1.3-31        multcomp_1.4-28    tidyselect_1.2.1
## [34] digest_0.6.37      nortest_1.0-4      mvtnorm_1.3-3
## [37] labeling_0.4.3     forcats_1.0.0      splines_4.5.0
## [40] fastmap_1.2.0      grid_4.5.0         lmom_3.2
## [43] expm_1.0-0         cli_3.6.5          magrittr_2.0.3
## [46] utf8_1.2.6         survival_3.8-3     TH.data_1.1-3
## [49] e1071_1.7-16       readr_2.1.5        libcoin_1.0-10
## [52] withr_3.0.2        scales_1.4.0       rmarkdown_2.29
## [55] httr_1.4.7         matrixStats_1.5.0  cellranger_1.1.0
## [58] zoo_1.8-14         modeltools_0.2-24  hms_1.1.3
## [61] evaluate_1.0.3     knitr_1.50         haven_2.5.5
## [64] lmtest_0.9-40      rlang_1.1.6        Rcpp_1.0.14
## [67] glue_1.8.0         rstudioapi_0.17.1  R6_2.6.1
## [70] plyr_1.8.9         fs_1.6.6           multcompView_0.1-10
```

**Table S9.** Time-dependent effect of 20 mM D-glucose on *MORG1* mRNA in *MORG1*-luciferase-tagged HEK293 cells. Differences among treatment groups were analysed using the Kruskal–Wallis one-way analysis of variance by ranks, followed by Dunn's multiple-comparison post-hoc test. Among the statistically significant differences, those between the 5.5 mM D-glucose and 20 mM D-glucose treatment groups at 3, 6, 9, and 12 h are specifically indicated by underlining and are shown on the graph in Figure 5.

**Kruskal-Wallis One Way Analysis of Variance on Ranks**

**Data source:** Time dependent RT-PCRs.JNB

**Normality Test (Shapiro-Wilk):** Failed (P < 0.050)

| Group | N | Missing | Median | 25% | 75% |
|---|---|---|---|---|---|
| 3 h -5.5 mM | 20 | 4 | 0.978 | 0.877 | 1.185 |
| 3 h -20 mM | 20 | 2 | 1.422 | 1.061 | 1.718 |
| 6 h -5.5 mM | 20 | 2 | 1.004 | 0.920 | 1.086 |
| 6 h - 20 mM | 20 | 3 | 1.292 | 1.147 | 1.422 |
| 9 h -5.5 mM | 20 | 2 | 1.001 | 0.866 | 1.180 |
| 9 h - 20 mM | 20 | 2 | 1.446 | 1.352 | 2.098 |
| 12 h -5.5 mM | 20 | 2 | 0.997 | 0.902 | 1.137 |
| 12 h - 20 mM | 19 | 2 | 1.452 | 1.295 | 1.506 |

H = 62.759 with 7 degrees of freedom. (P = <0.001)

The differences in the median values among the treatment groups are greater than would be expected by chance; there is a statistically significant difference (P = <0.001)

To isolate the group or groups that differ from the others use a multiple comparison procedure.

All Pairwise Multiple Comparison Procedures (Dunn's Method) :

| Comparison | Diff of Ranks | Q | P | P<0.050 |
|---|---|---|---|---|
| 9 h - 20 mM vs 6 h -5.5 mM | 64.250 | 4.752 | <0.001 | Yes |
| 9 h - 20 mM vs 9 h -5.5 mM | 63.278 | 4.680 | <0.001 | Yes |
| 9 h - 20 mM vs 12 h -5.5 mM | 61.583 | 4.555 | <0.001 | Yes |
| 9 h - 20 mM vs 3 h -5.5 mM | 61.521 | 4.415 | <0.001 | Yes |
| 9 h - 20 mM vs 6 h - 20 mM | 22.245 | 1.622 | 1.000 | No |
| 9 h - 20 mM vs 3 h -20 mM | 15.528 | 1.149 | 1.000 | Do Not Test |
| 9 h - 20 mM vs 12 h - 20 mM | 2.392 | 0.174 | 1.000 | Do Not Test |
| 12 h - 20 mM vs 6 h -5.5 mM | 61.858 | 4.510 | <0.001 | Yes |
| 12 h - 20 mM vs 9 h -5.5 mM | 60.886 | 4.439 | <0.001 | Yes |
| 12 h - 20 mM vs 12 h -5.5 mM | 59.191 | 4.315 | <0.001 | Yes |
| 12 h - 20 mM vs 3 h -5.5 mM | 59.129 | 4.185 | <0.001 | Yes |
| 12 h - 20 mM vs 6 h - 20 mM | 19.853 | 1.427 | 1.000 | Do Not Test |
| 12 h - 20 mM vs 3 h -20 mM | 13.136 | 0.958 | 1.000 | Do Not Test |
| 3 h -20 mM vs 6 h -5.5 mM | 48.722 | 3.604 | 0.009 | Yes |
| 3 h -20 mM vs 9 h -5.5 mM | 47.750 | 3.532 | 0.012 | Yes |
| 3 h -20 mM vs 12 h -5.5 mM | 46.056 | 3.407 | 0.018 | Yes |
| 3 h -20 mM vs 3 h -5.5 mM | 45.993 | 3.300 | 0.027 | Yes |
| 3 h -20 mM vs 6 h - 20 mM | 6.717 | 0.490 | 1.000 | Do Not Test |

| | | | | |
|---|---|---|---|---|
| 6 h - 20 mM vs 6 h -5.5 mM | 42.005 | 3.062 | 0.062 | No |
| 6 h - 20 mM vs 9 h -5.5 mM | 41.033 | 2.991 | 0.078 | Do Not Test |
| 6 h - 20 mM vs 12 h -5.5 mM | 39.338 | 2.868 | 0.116 | Do Not Test |
| 6 h - 20 mM vs 3 h -5.5 mM | 39.276 | 2.780 | 0.152 | Do Not Test |
| 3 h -5.5 mM vs 6 h -5.5 mM | 2.729 | 0.196 | 1.000 | Do Not Test |
| 3 h -5.5 mM vs 9 h -5.5 mM | 1.757 | 0.126 | 1.000 | Do Not Test |
| 3 h -5.5 mM vs 12 h -5.5 mM | 0.0625 | 0.00448 | 1.000 | Do Not Test |
| 12 h -5.5 mM vs 6 h -5.5 mM | 2.667 | 0.197 | 1.000 | Do Not Test |
| 12 h -5.5 mM vs 9 h -5.5 mM | 1.694 | 0.125 | 1.000 | Do Not Test |
| 9 h -5.5 mM vs 6 h -5.5 mM | 0.972 | 0.0719 | 1.000 | Do Not Test |

Note: The multiple comparisons on ranks do not include an adjustment for ties.

**Figure 2 c)**
Western Blots

WB # 1

The SDS-PAGE gel was transferred on a PVDF membrane and
- the upper part was probed with an antibody against VINCULIN;
- the lower part was probed with an antibody against MORG1.

- **The bands of MORG1 and VINCULIN expressions from WB #1 are shown as representative images in Fig. 2c)**

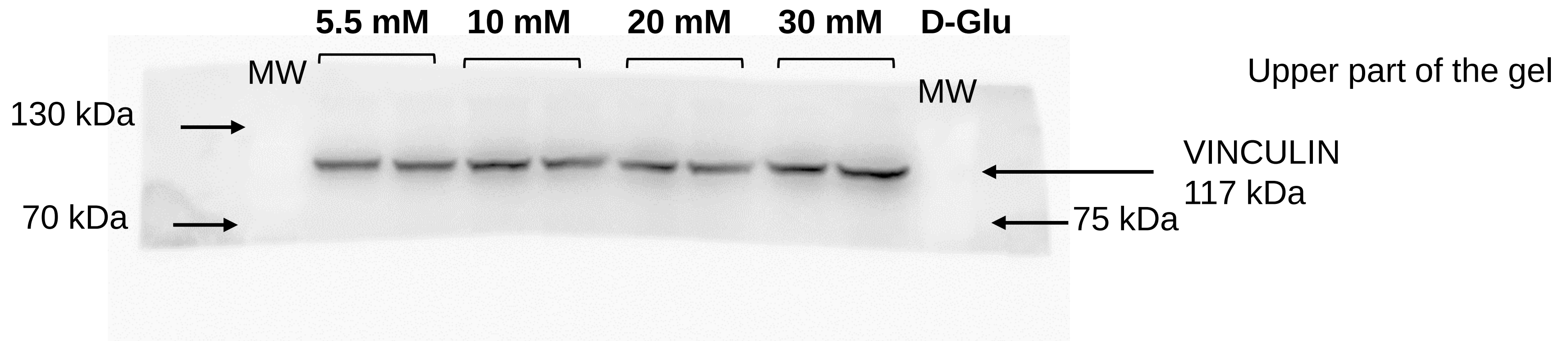


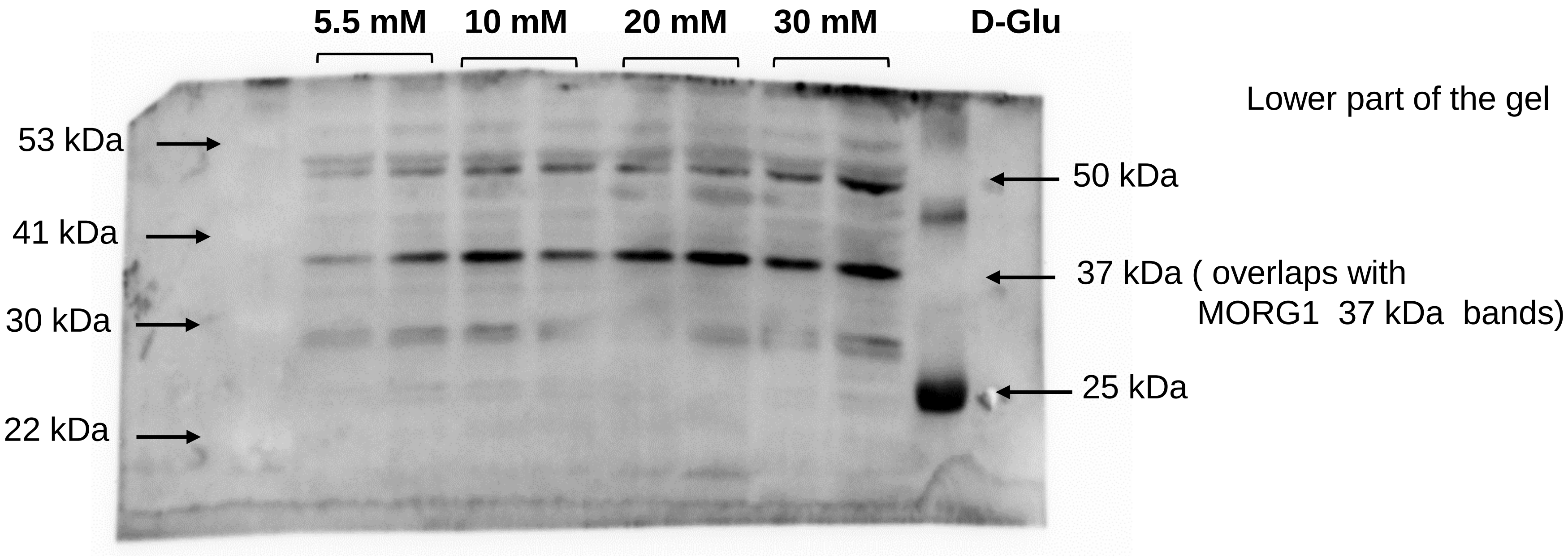

**Figure 2 c)**
Western Blots

The SDS-PAGE gel was transferred on a PVDF membrane and
- the upper part was probed with an antibody against VINCULIN;
- the lower part was probed with an antibody aginst MORG1.

WB #2

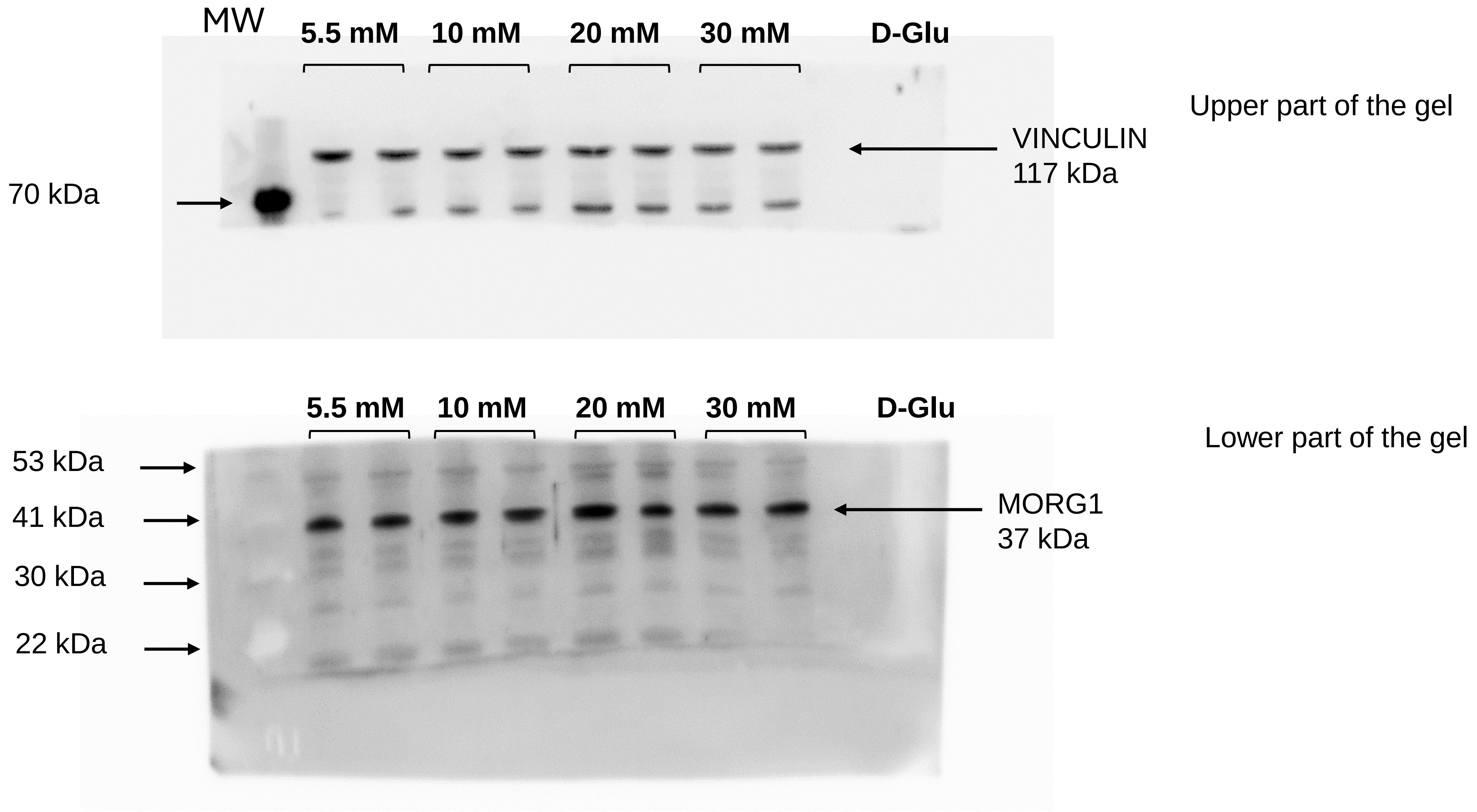

**Figure 2 c)**
Western Blots

WB # 3

The SDS-PAGE gel was transferred on a PVDF membrane and
- the upper part was probed with an antibody against VINCULIN;
- the lower part was probed with an antibody against MORG1.

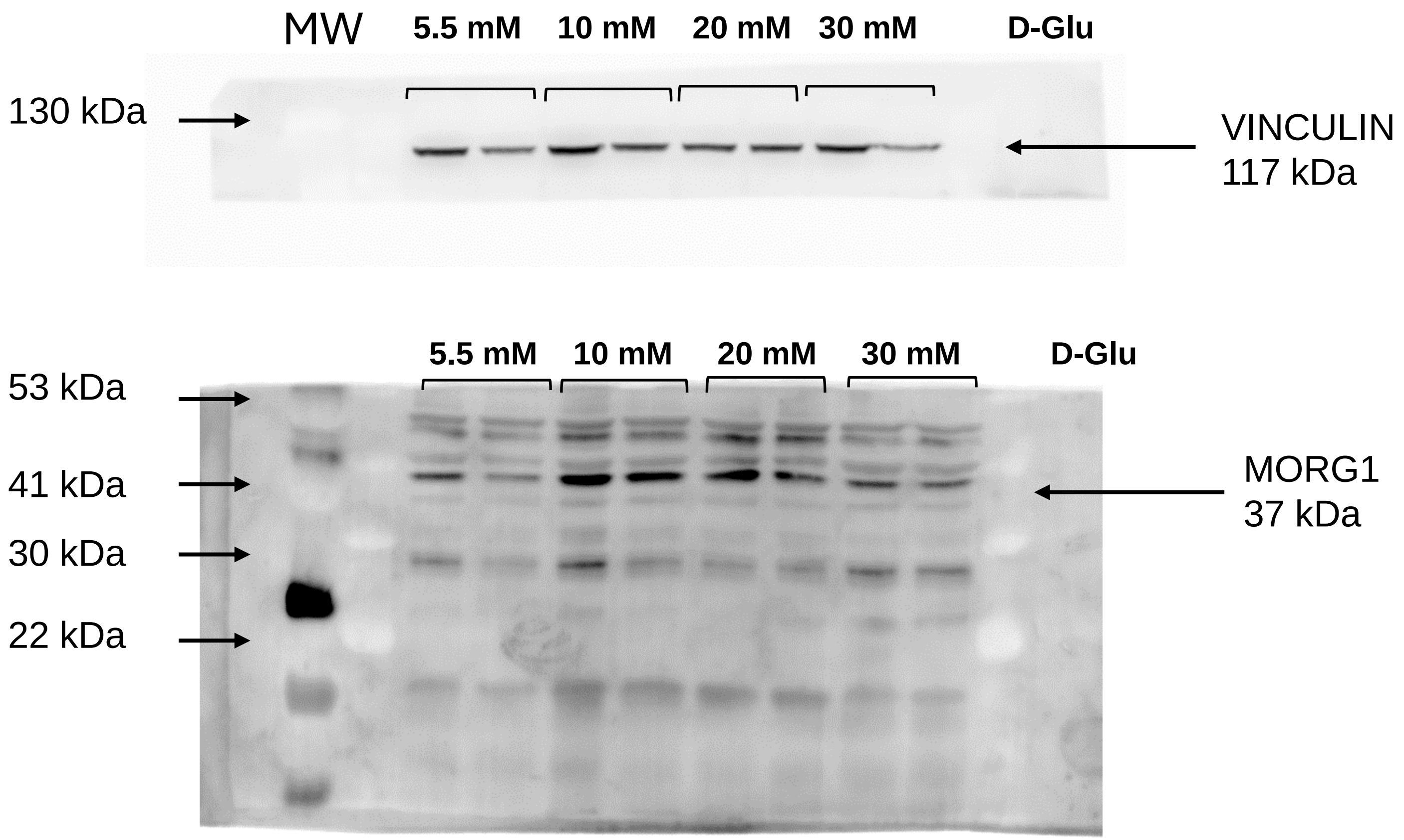


Upper part of the gel

Lower part of the gel

**Figure 6 a)**
Western Blots

**3 h treatments**

WB # 1

The SDS-PAGE gel was transferred on a PVDF membrane and
- the upper part was probed with an antibody against VINCULIN;
- the lower part was probed with an antibody against MORG1.

- **The bands selected for the representative images in Fig. 6a) are shown in the boxes respectively for VINCULIN and MORG1 proteins**.

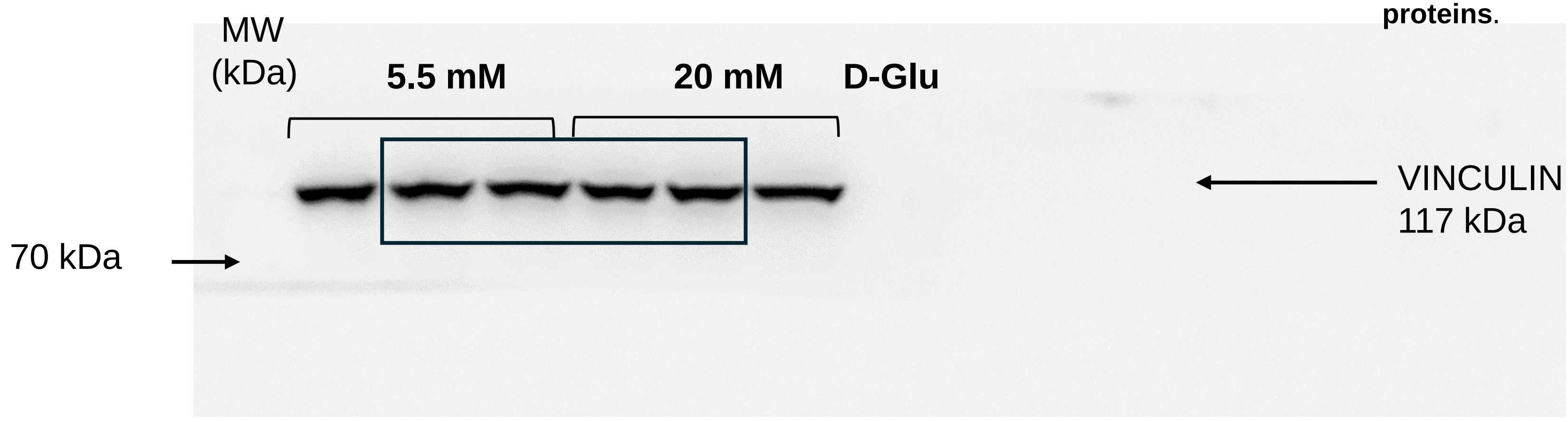


Upper part of the gel

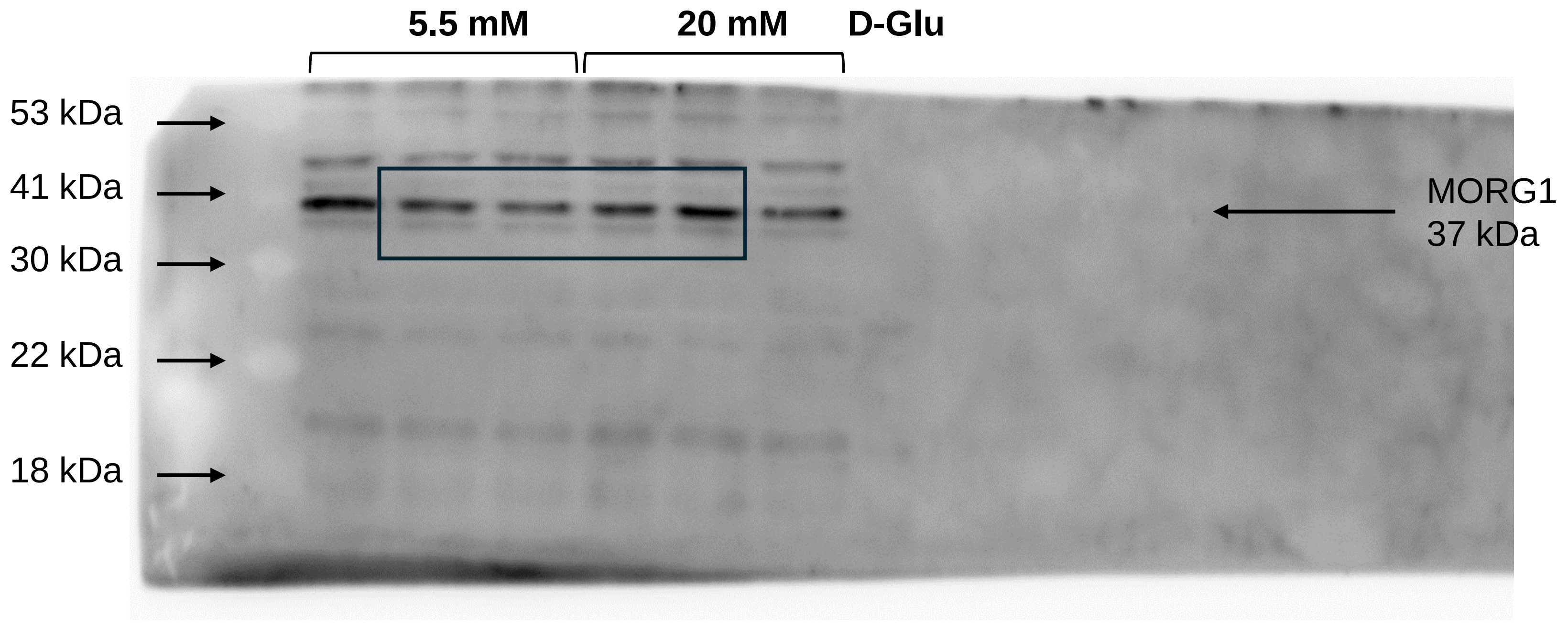


Lower part of the gel

**Figure 6 a)**
Western Blots

**3 h treatments**

WB # 2

The SDS-PAGE gel was transferred on a PVDF membrane and
- the upper part was probed with an antibody against VINCULIN;
- the lower part was probed with an antibody against MORG1.

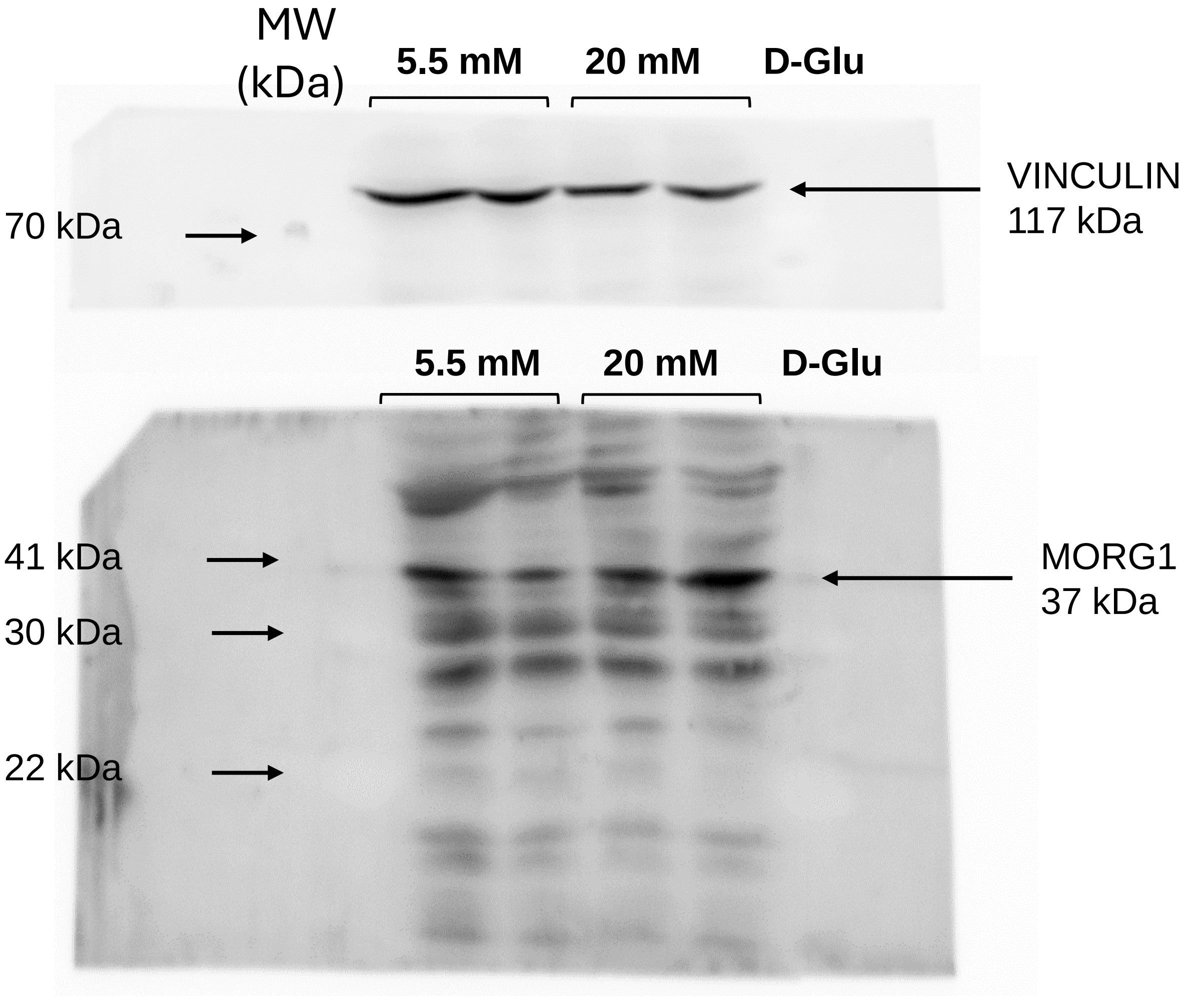


Upper part of the gel

Lower part of the gel

**Figure 6 b)**

Western Blots

**6 h treatments**

WB # 1

The SDS-PAGE gel was transferred on a PVDF membrane and

- the upper part was probed with an antibody against VINCULIN;
- the lower part was probed with an antibody against MORG1.

**- The bands of MORG1 and VINCULIN protein expressions from WB #1 are shown as representative images in Fig. 6b)**

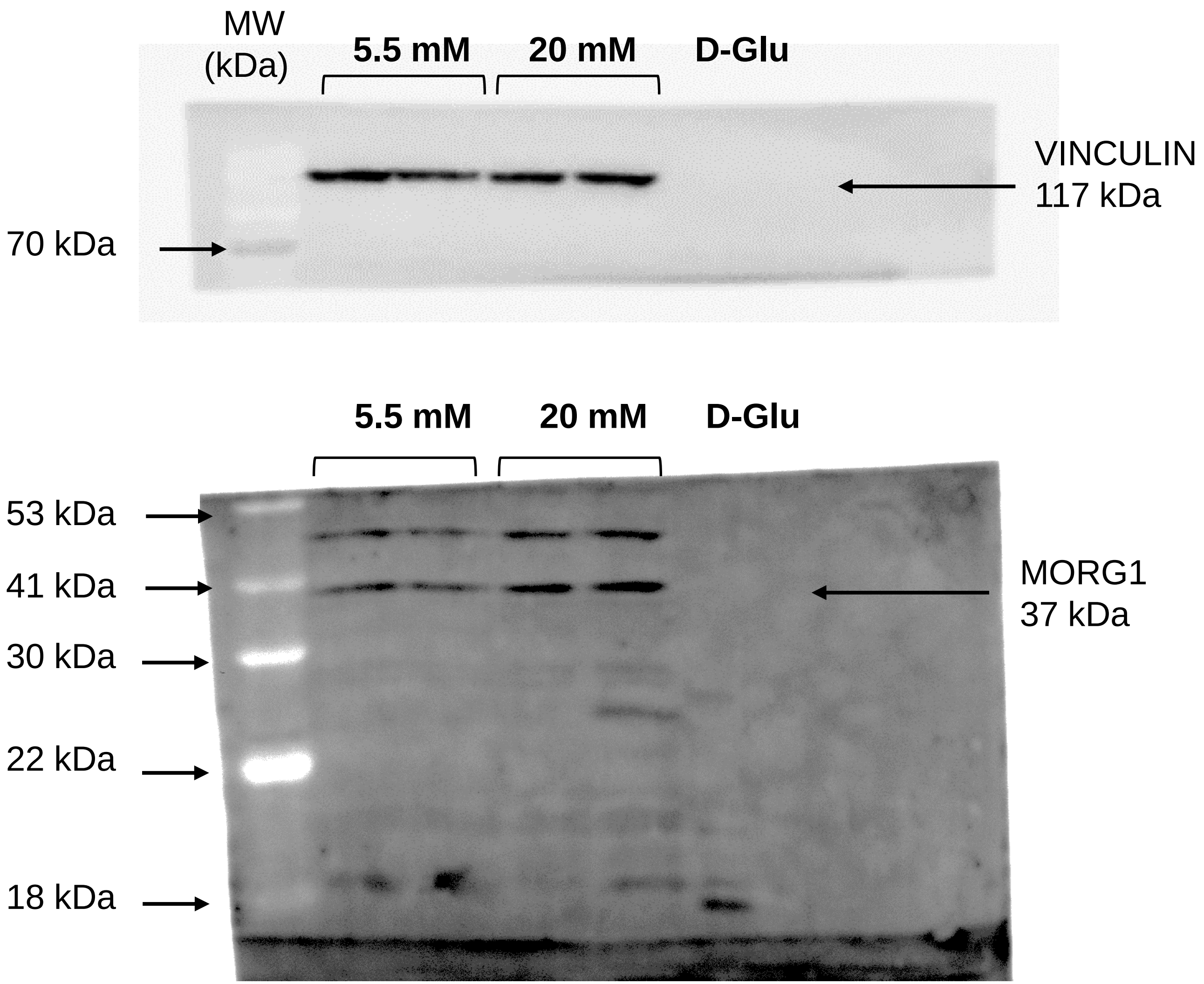


Upper part of the gel

Lower part of the gel

**Figure 6 b)**

Western Blots **6 h treatments** WB # 2

The SDS-PAGE gel was transferred on a PVDF membrane and
- the upper part was probed with an antibody against VINCULIN;
- the lower part was probed with an antibody against MORG1.

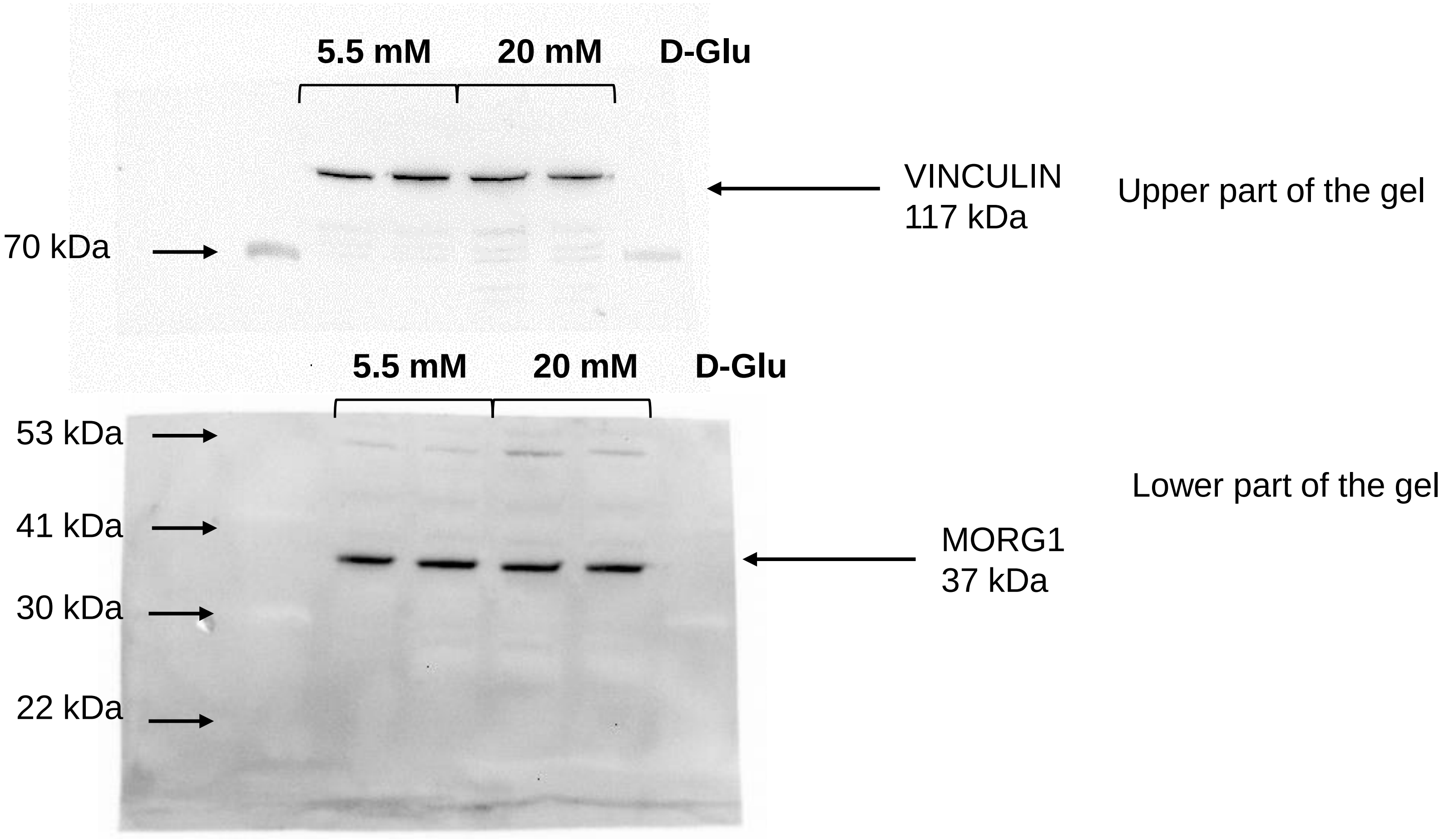

**Figure 6 c)**

Western Blots

**9 h treatments**

WB # 1

The SDS-PAGE gel was transferred on a PVDF membrane and
- the upper part was probed with an antibody against VINCULIN;
- the lower part was probed with an antibody against MORG1.

- **The lanes which were selected for the representative images shown in Fig. 6c) are shown in the boxes respectively for MORG1 and VINCULIN WBlots**.

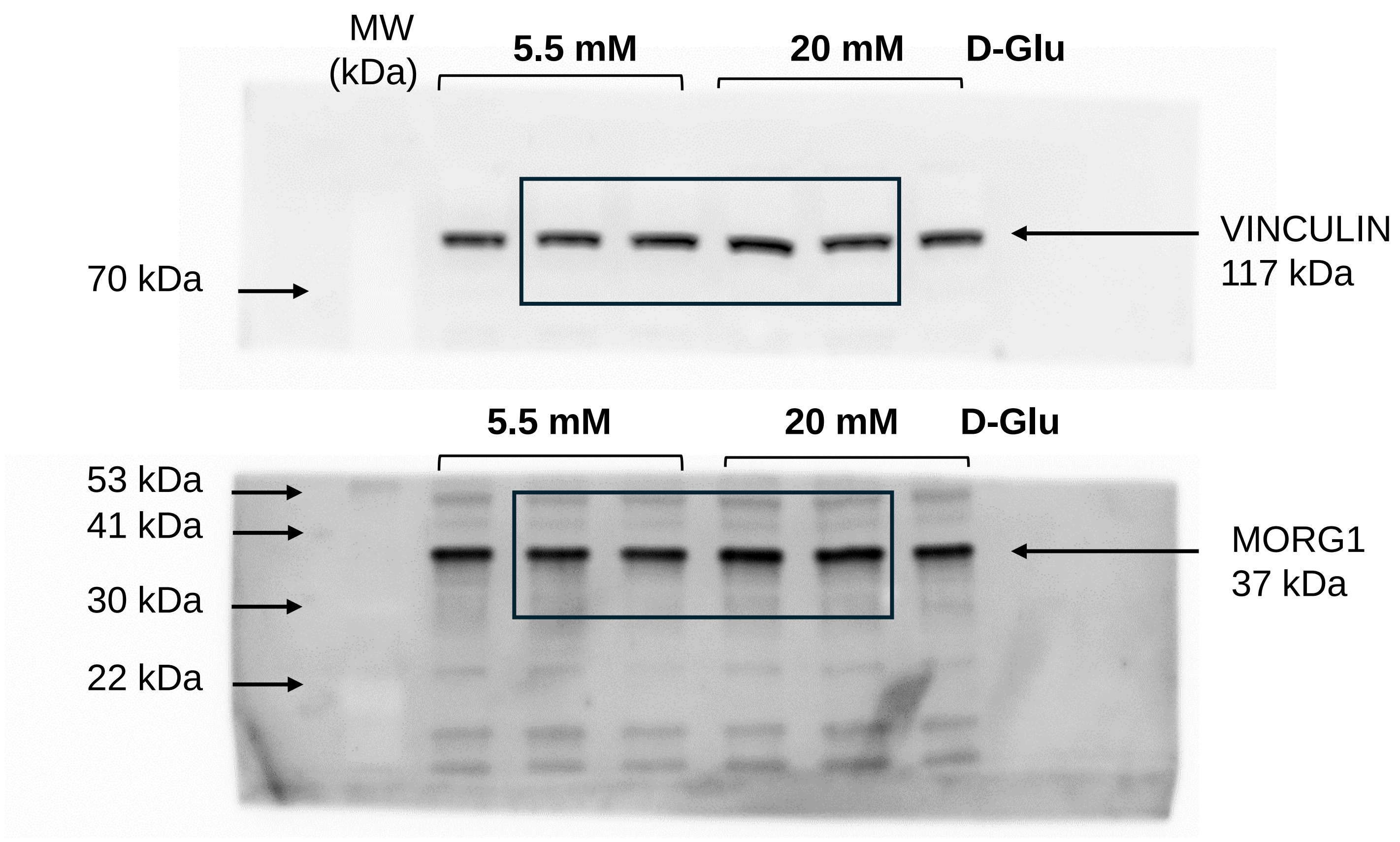


Upper part of the gel

Lower part of the gel

**Figure 6 c)**
Western Blots

**9 h treatments**

WB # 2

The SDS-PAGE gel was transferred on a PVDF membrane and
- the upper part was probed with an antibody against VINCULIN;
- the lower part was probed with an antibody against MORG1.

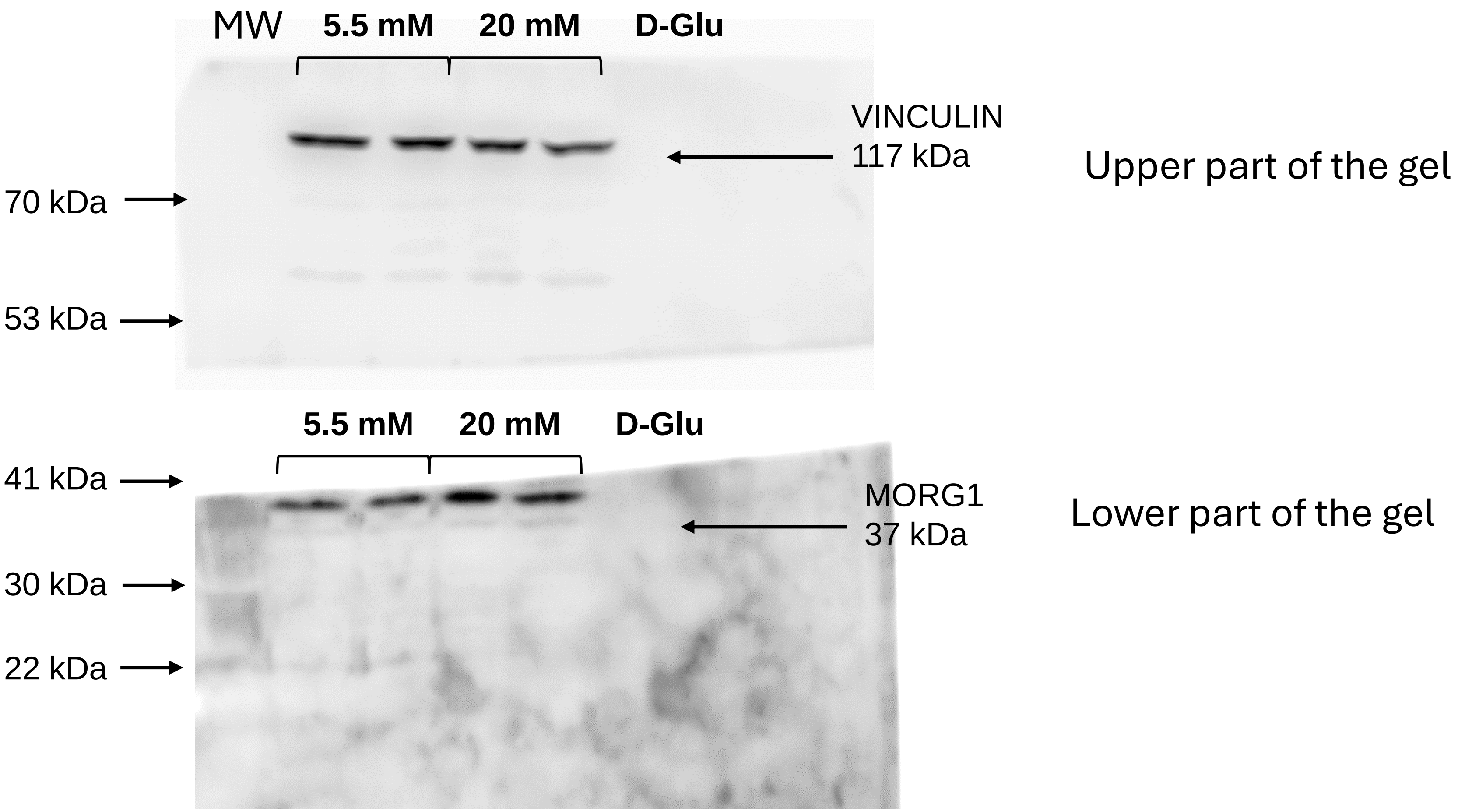

**Figure 6 d)**
Western Blots **12 h treatments** WB # 1

The SDS-PAGE gel was transferred on a PVDF membrane and
- the upper part was probed with an antibody against VINCULIN;
- the lower part was probed with an antibody against MORG1.

- **The lanes which were selected for the representative images in Fig. 6d) are shown in the box respectively for MORG1 and VINCULIN WBlots.**

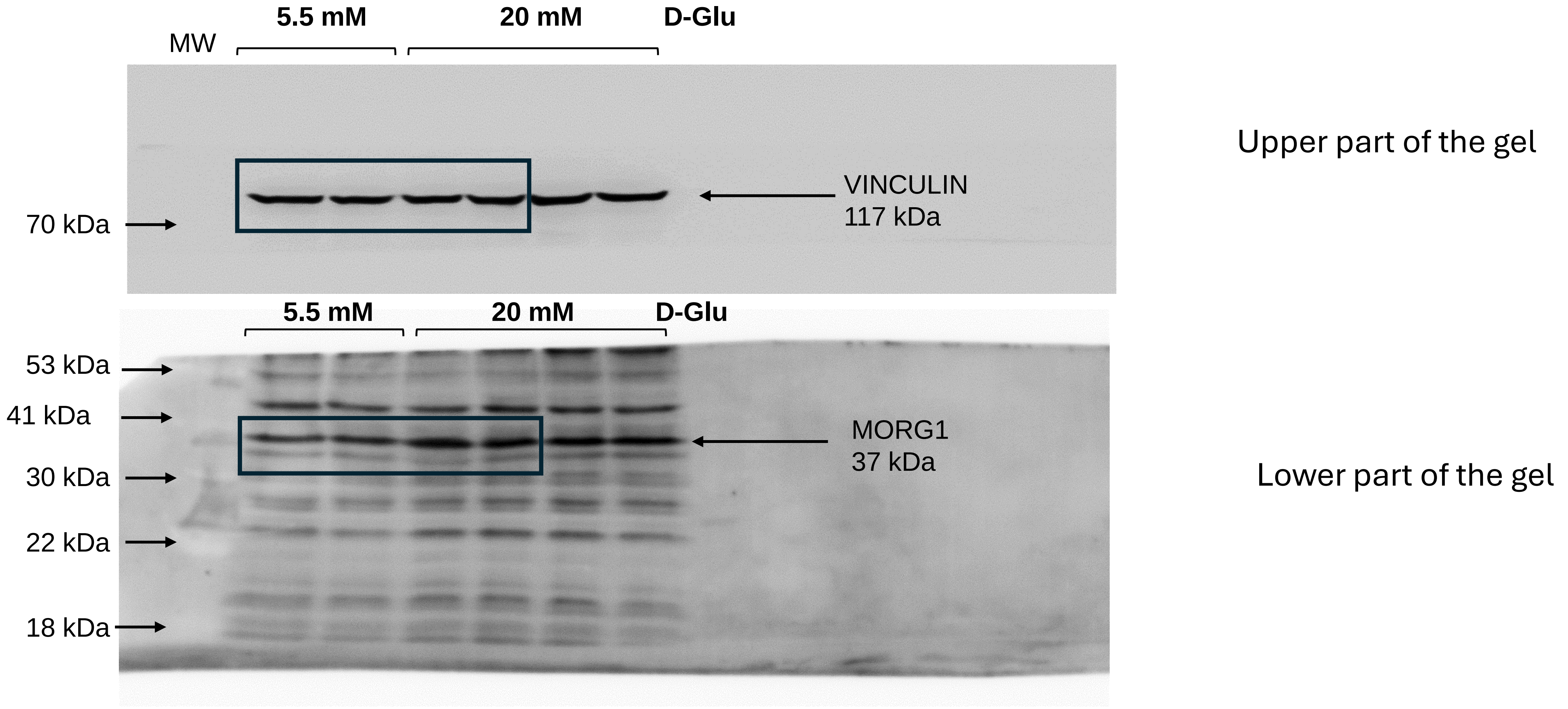

**Figure 6 d)**
Western Blots **12 h treatments** WB # 2

The SDS-PAGE gel was transferred on a PVDF membrane and
- the upper part was probed with an antibody against VINCULIN;
- the lower part was probed with an antibody against MORG1.

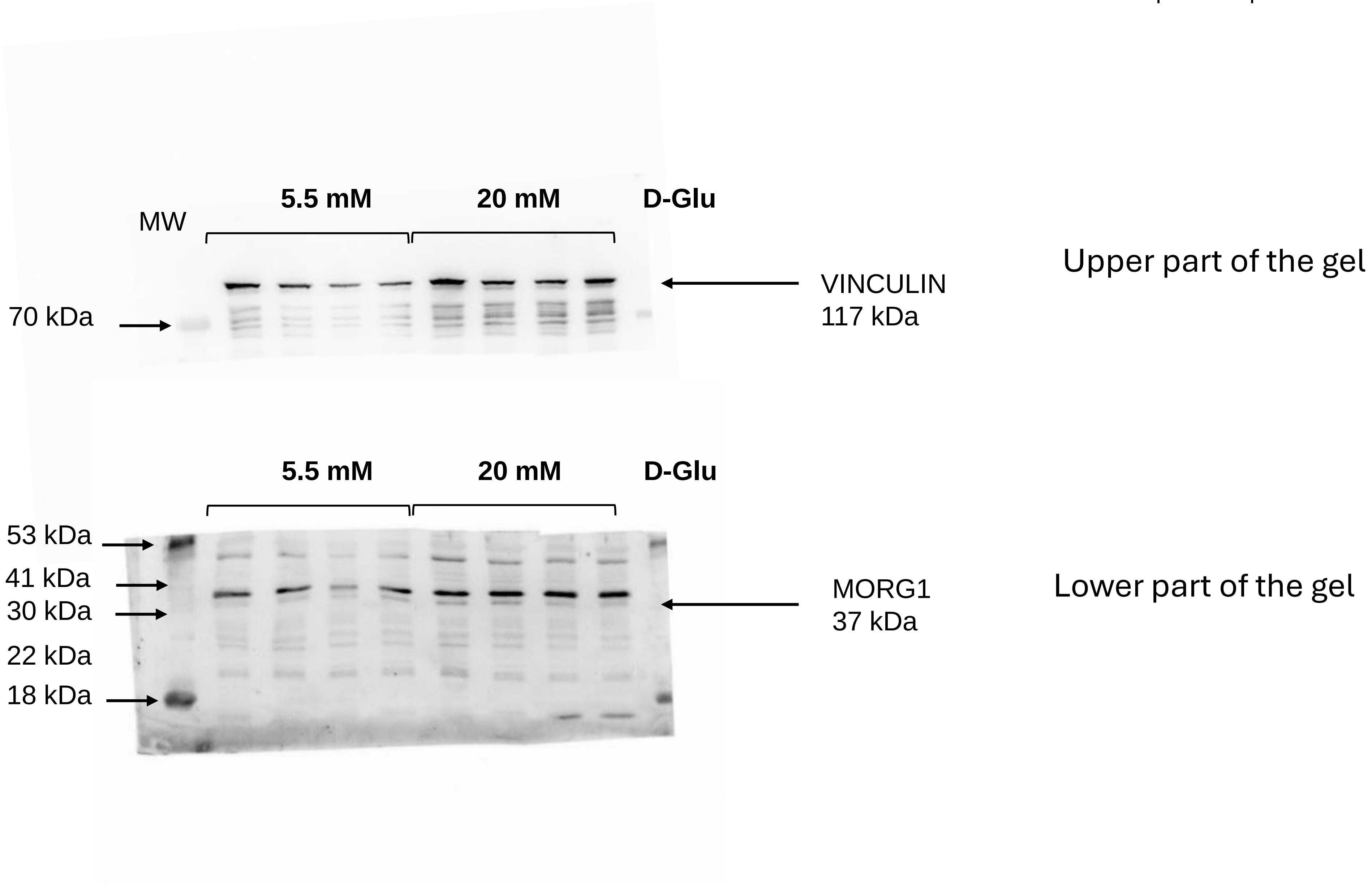

**Figure 8 a)**
Western Blots

WB # 1

The SDS-PAGE gel was transferred on a PVDF membrane and
- the upper part was probed with an antibody against VINCULIN;
- the lower part was probed with an antibody against MORG1.

**- The bands of MORG1 and VINCULIN expressions from WB #1 are shown as representative images in Fig. 8a)**

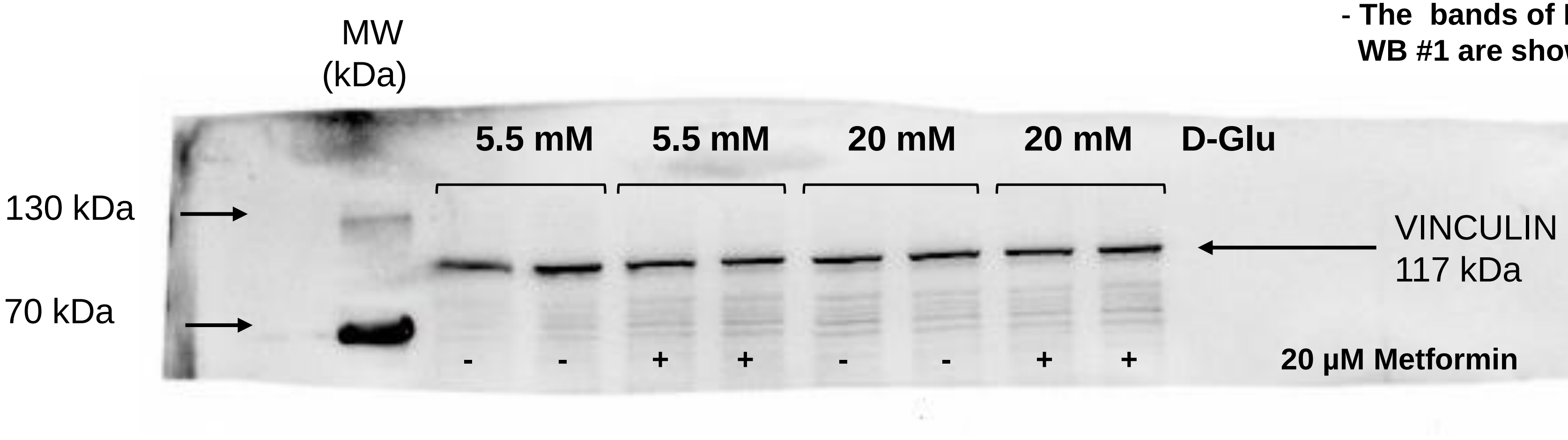


Upper part of the gel

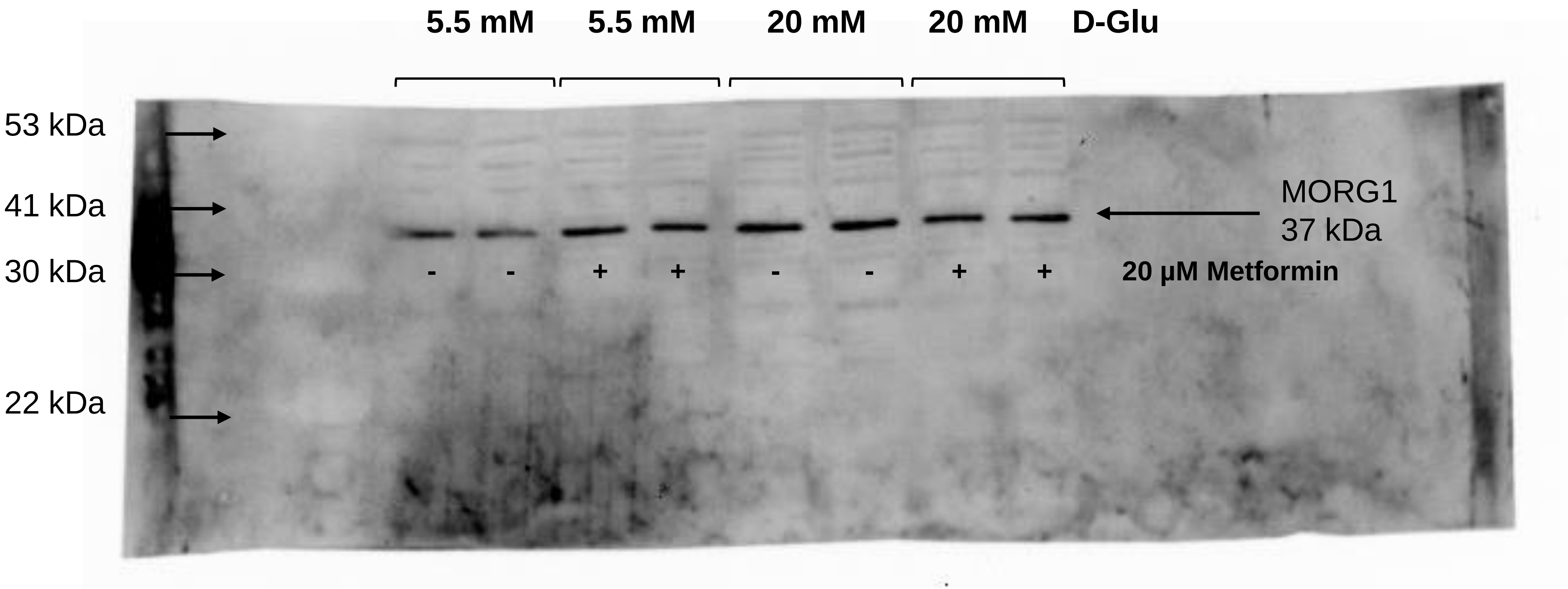


Lower part of the gel

**Figure 8 a)**
Western Blots

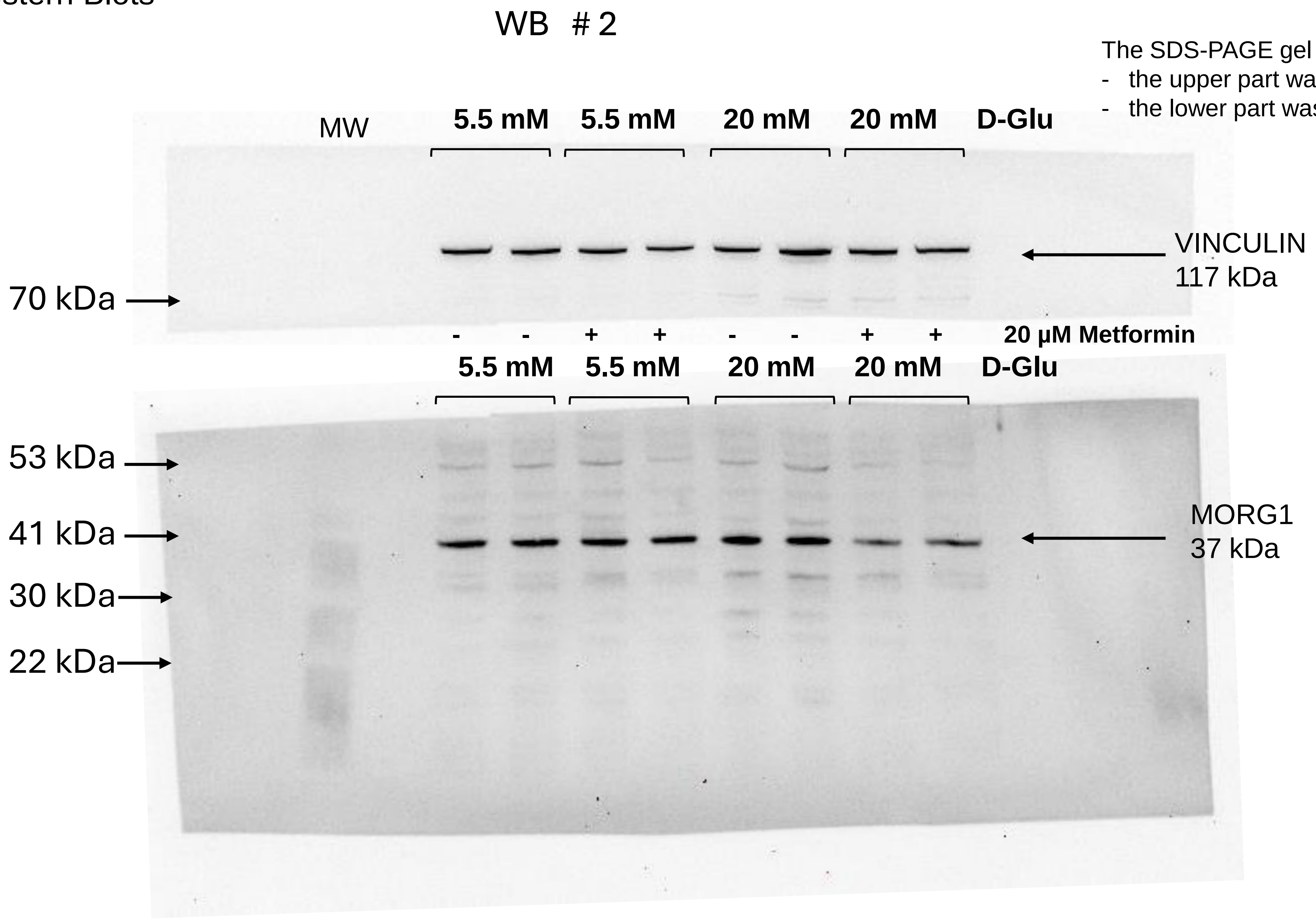


The SDS-PAGE gel was transferred on a PVDF membrane and
- the upper part was probed with an antibody against VINCULIN;
- the lower part was probed with an antibody aginst MORG1.

Upper part of the gel

Lower part of the gel

**Figure 9 a)**
Western Blots

WB # 1

The SDS-PAGE gel was transferred on a PVDF membrane and
- the upper part was probed with an antibody against VINCULIN;
- the lower part was probed with an antibody against MORG1.

**- The bands of MORG1 and VINCULIN expressions from WB #1 are shown as representative images in Fig. 9a)**

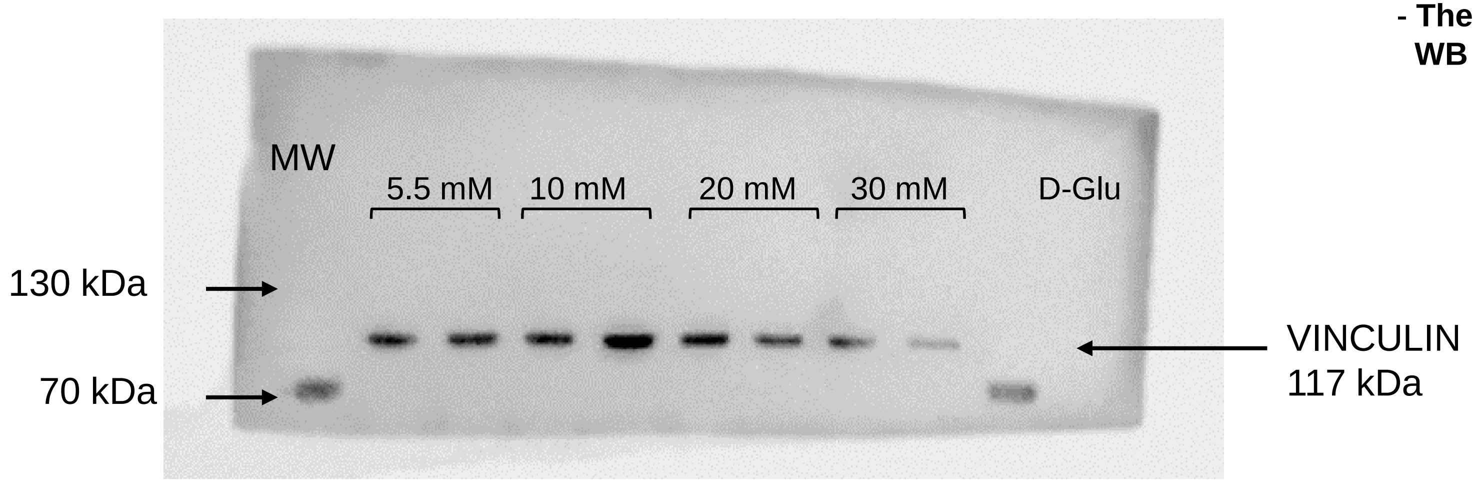


Upper part of the gel

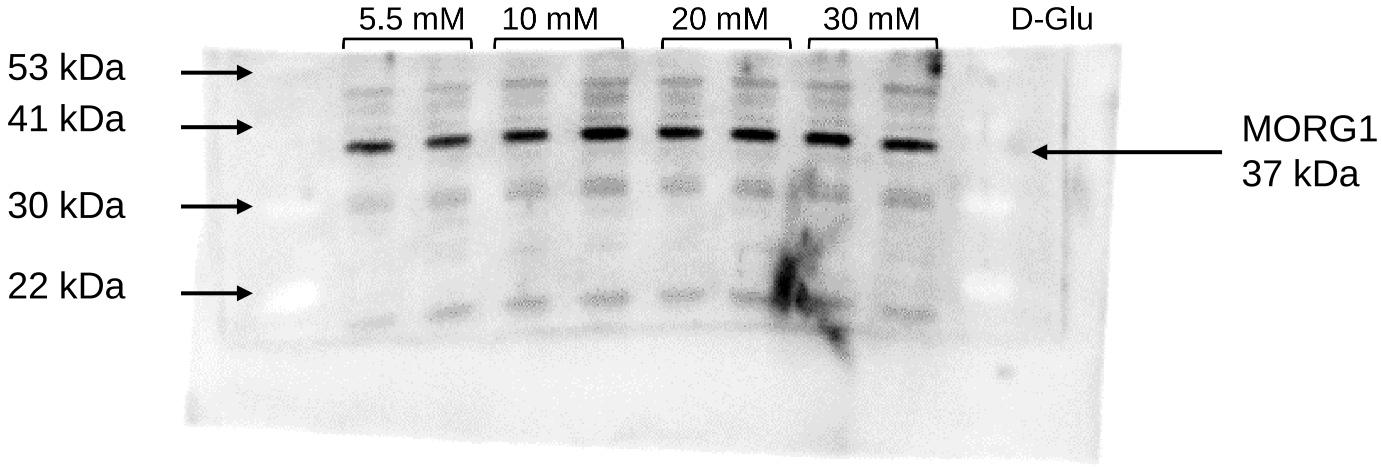


Lower part of the gel

**Figure 9 a)**
Western Blots

The SDS-PAGE gel was transferred on a PVDF membrane and
- the upper part was probed with an antibody against MORG1.
- part was probed with an antibody against VINCULIN;
- the lower

WB # 2

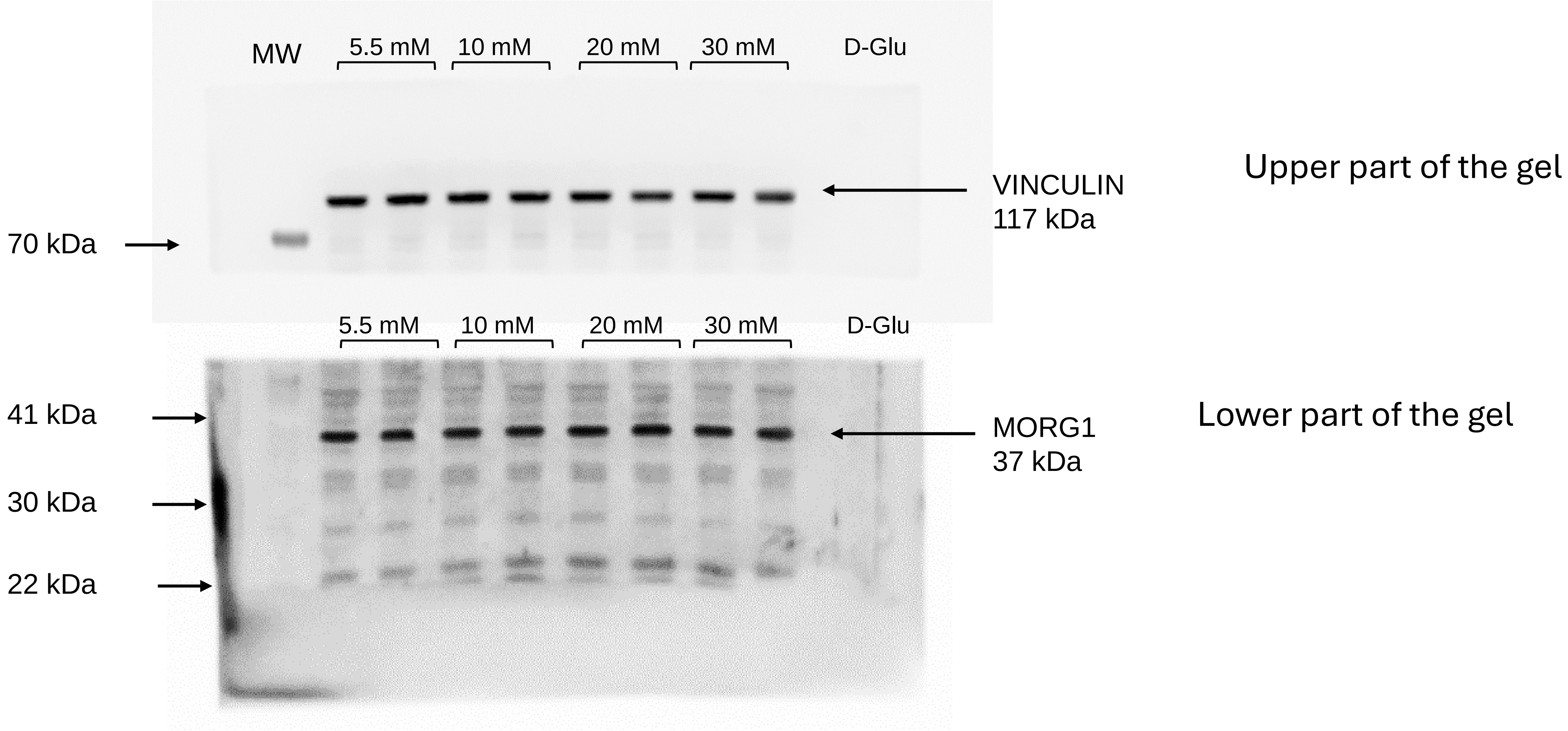

**Figure 9 a)**
Western Blots

The SDS-PAGE gel was transferred on a PVDF membrane and
- the upper part was probed with an antibody against VINCULIN;
- the lower part was probed with an antibody against MORG1.

- **The labeled lanes from WB #3 were included to the calculations of MORG1 expression in HEK293 cells shown on the corresponding graph**

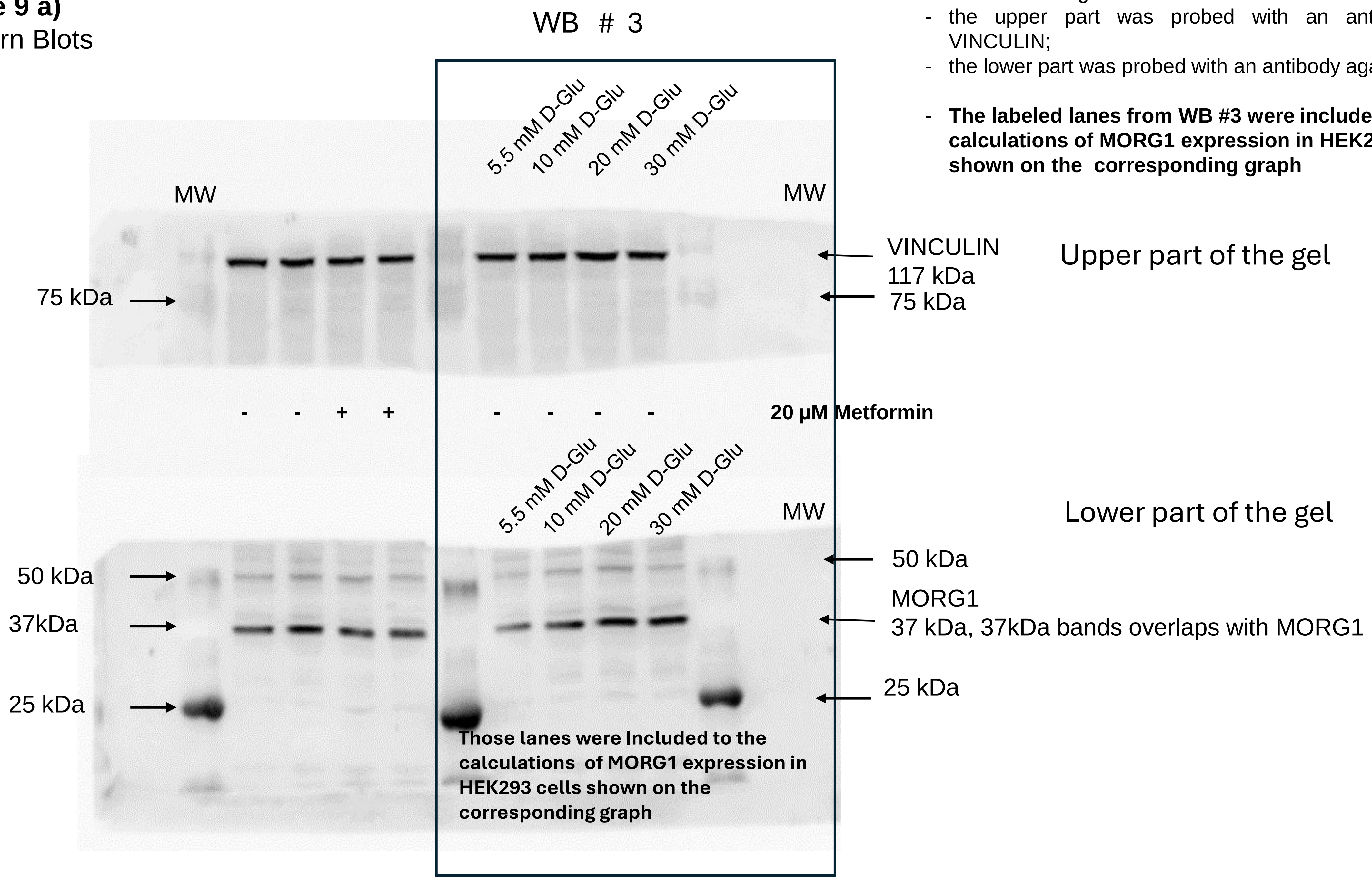

**Figure 10 a)**
Western Blots

The SDS-PAGE gel was transferred on a PVDF membrane and
- the upper part was probed with an antibody against VINCULIN;
- the lower part was probed with an antibody against MORG1.

**- The bands of MORG1 and VINCULIN expressions from WB #1 are shown as representative images in Fig. 10a)**

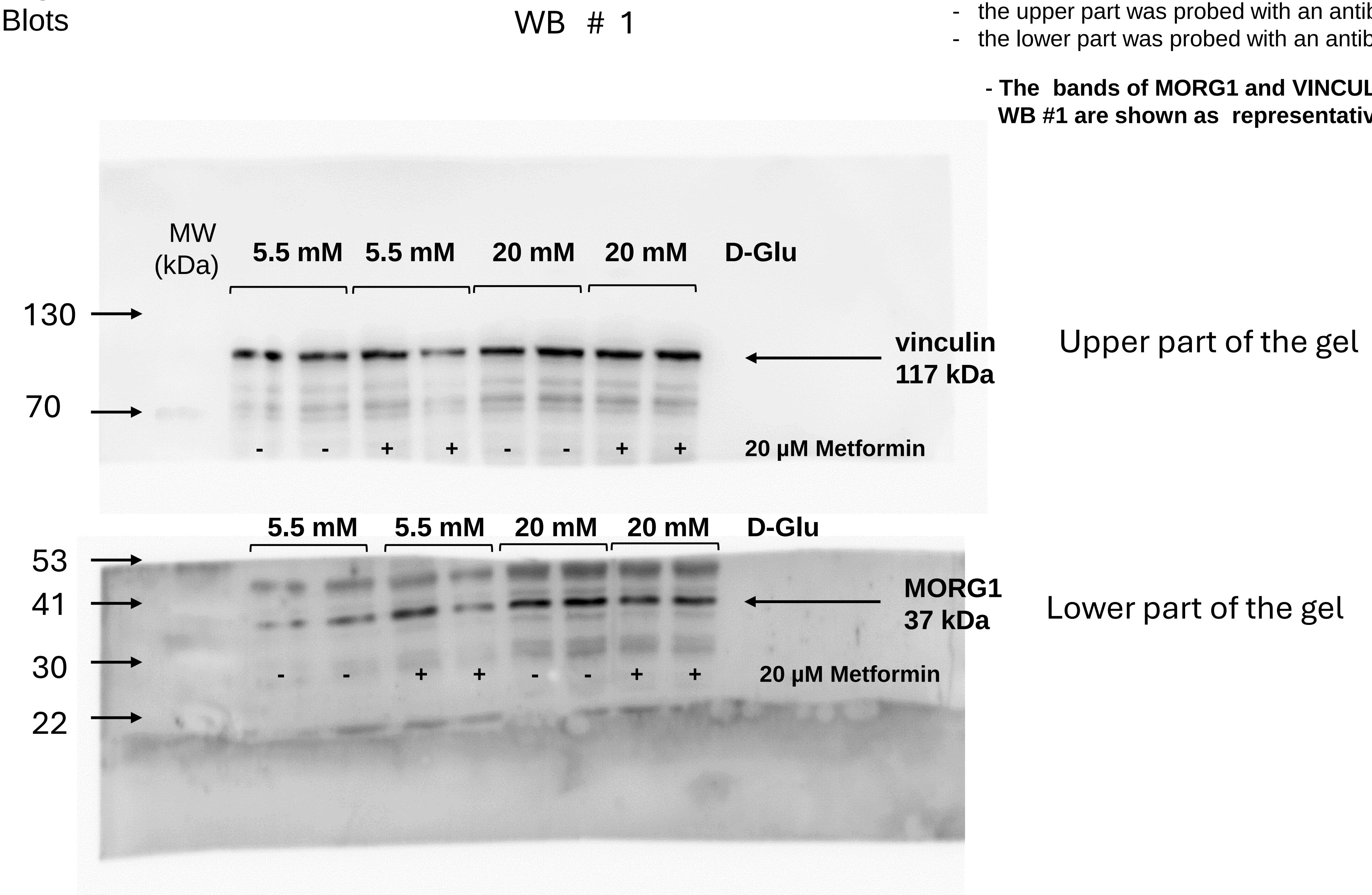

**Figure 10 a)**
Western Blots

The SDS-PAGE gel was transferred on a PVDF membrane and
- the upper part was probed with an antibody against VINCULIN;
- the lower part was probed with an antibody against MORG1.

- All bands were included to the calculations of the MORG1 expression shown on the corresponing graph

WB # 2

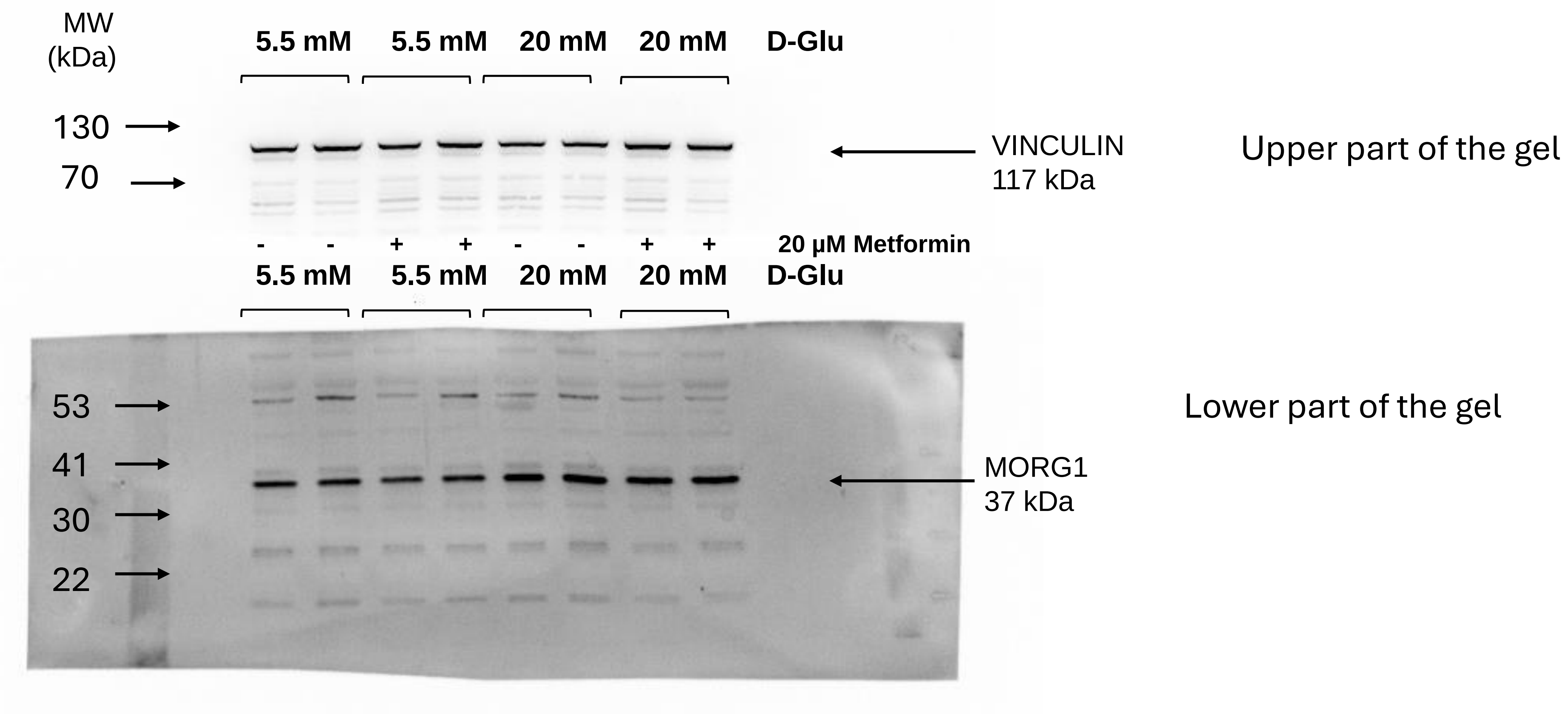

**Figure 10 a)**
Western Blots

The SDS-PAGE gel was transferred on a PVDF membrane and
- the upper part was probed with an antibody against VINCULIN;
- the lower part was probed with an antibody against MORG1.

**- The labeled lanes from WB #3 were included to the calculation of of MORG1 expression in HEK293 cells shown on the corresponding graph**

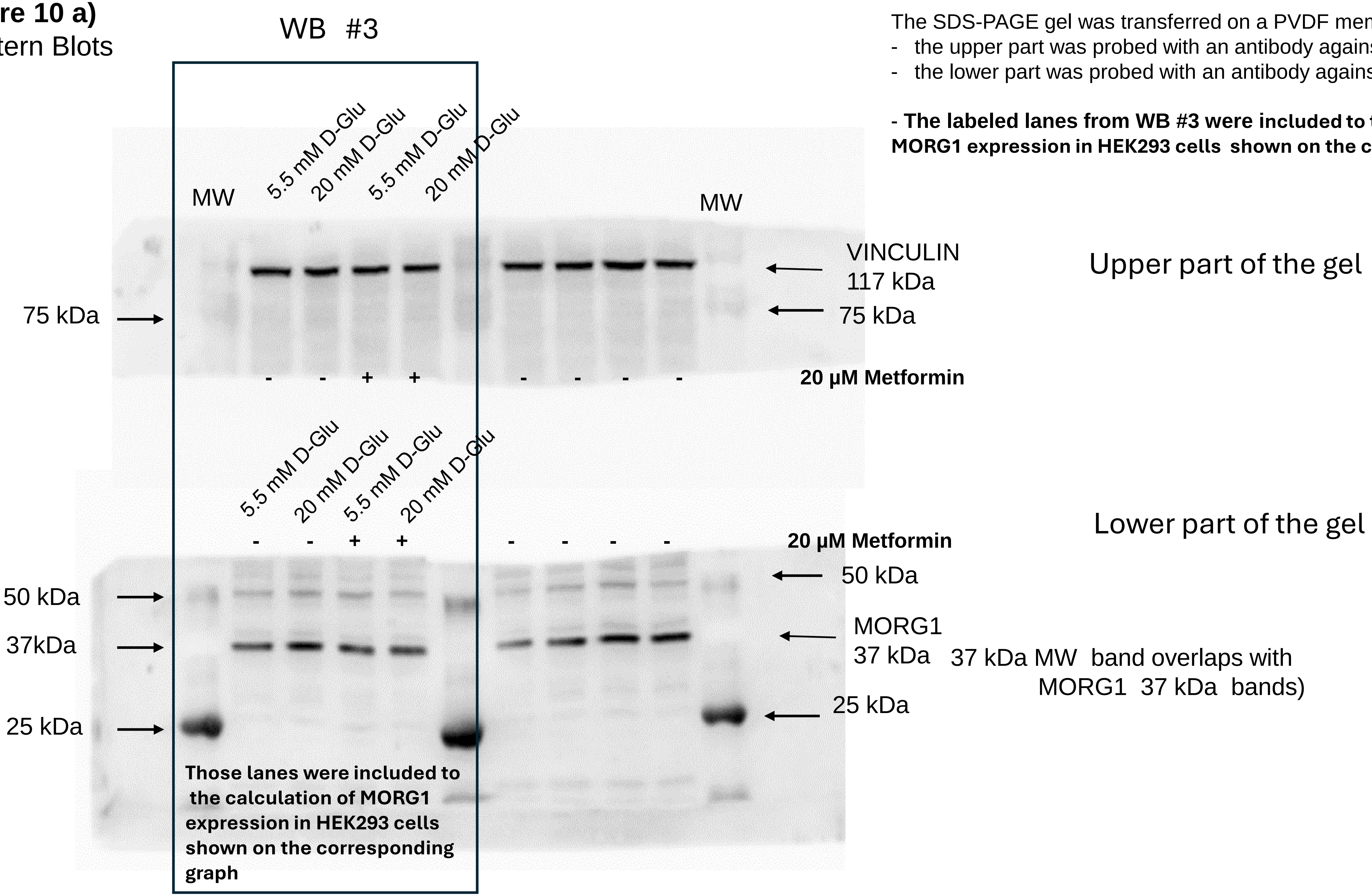

**Figure 11 b)**
Western Blots

WB from TKPTS cells lysates

The SDS-PAGE gel was transferred on a PVDF membrane and
- the upper part was probed with an antibody against VINCULIN;
- the lower part was probed with an antibody against MORG1.

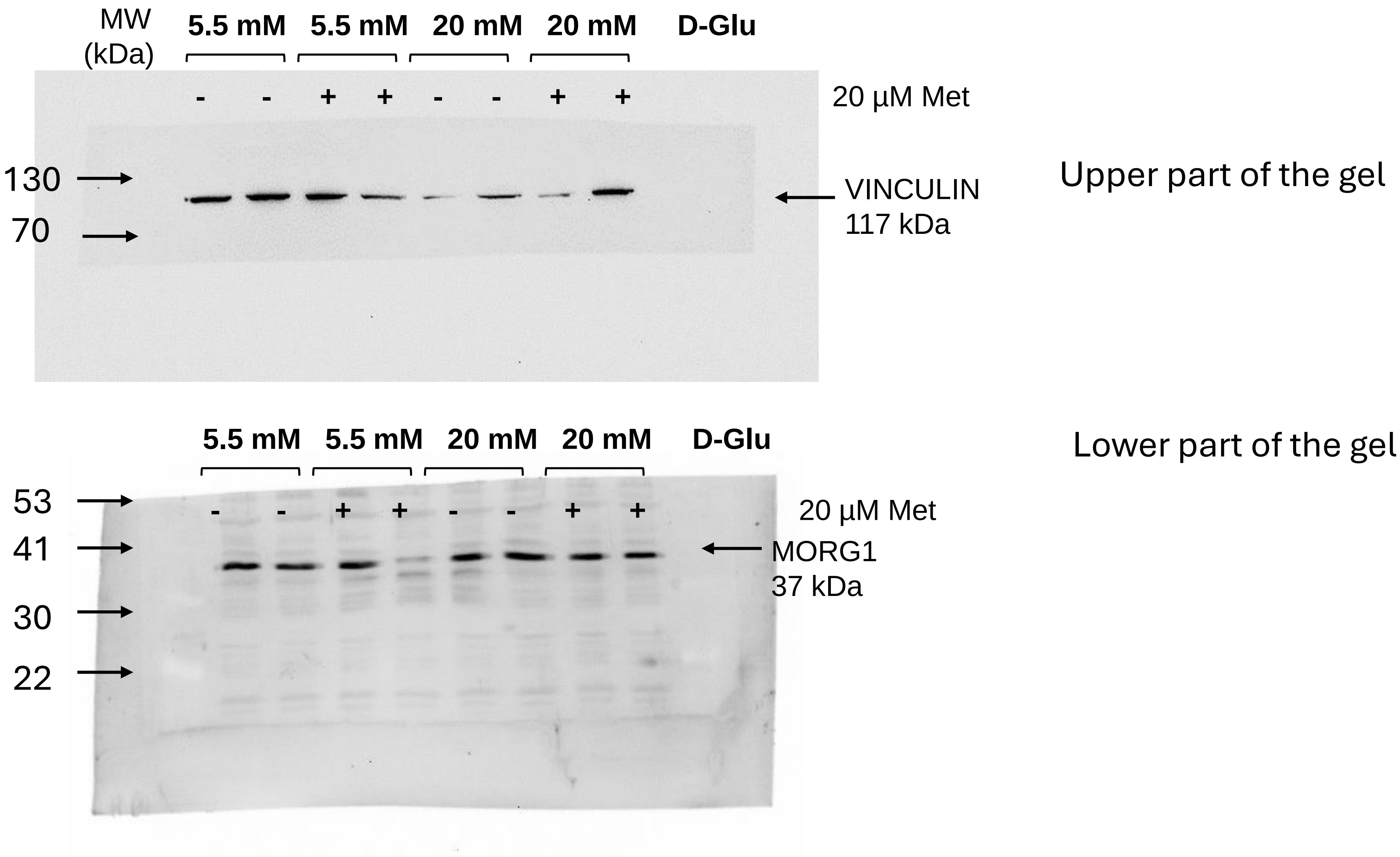

**Figure 13 a)**
Western Bolts

WB from HEK293 cells lysates

The SDS-PAGE gel was transferred on a PVDF membrane and
- First was probed with an antibody against pAMPK Thr172
- after that the membrane was stripped and re-probed with an antibody against VINCULIN;

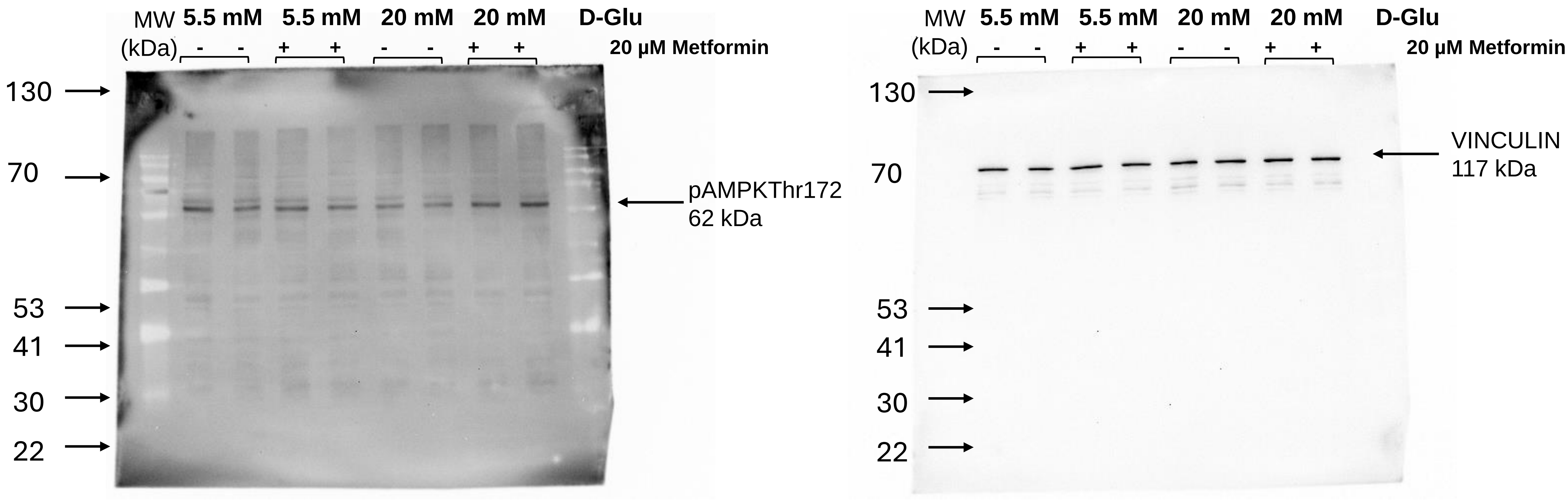

**Figure 13 b)**
Western Bolts

WB from TKPTS cells lysates

The SDS-PAGE gel was transferred on a PVDF membrane and
- First was probed with an antibody against pAMPK Thr172
- after that the membrane was stripped and re-probed with an antibody against VINCULIN;

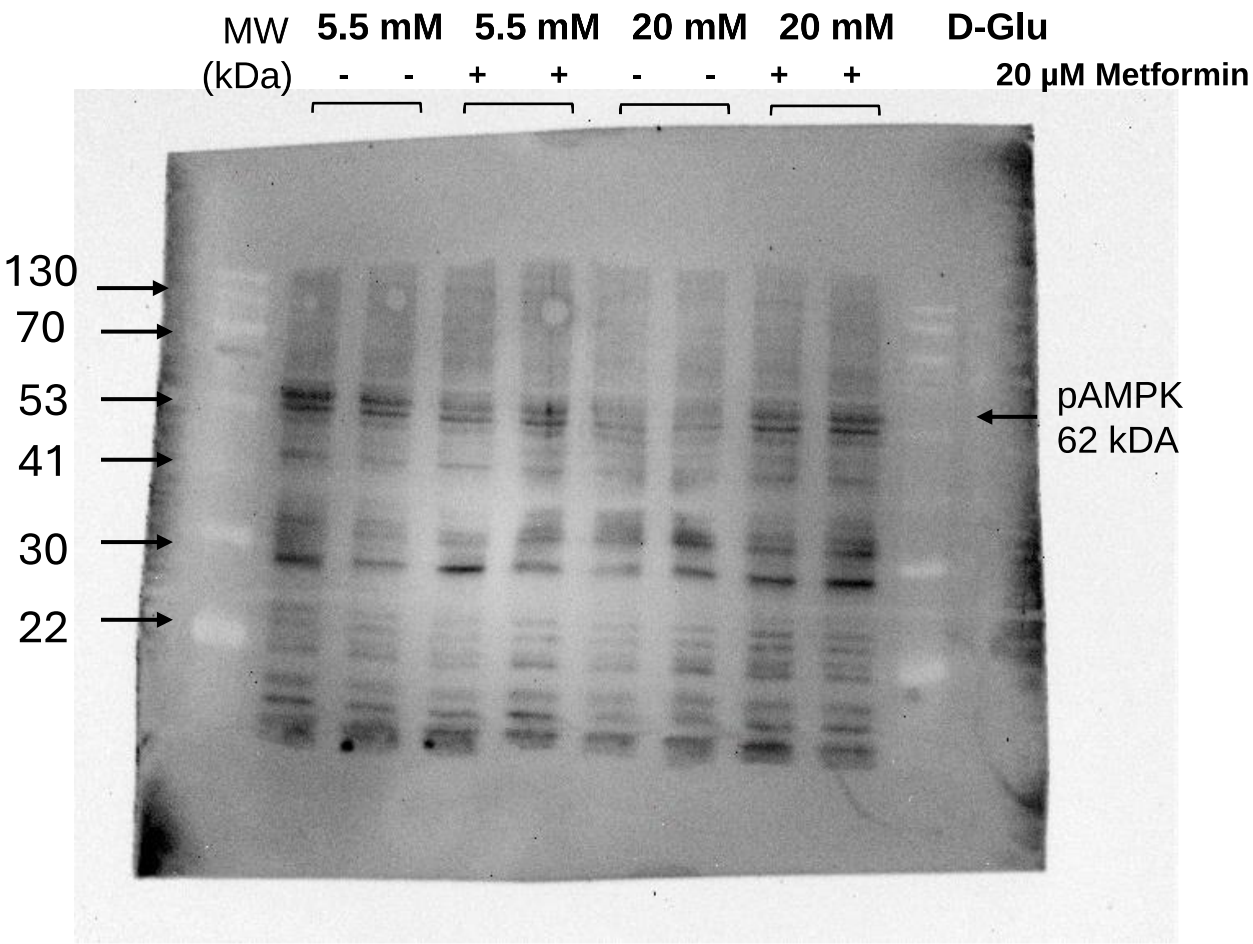


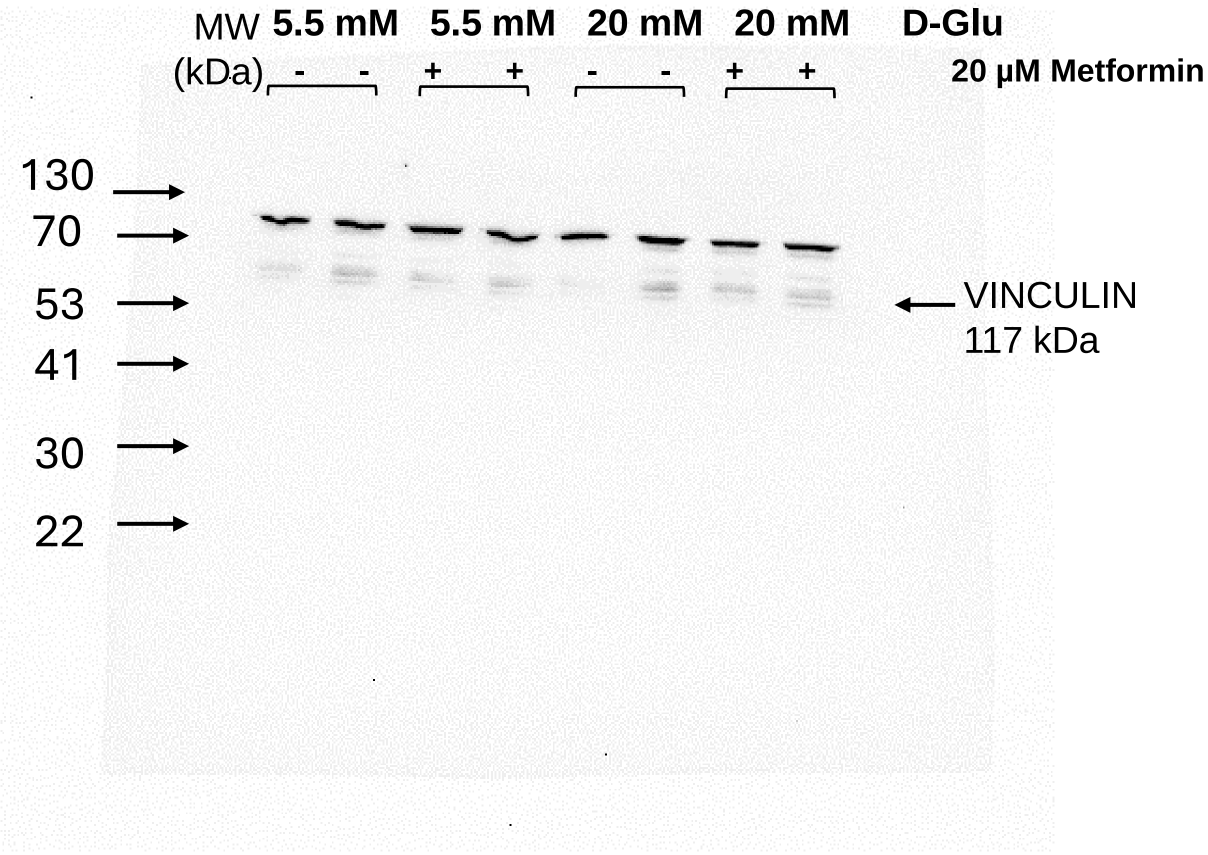

Supplement: Supplementary file 1 [file cimb-48-00934-s001.zip › cimb-4447042-supplementary.pdf]
